# Discovery of Icotinib-1,2,3-Triazole Derivatives as IDO1 Inhibitors

**DOI:** 10.3389/fphar.2020.579024

**Published:** 2020-09-30

**Authors:** Long-fei Mao, Yu-wei Wang, Jie Zhao, Gui-qing Xu, Xiao-jun Yao, Yue-Ming Li

**Affiliations:** ^1^State Key Laboratory of Medicinal Chemical Biology, College of Pharmacy and Tianjin Key Laboratory of Molecular Drug Research, Nankai University, Tianjin, China; ^2^School of Chemistry and Chemical Engineering, Henan Engineering Research Center of Chiral Hydroxyl Pharmaceutical, Henan Normal University, Xinxiang, China; ^3^College of Pharmacy, Shaanxi University of Chinese Medicine, Xi’an-Xianyang New Economic Zone, Xianyang, China; ^4^State Key Laboratory of Quality Research in Chinese Medicine/Macau Institute for Applied Research in Medicine and Health, Macau University of Science and Technology, Macau, China

**Keywords:** icotinib, 1,2,3-triazole, indoleamine 2,3-dioxygenase 1, inhibitor, immunotherapy

## Abstract

Tumor immunotherapy is considered to be a highlight in cancer treatment in recent years. Indoleamine 2,3-dioxygenase 1 (IDO1) is closely related to the over expression of many cancers, and is therefore a promising target for tumor immunotherapy. To search for novel IDO1-targeting therapeutic agents, 22 icotinib-linked 1,2,3-triazole derivatives were prepared and evaluated for their inhibitory activity against IDO1. The structures of the prepared compounds were confirmed with^1^H NMR, ^13^C NMR and HR MS. IDO1 inhibitory activity assay results indicated that 10 of those compounds showed remarkable inhibitory activity against IDO1, among which compound **a17** was the most potent with IC_50_value of 0.37 μM. The binding model between the prepared compounds and IDO1 was studied with molecular modeling study. The current study suggested that icotinib-1,2,3-triazole derivatives could be used as potential inhibitors that preferentially bind to the ferrous form of IDO1 through the formation of coordinate bond with the haem iron.

**Graphical Abstract:**
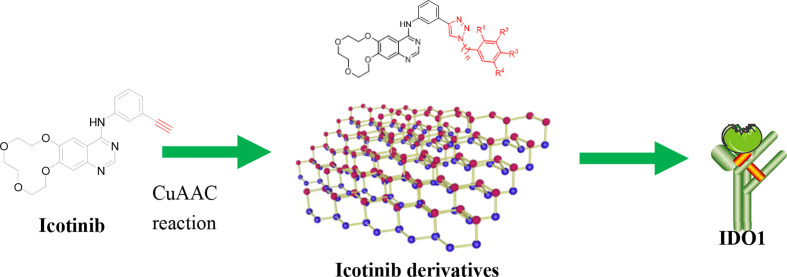


## Introduction

Tumor immunotherapy is an emerging field in tumor treatment. Studies show that indoleamine 2,3-dioxygenase 1 (IDO1) is the initial and rate-limiting enzyme that catalyzes the metabolism of tryptophan along the kynurenine pathway outside the human liver (Chen et al., 2019), and plays an important role in regulating the body’s innate and adaptive immunity by catalyzing tryptophan metabolism (Takikawa et al., 1986; Takikawa, 2005). In the tumor microenvironment, tumor cells can induce IDO1 over expression, which causes the depletion of local tryptophan and the accumulation of metabolites such as kynurenine, thereby activating GCN2 and AHR signaling pathways, inhibiting T cell proliferation, and inducing apoptosis (Muller et al., 2005). Additionally, the original T cells are stimulated to differentiate into regulatory T cells, thus mediating tumor immune escape (Efimov et al., 2011). Over expression of IDO1 has been found in a variety of malignant tumors, such as ovarian cancer, pancreatic cancer, and non-small cell lung cancer. Therefore, IDO1 inhibitors once attracted considerable attention as potential agents for cancer treatment.

**Figure 1:**
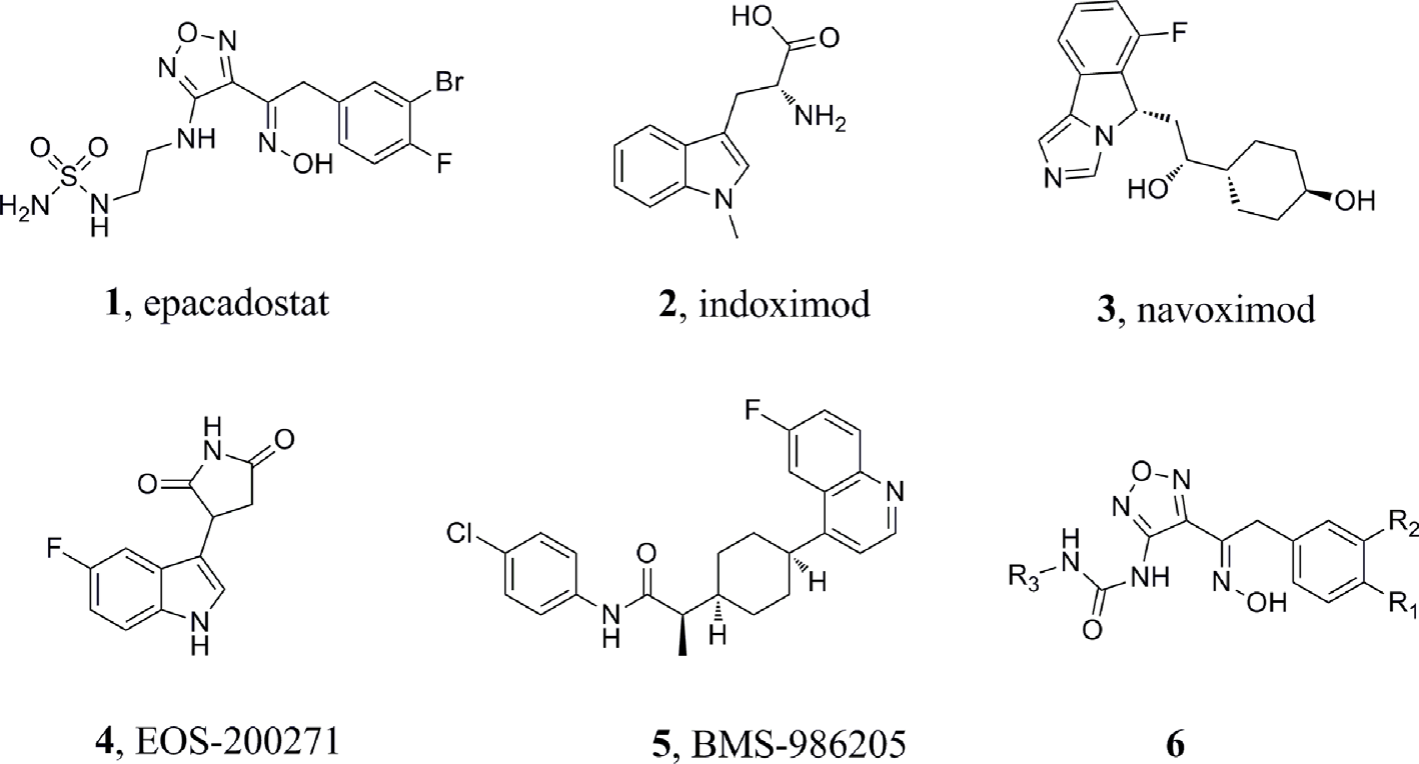
Chemical structures of sixIDO1 inhibitors.

**Figure 2:**
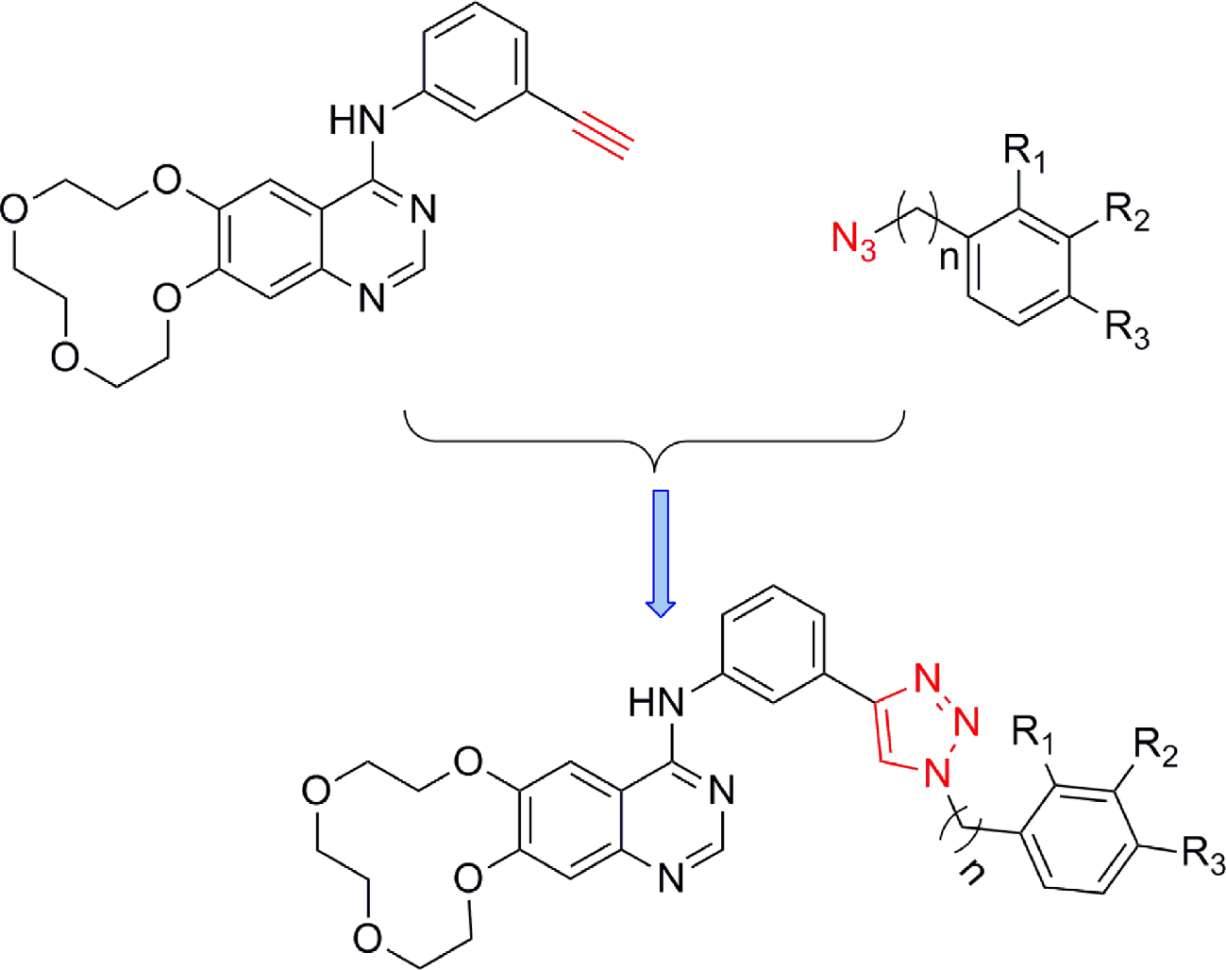
The reaction routes to1,2,3-triazole-linkedicotinibderivatives.

**Figure 3:**
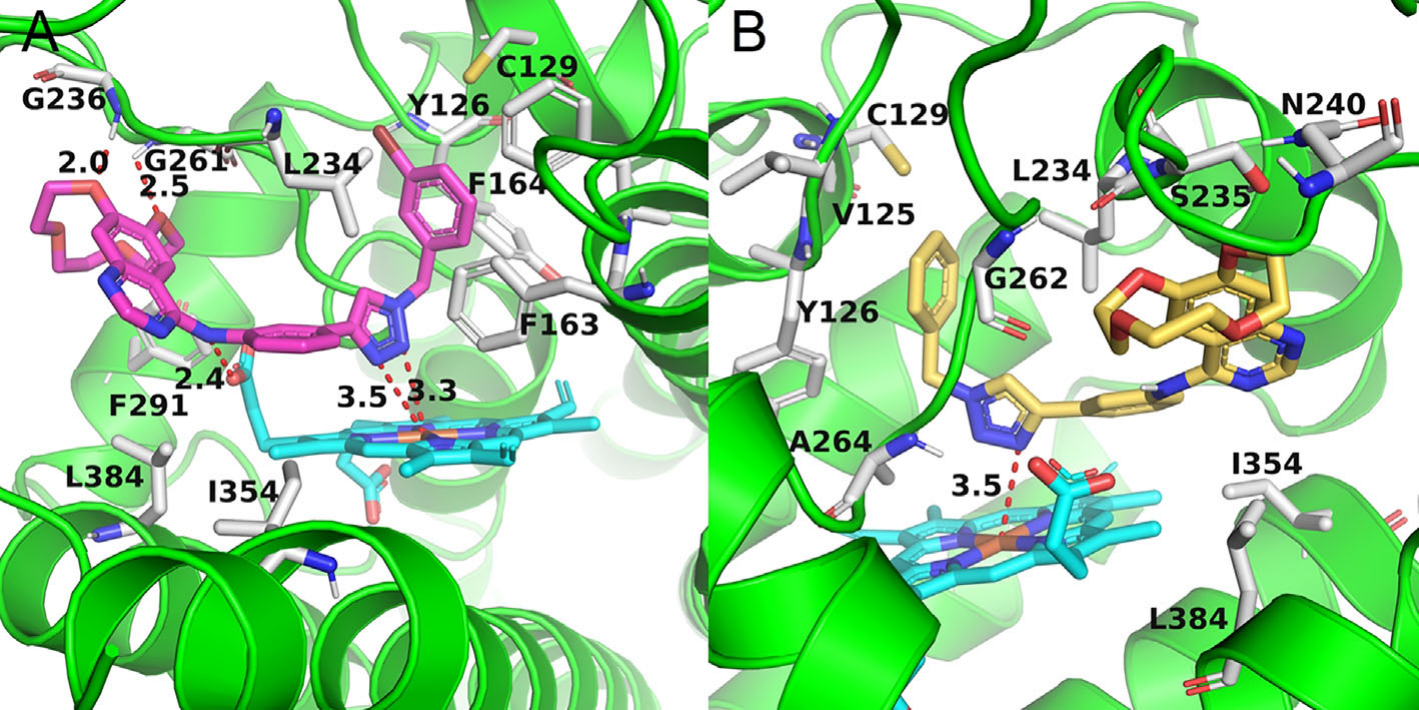
The binding mode of compounds in complex with IDO1. Theprotein is represented by a green cartoon, while compound **a17** (pink, **A**) and compound **a18** (yellow, **B**) are represented as sticks. The hydrogen bonds are colored in red dash.

**Figure 4:**
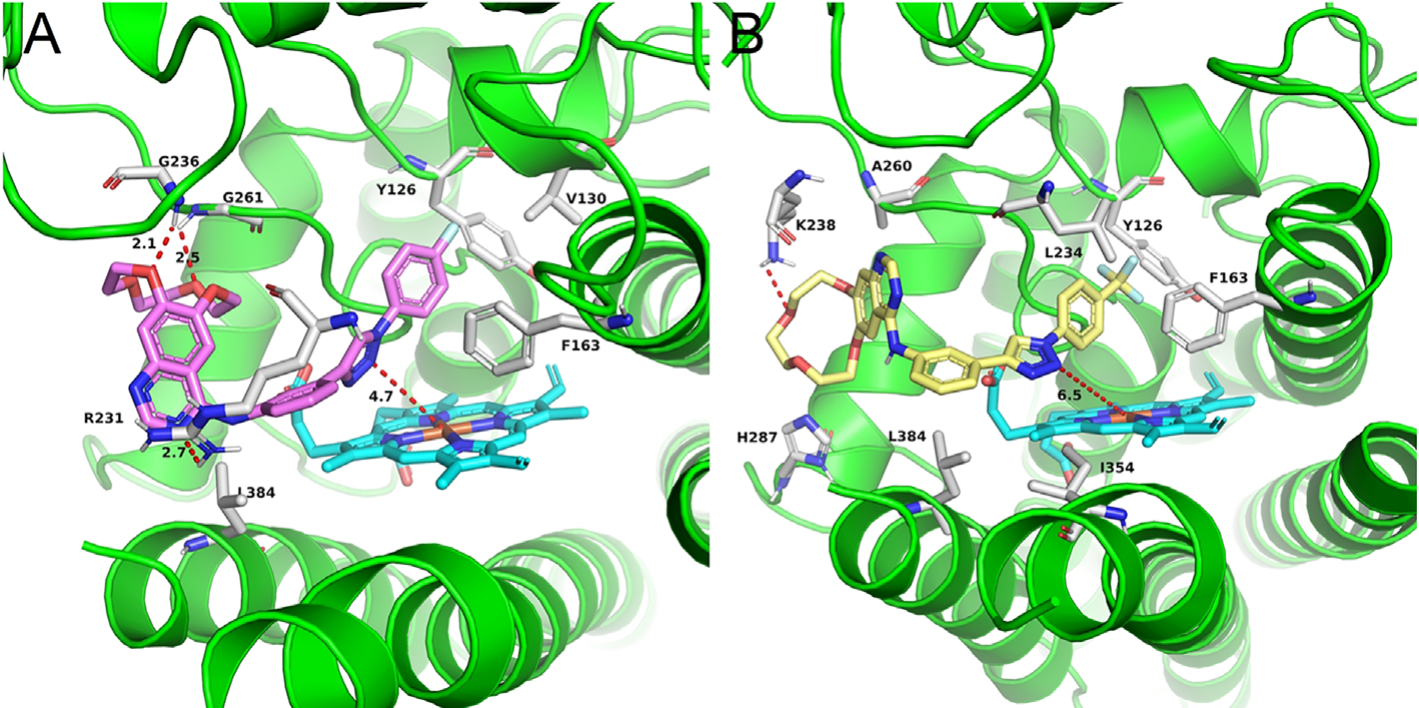
The binding mode of compounds in complex with IDO1. Theprotein is represented by a green cartoon, while compound **a3** (pink, **A**) and compound **a12** (yellow, **B**) are represented as sticks. The hydrogen bonds are colored in red dash.

Several candidates are currently undergoing clinical trials, but none of these has been approved so far, suggesting that the identification of potent and clinically useful IDO1 inhibitors is an open challenge. For example, epacadostat (**Figure 1**, **1**, INCB024360) (Yue et al., 2017), indoximod (**Figure 1**, **2**, 1-methyl-D-tryptophan) (Soliman et al., 2009), navoximod (**Figure 1**, **3**, NLG-919) (Kumar et al., 2019), EOS-200271 (**Figure 1**, **4**, PF-06840003) (Crosignani et al., 2017), and BMS-986205 (**Figure 1**, **5**) have been are currently being tested in human clinical trials. Epacadostat, developed by Incyte, is the first highly effective and highly selective oral IDO1 inhibitor (Morgan et al., 2008; Lin et al., 2016; Lewis-Ballester et al., 2017; Yue et al., 2017). It can effectively restore the anti-tumor immune response in human Hela cells treated with IFN-γ. *Via* reversing tumor-associated immunosuppression, it can effectively suppress kynurenineproduction. Epacadostat also increases IFN-γ production, promotes the growth of natural killer (NK) and T cells, and reduces the number of converted regulatory T cells (Tregs) (Dounay et al., 2015). Based on the promising results in Phase 1/2 studies, epacadostat proceeded to a Phase 3 trial (ECHO-301) in combination with pembroluzimab in the treatment of metastatic melanoma. Recent results coming from the pivotal Phase 3 trial of ECHO-301 have shown no indication that epacadostat provides an increased benefit compared to pembrolizumab alone, questioning the effectiveness of IDO1 inhibitors. This failure led to the interruption of other Phase III trials and the reconsideration of whether some elements had been neglected in the landscape of IDO1 inhibitors (Serafini et al., 2020). Study showed that the key group playing the active role in the epacadostat molecule was the oxadiazole structure. The epacadostat molecule entered the heme pocket of the IDO1, and the oxadiazole structure located directly above the Fe ion in the heme and then interacted with it. Based on this, a new type of IDO1 inhibitor which linked urea groups to the oxadiazole structure was developed (Wu et al., 2017; Song et al., 2020) (**Figure 1**, **6**) and the compounds showed submicromolar level of IC_50_ against IDO1.

1,2,3-Triazole, an *N*-heterocyclic building block, played a significant role in drug design and synthesis (Majeed et al., 2013). Many compounds containing the 1,2,3-triazole unit exhibited good activities against inflammation, cancer, and microbes (De Souza et al., 2020). Moreover, copper(I)-catalysedazide-alkyne cycloaddition (CuAAC) reaction, a convenient and regiospecific approach to 1,4-disubstituted triazoles (Thomopoulou et al., 2015), has aroused great interest among the researchers and has been widely used in the preparation of different bioactive molecules (Hong et al., 2010). Compounds containing 1,2,3-triazole moiety showed good bioactivities such as antitumor or antibacterial activity (Röhrig et al., 2012; Mao et al., 2017). Furthermore, according to the literature, compounds containing 1,2,3-triazole possessed promising IDO1 inhibition (IC_50_ = 12.6 μM).

Encouraged by these results, we decided to study the bioactivity of compounds bearing different 1,2,3-triazole groups. At first, icotinib was chosen as the starting point. This compound has been clinically used in China for the treatment of NSCLC (Yang et al., 2017; Liang et al., 2018; Zhang et al., 2018). We envisioned that introducing 1,2,3-triazole structure into the molecule *via* conventional click reaction would give compounds with additional benefit by the 1,2,3-triazole group, and this twin drug approach will combine the advantages of both EGFR-TKI and IDO1 inhibitors. Herein, we wish to present our preliminary results on the preparation of the 1,2,3-triazole derivatives and their *in vitro* inhibitory activity against IDO1.

## Chemistry

The synthetic strategy for the preparation of the target compounds is illustrated in **Figure 2**. Copper(I)-catalysed azide–alkyne cycloaddition between icotinib and different azido compounds afforded the target compounds **a1**–**a22**. The reaction conditions of these steps were convenient and easy to control. The structures of some key intermediates and all target compounds were confirmed by nuclear magnetic resonance and high-resolution mass spectrometry experiments.

## Experimental Protocols

### Materials and Chemistry

Icotinib-1,2,3-triazole derivatives were in-house synthesized. Icotinib and Aryl-azido compounds were purchased from Acros Organics (Morris Plains, NJ, USA). All reagents and solvents obtained from commercially available source were used without further treatment. ^1^H NMR and ^13^C NMR spectra were acquired in DMSO-d_6_ or CDCl_3_ solution with a Bruker 600 spectrometer. Chemical shifts (δ) were given in parts per million with tetramethylsilane as internal reference and coupling constants were expressed in hertz. High-resolution mass spectra (HRMS) measurements were carried out using an Bruker MicrOTOF-Q II mass spectrometer.

Hela cell line, DMEM medium and fetal bovine serum were purchased from ATCC (Virginia, USA). Recombinant human IFN-γ was purchased from R&D systems (Emeryville, CA, USA). The 3.05 Ntrichloroacetic acid, 4-(dimethylamino)benzaldehyde and acetic acid were purchased from Sigma Aldrich (St. Louis, MI, USA).

### General Procedure for the Synthesis of Analogues a1–a22

#### General Procedure for Preparation of Compound **a1–a22**

Aryl-azido (1.2 mmol) and icotinib (1.0 mmol) were added to 15 ml mixed solvent (water: *tert*-butanol =2:1). The reaction was carried out with copper sulfate pentahydrate (0.1 mmol) and sodium ascorbate (0.2 mmol) at 80°C. After completion of the reaction (monitored by TLC), the mixture was extracted with dichloromethane (15 ml×3). The combined organic phase was washed successively with water and brine, dried over sodium sulfate and concentrated *in vacuo*. The residue was purified wiht column chromatography (CH_2_Cl_2_/MeOH=20:1) to give the desired compound a.

*{3-[1-(3-Fluoro-phenyl)-1H-[1,2,3]triazol-4-yl]-phenyl}-(7,8,10,11,13,14-hexahydro-6,9,12,15-tetraoxa-1,3-diaza-cyclododeca[b]naphthalen-4-yl)-amine(**a1**):* Yellow solid, Purity 96%; ^1^H NMR (600 MHz, DMSO-*d_6_*): *δ* 9.66 (s, 1H), 9.38 (s, 1H), 8.56 (s, 1H), 8.44 (s, 1H), 8.23 (s, 1H), 7.98 (d, *J*=7.9 Hz, 1H), 7.91 (dd, *J_1_* = 19.0_ _*Hz*, *J_2_* = 9.0* Hz*, 2H), 7.70 (dd, *J_1_* = 14.8_ _*Hz*, *J_2_* = 7.8_ _*Hz*, 1H), 7.65 (d, *J*=7.5 Hz, 1H), 7.53 (t, *J*=7.8 Hz, 1H), 7.39 (t, *J*=8.3 Hz, 1H), 7.33 (s, 1H), 4.32 (d, *J*=12.4 Hz, 4H), 3.79 (d, *J*=19.9 Hz, 4H), 3.65 (s, 4H); ^13^C NMR (150 MHz, DMSO-*d_6_*): 163.8, 162.1, 157.1, 156.5, 153.8, 150.3, 147.9, 140.6, 138.4, 132.4, 130.8, 129.7, 122.6, 121.0, 120.3, 119.3, 116.4, 115.8, 112.1, 110.7, 108.1, 107.9, 73.4, 70.9, 70.9, 70.5, 69.3, 68.9; HR MS (ESI) *m/z*: calcd for C_28_H_26_O_4_N_6_F [M+H]^+^ 529.1994, found 529.2000.

*{3-[1-(4-Chloro-phenyl)-1H-[1,2,3]triazol-4-yl]-phenyl}-(7,8,10,11,13,14-hexahydro-6,9,12,15-tetraoxa-1,3-diaza-cyclododeca[b]naphthalen-4-yl)-amine (**a2**)*: Brown solid, Purity 97%; ^1^H NMR (600 MHz, DMSO-*d_6_*): *δ* 9.37 (s, 1H), 8.59 (s, 1H), 8.44 (s, 1H), 8.30 (s, 1H), 8.04 (d, *J*=7.7 Hz, 2H), 7.95 (d, *J*=8.0 Hz, 1H), 7.74 (d, *J*=7.7 Hz, 2H), 7.67 (d, *J*=7.6 Hz, 1H), 7.53 (t, *J* =7.8 Hz, 1H), 7.34 (s, 1H), 4.33 (d, *J*=13.2 Hz, 4H), 3.79 (d, *J* =22.6 Hz, 4H), 3.65 (s, 4H); ^13^C NMR (150 MHz, DMSO-*d_6_*): 157.3, 156.7, 150.4, 147.9, 140.4, 135.9, 133.6, 130.8, 130.4, 129.6, 126.5, 125.7, 122.7, 122.1, 121.2, 119.5, 110.9, 73.4, 70.9, 70.5, 69.2, 68.9, 64.3, 45.9, 8.9; HR MS (ESI) *m/z*: calcd for C_28_H_26_O_4_N_6_Cl [M+H]^+^ 545.1699, found 545.1703.

*{3-[1-(4-Fluoro-phenyl)-1H-[1,2,3]triazol-4-yl]-phenyl}-(7,8,10,11,13,14-hexahydro-6,9,12,15-tetraoxa-1,3-diaza-cyclododeca[b]naphthalen-4-yl)-amine (* **a3***)*: White solid, Purity 98%; ^1^H NMR (600 MHz, DMSO-*d_6_*): *δ* 9.70 (s, 1H), 9.31 (s, 1H), 8.62 (s, 1H), 8.43 (s, 1H), 8.25 (s, 1H), 8.04 (d, *J_1_* = 8.4*_ Hz_*, *J_2_* = 4.7* Hz*, 2H), 7.96 (d, *J*=7.9 Hz, 1H), 7.66 (d, *J*=7.5 Hz, 1H), 7.63-7.39 (m, 3H), 7.35 (s, 1H), 4.32 (s, 4H), 3.80 (d, *J*=21.6 Hz, 4H), 3.65 (s, 4H); ^13^C NMR (150 MHz, DMSO-*d_6_*): 162.9, 161.4, 157.2, 156.5, 150.3, 147.8, 140.5, 133.7, 130.9, 129.6, 122.9, 122.8, 122.6, 121.1, 120.5, 119.4, 117.4, 117.2, 111.9, 110.8, 73.4, 70.9, 70.9, 70.5, 69.3, 68.9; HR MS (ESI) *m/z*: calcdfor C_28_H_26_O_4_N_6_F [M+H]^+^ 529.1994, found 529.2000.

*(7,8,10,11,13,14-Hexahydro-6,9,12,15-tetraoxa-1,3-diaza-cyclododeca[b]naphthalen-4-yl)-{3-[1-(2-methoxy-phenyl)-1H-[1,2,3]triazol-4-yl]-phenyl}-amine (**a4**)*: White solid, Purity 96%; ^1^H NMR (600 MHz, DMSO-*d_6_*): *δ* 9.63 (s, 1H), 8.92 (s, 1H), 8.53 (s, 1H), 8.41 (s, 1H), 8.25 (s, 1H), 7.97 (d, *J*=8.0 Hz, 1H), 7.70 (d, *J*=7.8 Hz, 1H), 7.66 (d, *J*=7.6 Hz, 1H), 7.57 (t, *J*=7.9 Hz, 1H), 7.50 (t, *J*=7.8 Hz, 1H), 7.36 (d, *J*=8.4 Hz, 1H), 7.32 (s, 1H), 7.19 (t, *J*=7.6 Hz, 1H), 4.32 (d, *J*=13.1 Hz, 4H), 3.90 (s, 3H), 3.80 (d, *J*=20.7 Hz, 4H), 3.65 (s, 4H); ^13^C NMR (150 MHz, DMSO-*d_6_*): 157.1, 156.4, 153.9, 152.3, 150.2, 148.1, 146.7, 140.6, 131.4, 131.2, 129.5, 126.4, 126.2, 123.9, 122.3, 121.4, 121.0, 119.2, 113.5, 112.1, 110.8, 110.3, 73.4, 70.9, 70.8, 70.5, 69.3, 68.9; HR MS (ESI) *m/z*: calcd for C_29_H_29_O_5_N_6_ [M+H]^+^ 541.2194, found 541.2197.

*{3-[1-(2-Chloro-phenyl)-1H-[1,2,3]triazol-4-yl]-phenyl}-(7,8,10,11,13,14-hexahydro-6,9,12,15-tetraoxa-1,3-diaza-cyclododeca[b]naphthalen-4-yl)-amine (* ***a5****)*: Yellow solid, Purity 98%; ^1^H NMR (600 MHz, DMSO-*d_6_*): *δ* 9.63 (s, 1H), 9.07 (s, 1H), 8.53 (s, 1H), 8.45 (s, 1H), 8.22 (s, 1H), 7.97 (d, *J*=8.1 Hz, 1H), 7.82 (t, *J*=9.0 Hz, 2H), 7.69-7.63 (m, 3H), 7.51 (t, *J*=7.9 Hz, 1H), 7.32 (s, 1H), 4.31 (d, *J*=14.9 Hz, 4H), 3.80 (d, *J*=21.4 Hz, 4H), 3.65 (s, 4H); ^13^C NMR (150 MHz, DMSO-*d_6_*): 157.1, 156.5, 153.9, 150.2, 148.2, 147.0, 140.6, 131.1, 130.9, 129.6, 129.1, 129.0, 128.9, 124.2, 119.3, 112.2, 110.7, 110.2, 73.4, 70.9, 70.8, 70.5, 69.3, 68.9; HR MS (ESI) *m/z*: calcd for C_28_H_26_O_4_N_6_Cl [M+H]^+^ 545.1699, found 545.1704.

*(7,8,10,11,13,14-Hexahydro-6,9,12,15-tetraoxa-1,3-diaza-cyclododeca[b]naphthalen-4-yl)-[3-(1-phenyl-1H-[1,2,3]triazol-4-yl)-phenyl]-amine (**a6**)*: Yellow solid, Purity 97.1%; ^1^H NMR (600 MHz, DMSO-*d_6_*): *δ* 9.68 (s, 1H), 9.33 (s, 1H), 8.53 (s, 1H), 8.45 (s, 1H), 8.24 (s, 1H), 7.99 (t, *J*=10.1 Hz, 3H), 7.67-7.64 (m, 3H), 7.53 (q, *J*=7.5 Hz, 2H), 7.32 (s, 1H), 4.32 (d, *J*=13.6 Hz, 4H), 3.79 (d, *J*=20.2 Hz, 4H), 3.65 (s, 4H); ^13^C NMR (150 MHz, DMSO-*d_6_*): 157.2, 156.4, 153.9, 150.2, 148.1, 147.8, 140.6, 137.1, 131.0, 130.4, 129.6, 129.2, 122.5, 121.0, 120.5, 120.2, 119.3, 112.2, 110.7, 110.3, 73.4, 70.9, 70.9, 70.5, 69.3, 68.9; HR MS (ESI) *m/z*: calcd for C_28_H_26_O_4_NaN_6_ [M+Na]^+^ 533.1913, found 533.1915.

*{3-[1-(3-Chloro-phenyl)-1H-[1,2,3]triazol-4-yl]-phenyl}-(7,8,10,11,13,14-hexahydro-6,9,12,15-tetraoxa-1,3-diaza-cyclododeca[b]naphthalen-4-yl)-amine (***a7***)*: Yellow solid, Purity 98%; ^1^H NMR (600 MHz, DMSO-*d_6_*): *δ* 9.70 (s, 1H), 9.41 (s, 1H), 8.68 (s, 1H), 8.44 (s, 1H), 8.28 (s, 1H), 8.13 (s, 1H), 7.99 (dd, *J_1_* = 27.4* Hz*, *J_2_* = 8.0* Hz*, 2H), 7.67 (dd, *J_1_* = 16.5* Hz*, *J_2_* = 8.0* Hz*, 2H), 7.60 (d, *J*=8.0 Hz, 1H), 7.53 (t, *J*=7.8 Hz, 1H), 7.37 (s, 1H), 4.32 (s, 4H), 3.79 (d, *J*=21.8 Hz, 4H), 3.65 (s, 4H); ^13^C NMR (150 MHz, DMSO-*d_6_*): 157.2, 156.5, 153.7, 150.3, 147.9, 140.5, 138.2, 134.7, 132.2, 130.8, 130.1, 129.7, 129.0, 122.6, 121.1, 120.4, 120.3, 119.4, 119.0, 112.0, 110.8, 93.3, 73.4, 70.9, 70.9, 70.5, 69.3, 68.9; HR MS (ESI) *m/z*: calcd for C_28_H_26_O_4_N_6_Cl [M+H]^+^ 545.1699, found 545.1705.

*(7,8,10,11,13,14-Hexahydro-6,9,12,15-tetraoxa-1,3-diaza-cyclododeca[b]naphthalen-4-yl)-{3-[1-(2-iodo-phenyl)-1H-[1,2,3]triazol-4-yl]-phenyl}-amine (**a8**)*: White solid, Purity 98%; ^1^H NMR (600 MHz, DMSO-*d_6_*): *δ* 9.65-9.60 (m, 1H), 8.99 (s, 1H), 8.58-8.46 (m, 2H), 8.32-8.14 (m, 2H), 7.96 (d, *J*=10.5 Hz, 1H), 7.68-7.65 (m, 2H), 7.57-7.40 (m, 2H), 7.32 (d, *J*=6.1 Hz, 1H), 5.70 (m, 1H), 4.31 (s, 4H), 3.79 (d, *J*=25.0 Hz, 4H), 3.65 (s, 4H); ^13^C NMR (150 MHz, DMSO-*d_6_*): 157.1, 156.5, 153.9, 150.3, 148.0, 147.0, 140.6, 140.3, 140.3, 140.0, 131.1, 130.2, 129.9, 129.6, 129.4, 128.6, 124.0, 122.3, 121.0, 119.2, 112.1, 110.8, 110.2, 96.5, 73.4, 70.9, 70.8, 70.5, 69.3, 68.9, 58.0; HR MS (ESI) *m/z*: calcd for C_28_H_26_O_4_N_6_I [M+H]^+^ 637.1060, found 637.1065.

*(7,8,10,11,13,14-Hexahydro-6,9,12,15-tetraoxa-1,3-diaza-cyclododeca[b]naphthalen-4-yl)-{3-[1-(3-methoxy-phenyl)-1H-[1,2,3]triazol-4-yl]-phenyl}-amine (**a9**)*: Brown solid, Purity 97%; ^1^H NMR (600 MHz, DMSO-*d_6_*): *δ* 9.69 (s, 1H), 9.39 (s, 1H), 8.58 (s, 1H), 8.47 (s, 1H), 8.27 (s, 1H), 8.02 (d, *J*=7.9 Hz, 1H), 7.70 (d, *J*=7.6 Hz, 1H), 7.63-7.56 (m, 4H), 7.37 (s, 1H), 7.14 (d, *J*=7.9 Hz, 1H), 4.36 (d, *J*=11.7 Hz, 4H), 3.94 (s, 3H), 3.84 (d, *J*=20.1 Hz, 4H), 3.70 (s, 4H); ^13^C NMR (150 MHz, DMSO-*d_6_*): 160.7, 157.1, 156.5, 153.9, 150.3, 148.1, 147.8, 140.6, 138.2, 131.4, 131.0, 129.6, 122.5, 121.0, 120.2, 119.3, 114.9, 112.4, 112.2, 110.7, 110.2, 106.1, 73.4, 70.9, 70.9, 70.5, 69.3, 68.9, 56.1; HR MS (ESI) *m/z*: calcd for C_29_H_28_O_5_N_6_Na [M+Na]^+^ 563.2013, found 563.2017.

*{3-[1-(4-Bromo-phenyl)-1H-[1,2,3]triazol-4-yl]-phenyl}-(7,8,10,11,13,14-hexahydro-6,9,12,15-tetraoxa-1,3-diaza-cyclododeca[b]naphthalen-4-yl)-amine (**a10**)*: White solid, Purity 98%; ^1^H NMR (600 MHz, DMSO-*d_6_*): *δ* 9.63 (s, 1H), 9.36 (s, 1H), 8.53 (s, 1H), 8.44 (s, 1H), 8.22 (s, 1H), 7.97 (d, *J*=8.6 Hz, 3H), 7.86 (d, *J*=8.6 Hz, 2H), 7.65 (d, *J*=7.6 Hz, 1H), 7.52 (t, *J*=7.9 Hz, 1H), 7.32 (s, 1H), 4.32 (d, *J*=11.2 Hz, 4H), 3.79 (d, *J*=20.5 Hz, 4H), 3.65 (s, 4H); ^13^C NMR (150 MHz, DMSO-*d_6_*): 157.1, 156.5, 153.9, 150.3, 148.1, 148.0, 140.6, 136.3, 133.3, 130.8, 129.6, 122.5, 122.4, 121.8, 121.0, 120.2, 119.3, 112.2, 110.7, 110.2, 73.4, 70.9, 70.9, 70.5, 69.3, 68.9; HR MS (ESI) *m/z*: calcd for C_28_H_25_O_4_N_6_BrNa [M+Na]^+^ 611.1013, found 611.1020.

*{3-[1-(2-Bromo-phenyl)-1H-[1,2,3]triazol-4-yl]-phenyl}-(7,8,10,11,13,14-hexahydro-6,9,12,15-tetraoxa-1,3-diaza-cyclododeca[b]naphthalen-4-yl)-amine (**a11**)*: Yellow solid, Purity 97%; ^1^H NMR (600 MHz, DMSO-*d_6_*): *δ* 9.64 (s, 1H), 9.04 (s, 1H), 8.54 (s, 1H), 8.45 (s, 1H), 8.22 (s, 1H), 7.97 (d, *J*=8.0 Hz, 2H), 7.77 (d, *J*=7.8 Hz, 1H), 7.71-7.65 (m, 2H), 7.60 (dd, *J_1_* = 17.2* Hz*, *J_2_* = 9.5* Hz*, 1H), 7.51 (t, *J*=7.8 Hz, 1H), 7.32 (s, 1H), 4.32 (d, *J*=10.4 Hz, 4H), 3.79 (d, *J*=21.8 Hz, 4H), 3.65 (s, 4H); ^13^C NMR (150 MHz, DMSO-*d_6_*): *d* 157.1, 156.5, 153.9, 150.2, 148.1, 146.9, 140.6, 136.7, 134.1, 132.6, 130.9, 129.6, 129.5, 129.2, 124.2, 122.4, 121.0, 119.5, 119.3, 112.1, 110.8, 110.2, 73.4, 70.9, 70.8, 70.5, 69.3, 68.9; HR MS (ESI) *m/z*: calcd for C_28_H_25_O_4_N_6_BrNa [M+Na]^+^ 611.1013, found 611.1021.

*(7,8,10,11,13,14-Hexahydro-6,9,12,15-tetraoxa-1,3-diaza-cyclododeca[b]naphthalen-4-yl)-{3-[1-(4-trifluoromethyl-phenyl)-1H-[1,2,3]triazol-4-yl]-phenyl}-amine (**a12**)*: White solid, Purity 99%; ^1^H NMR (600 MHz, DMSO-*d_6_*): *δ* 9.69 (s, 1H), 9.48 (s, 1H), 8.45 (s, 1H), 8.36 (s, 1H), 8.25 (d, *J*=8.0 Hz, 2H), 8.04 (d, *J*=8.0 Hz, 2H), 7.97 (d, *J*=7.5 Hz, 1H), 7.68 (d, *J*=7.4 Hz, 1H), 7.53 (t, *J*=7.6 Hz, 1H), 4.32 (s, 4 H), 3.79 (d, *J*=19.9 Hz, 4H), 3.64 (s, 4H); ^13^C NMR (150 MHz, DMSO-*d_6_*): 156.7, 156.3, 150.4, 148.1, 140.7, 139.9, 130.7, 129.9, 129.3, 129.1, 128.8, 127.8, 125.2, 123.4, 122.6, 121.6, 121.1, 120.9, 120.4, 119.4, 110.9, 73.4, 70.9, 70.9, 70.5, 69.3, 68.9; HR MS (ESI) *m/z*: calcd for C_29_H_26_O_4_N_6_F_3_ [M+H]^+^ 579.1962, found 579.1972.

*{3-[1-(2-Fluoro-benzyl)-1H-[1,2,3]triazol-4-yl]-phenyl}-(7,8,10,11,13,14-hexahydro-6,9,12,15-tetraoxa-1,3-diaza-cyclododeca[b]naphthalen-4-yl)-amine (**a13**)*: Yellow solid, Purity 98%; ^1^H NMR (600 MHz, DMSO-*d_6_*): *δ* 9.99 (s, 1H), 9.05 (s, 1H), 8.93 (s, 1H), 8.73 (t, *J*=1.8 Hz, 1H), 8.61 (s, 1H), 8.36 (d, *J*=7.3 Hz, 1H), 7.99 (d, *J*=7.7 Hz, 1H), 7.89-7.82 (m, 3H), 7.73-7.66 (m, 3H), 6.16 (s, 2H), 4.73 (d, *J*=16.9 Hz, 4H), 4.23-4.18 (m, 4H), 4.07 (s, 4H); ^13^C NMR (150 MHz, DMSO-*d_6_*): 161.8, 160.2, 157.5, 156.9, 154.3, 150.6, 148.5, 147.5, 140.9, 131.7, 131.7, 129.9, 125.8, 123.7, 122.6, 122.5, 121.2, 119.5, 116.6, 116.5, 112.6, 111.1, 110.6, 73.8, 71.4, 71.3, 70.9, 69.7, 69.3, 48.0; HRMS (ESI) *m/z*: calcd for C_29_H_28_O_4_N_6_F [M+H]^+^ 543.2151, found 543.2158.

*(7,8,10,11,13,14-Hexahydro-6,9,12,15-tetraoxa-1,3-diaza-cyclododeca[b]naphthalen-4-yl)-{3-[1-(2-methyl-benzyl)-1H-[1,2,3]triazol-4-yl]-phenyl}-amine (**a14**)*: Yellow solid, Purity 98%; ^1^H NMR (600 MHz, DMSO-*d_6_*): *δ* 9.61 (s, 1H), 8.70 (s, 1H), 8.56 (s, 1H), 8.31 (s, 1H), 8.25 (s, 1H), 7.93 (d, *J*=8.0 Hz, 1H), 7.57 (d, *J*=7.6 Hz, 1H), 7.45 (t, *J*=7.9 Hz, 1H), 7.37 (s, 1H), 7.28-7.21 (m, 3H), 7.16 (d, *J*=7.5 Hz, 1H), 5.68 (s, 2H), 4.31 (s, 4H), 3.78 (d, *J*=24.3 Hz, 4H), 3.64 (s, 4H), 2.36 (s, 3H); ^13^C NMR (150 MHz, DMSO-*d_6_*): 157.1, 156.4, 153.7, 150.3, 147.0, 140.5, 136.8, 134.6, 131.4, 130.9, 129.5, 129.2, 128.8, 126.8, 122.1, 120.9, 119.1, 112.3, 110.8, 73.4, 70.9, 70.9, 70.5, 69.3, 68.9, 51.7, 19.2; HRMS (ESI) *m/z*: calcd for C_30_H_31_O_4_N_6_ [M+H]^+^539.2401, found 539.2406.

*(7,8,10,11,13,14-Hexahydro-6,9,12,15-tetraoxa-1,3-diaza-cyclododeca[b]naphthalen-4-yl)-{3-[1-(4-methyl-benzyl)-1H-[1,2,3]triazol-4-yl]-phenyl}-amine (**a15**)*: Brown solid, Purity 98%; ^1^H NMR (600 MHz, DMSO-*d_6_*): *δ* 9.59 (s, 1H), 8.61 (s, 2H), 8.30 (s, 1H), 8.24 (s, 1H), 7.93 (d, *J*=8.0 Hz, 1H), 7.55 (d, *J*=7.6 Hz, 1H), 7.45 (t, *J*=7.9 Hz, 1H), 7.35 (s, 1H), 7.28 (d, *J*=7.7 Hz, 2H), 7.21 (d, *J*=7.7 Hz, 2H), 5.61 (s, 2H), 4.31 (s, 4H), 3.79 (d, J=23.8 Hz, 4H), 3.65 (s, 4H), 2.30 (s, 3H); ^13^C NMR (150 MHz, DMSO-*d_6_*): 157.1, 156.5, 153.7, 150.3, 147.1, 140.5, 138.0, 133.5, 131.4, 129.8, 129.5, 128.5, 122.1, 121.9, 120.8, 119.1, 112.3, 110.8, 73.4, 70.9, 70.9, 70.5, 69.3, 68.9, 53.3, 21.2; HRMS (ESI) *m/z*: calcd for C_30_H_31_O_4_N_6_ [M+H]^+^ 539.2401, found 539.2408.

*(7,8,10,11,13,14-Hexahydro-6,9,12,15-tetraoxa-1,3-diaza-cyclododeca[b]naphthalen-4-yl)-{3-[1-(2-iodo-benzyl)-1H-[1,2,3]triazol-4-yl]-phenyl}-amine (**a16**)*: Yellow solid, Purity 96%; ^1^H NMR (600 MHz, DMSO-*d_6_*): *δ* 9.61 (s, 1H), 8.57 (d, *J*=16.0 Hz, 2H), 8.32 (s, 1H), 8.21 (s, 1H), 7.95 (dd, *J_1_* = 13.5 Hz , *J_2_ =* 8.0 Hz , 2H), 7.58 (d, *J*=7.6 Hz, 1H), 7.45 (dd, *J_1_* = 14.9 Hz , *J_2_* = 7.5 Hz , 2H), 7.32 (s, 1H), 7.17-7.13 (m, 2H), 5.70 (s, 2H), 4.31 (s, 4H), 3.79 (d, *J*=23.6 Hz, 4H), 3.65 (s, 4H); ^13^C NMR (150 MHz, DMSO-*d_6_*): 157.1, 156.5, 153.7, 150.2, 147.9, 147.0, 140.5, 140.0, 138.4, 131.3, 130.8, 130.2, 129.5, 129.4, 122.5, 122.1, 120.9, 119.1, 112.0, 110.8, 110.3, 99.7, 73.4, 70.9, 70.8, 70.5, 69.3, 68.9, 58.0; HRMS (ESI) *m/z*: calcd for C_29_H_28_O_4_N_6_I [M+H]^+^ 651.1211, found 651.1220.

*{3-[1-(3-Bromo-benzyl)-1H-[1,2,3]triazol-4-yl]-phenyl}-(7,8,10,11,13,14-hexahydro-6,9,12,15-tetraoxa-1,3-diaza-cyclododeca[b]naphthalen-4-yl)-amine (**a17**)*: White solid, Purity 98%; ^1^H NMR (600 MHz, DMSO-*d_6_*): *δ* 9.59 (s, 1H), 8.68 (s, 1H), 8.53 (s, 1H), 8.32 (s, 1H), 8.20 (s, 1H), 7.93 (d, *J*=8.0 Hz, 1H), 7.62 (s, 1H), 7.56 (d, *J*=6.9 Hz, 2H), 7.46 (t, *J*=7.9 Hz, 1H), 7.38 (d, *J*=4.4 Hz, 2H), 7.31 (s, 1H), 5.69 (s, 2H), 4.31 (s, 4H), 3.79 (d, *J*=22.7 Hz, 4H), 3.65 (s, 4H); ^13^C NMR (150 MHz, DMSO-*d_6_*): 157.1, 156.5, 153.8, 150.3, 148.0, 147.2, 140.5, 139.1, 131.6, 131.5, 131.2, 129.5, 127.6, 122.4, 122.3, 122.1, 120.8, 119.1, 112.1, 110.7, 110.3, 73.4, 70.9, 70.8, 70.5, 69.3, 68.9, 52.7; HRMS (ESI) *m/z*: calcd for C_29_H_28_O_4_N_6_Br [M+H]^+^ 603.1350, found 603.1356.

*[3-(1-Benzyl-1H-[1,2,3]triazol-4-yl)-phenyl]-(7,8,10,11,13,14-hexahydro-6,9,12,15-tetraoxa-1,3-diaza-cyclododeca[b]naphthalen-4-yl)-amine (**a18**)*: Brown solid, Purity 95%; ^1^H NMR (600 MHz, DMSO-*d_6_*): *δ* 9.60 (s, 1H), 8.66 (s, 1H), 8.51 (s, 1H), 8.32 (s, 1H), 8.21 (s, 1H), 7.93 (d, *J*=7.9 Hz, 1H), 7.56 (d, *J*=7.6 Hz, 1H), 7.47-7.35 (m, 6H), 7.31 (s, 1H), 5.67 (s, 2H), 4.31 (s, 4H), 3.81-3.76 (m, 4H), 3.65 (s, 4H); ^13^C NMR (150 MHz, DMSO-*d_6_*): 157.1, 156.4, 153.9, 150.2, 148.1, 147.1, 140.5, 136.5, 131.4, 129.5, 129.3, 128.7, 128.4, 122.1, 122.1, 120.8, 119.1, 112.1, 110.7, 73.4, 70.9, 70.9, 70.5, 69.3, 68.9, 53.5; HR MS (ESI) *m/z*: calcd for C_29_H_29_O_4_N_6_ [M+H]^+^ 525.2245, found 525.2254.

*{3-[1-(2-Bromo-benzyl)-1H-[1,2,3]triazol-4-yl]-phenyl}-(7,8,10,11,13,14-hexahydro-6,9,12,15-tetraoxa-1,3-diaza-cyclododeca[b]naphthalen-4-yl)-amine (**a19**)*:White solid, Purity 98%; ^1^H NMR (600 MHz, DMSO-*d_6_*): *δ* 9.59 (s, 1H), 8.62 (s, 1H), 8.51 (s, 1H), 8.32 (s, 1H), 8.20 (s, 1H), 7.94 (d, *J*=8.3 Hz, 1H), 7.73 (d, *J*=7.8 Hz, 1H), 7.57 (d, *J*=7.5 Hz, 1H), 7.45 (dd, *J_1_* = 14.4* Hz*, *J_2_* = 7.4* Hz*, 2H), 7.35 (t, *J*=7.7 Hz, 1H), 7.31 (s, 1H), 7.26 (d, *J*=7.7 Hz, 1H), 5.76 (s, 2H), 4.31 (s, 4H), 3.79 (d, *J*=23.7 Hz, 4H), 3.63 (s, 4H); HR MS (ESI) *m/z*: calcd for C_29_H_27_O_4_N_6_BrNa [M+Na]^+^ 625.1169, found 625.1178.

*(7,8,10,11,13,14-Hexahydro-6,9,12,15-tetraoxa-1,3-diaza-cyclododeca[b]naphthalen-4-yl)-{3-[1-(3-methoxy-benzyl)-1H-[1,2,3]triazol-4-yl]-phenyl}-amine (**a20**)*: Yellow solid, Purity 97%; ^1^H NMR (600 MHz, DMSO-*d_6_*): *δ* 9.72 (s, 1H), 8.66 (s, 1H), 8.50 (s, 1H), 8.35 (s, 1H), 8.31 (s, 1H), 7.95 (d, *J*=8.0 Hz, 1H), 7.55 (d, *J*=7.5 Hz, 1H), 7.44 (t, *J*=7.9 Hz, 1H), 7.35-7.28 (m, 2H), 6.98 (s, 1H), 6.93 (d, *J*=7.9 Hz, 2H), 5.63 (s, 2H), 4.32 (d, *J*=23.7 Hz, 4H), 3.80 (s, 2H), 3.76 (s, 5H), 3.64 (s, 4H); ^13^C NMR (150 MHz, DMSO-*d_6_*): 159.9, 157.1, 156.4, 153.9, 150.2, 148.1, 147.1, 140.6, 137.9, 131.3, 130.5, 129.4, 122.1, 120.7, 120.5, 119.2, 114.3, 114.0, 112.2, 110.8, 110.3, 73.3, 70.9, 70.4, 69.2, 68.8, 55.6, 53.4; HR MS (ESI) *m/z*: calcd for C_30_H_30_O_5_N_6_Na [M+Na]^+^ 577.2170, found 577.2176.

*{3-[1-(3,5-Dibromo-benzyl)-1H-[1,2,3]triazol-4-yl]-phenyl}-(7,8,10,11,13,14-hexahydro-6,9,12,15-tetraoxa-1,3-diaza-cyclododeca[b]naphthalen-4-yl)-amine (**a21**)*: Yellow solid, Purity 97%; ^1^H NMR (600 MHz, DMSO-*d_6_*): *δ* 9.62 (s, 1H), 8.71 (s, 1H), 8.51 (s, 1H), 8.33 (s, 1H), 8.22 (s, 1H), 7.93 (d, *J*=8.2 Hz, 1H), 7.86 (s, 1H), 7.64 (s, 2H), 7.57 (d, *J*=7.5 Hz, 1H), 7.46 (t, *J*=7.8 Hz, 1H), 7.31 (s, 1H), 5.70 (s, 2H), 4.32 (s, 4H), 3.78 (d, *J*=26.7 Hz, 4H), 3.65 (s, 4H); ^13^C NMR (150 MHz, DMSO-*d_6_*): 157.1, 156.4, 153.9, 150.2, 148.1, 147.2, 140.8, 140.5, 133.7, 131.2, 130.7, 129.5, 123.2, 122.4, 122.2, 120.8, 119.1, 112.2, 110.7, 110.2, 73.4, 70.9, 70.9, 70.5, 69.2, 68.9, 52.0; HR MS (ESI) *m/z*: calcd for C_29_H_26_O_4_N_6_Br_2_Na [M+Na]^+^703.0274, found 703.0281.

*(3-{1-[2-(4-Fluoro-phenyl)-ethyl]-1H-[1,2,3]triazol-4-yl}-phenyl)-(7,8,10,11,13,14-hexahydro-6,9,12,15-tetraoxa-1,3-diaza-cyclododeca[b]naphthalen-4-yl)-amine (**a22**)*: Brown solid, Purity 95%; ^1^H NMR (600 MHz, DMSO-*d_6_*): *δ* 9.58 (s, 1H), 8.52 (s, 2H), 8.30 (s, 1H), 8.20 (s, 1H), 7.92 (d, *J*=7.9 Hz, 1H), 7.51 (d, *J*=7.5 Hz, 1H), 7.45 (t, *J*=7.8 Hz, 1H), 7.32 (s, 1H), 7.29-7.16 (m, 2H), 7.12 (t, *J*=8.7 Hz, 2H), 4.67 (t, *J*=7.2 Hz, 2H), 4.31 (s, 4H), 3.79 (d, *J*=21.0 Hz, 4H), 3.65 (s, 4H), 3.24 (t, *J*=7.2 Hz, 2H); ^13^C NMR (150 MHz, DMSO-*d_6_*): 162.4, 160.7, 157.1, 156.4, 153.9, 150.2, 148.1, 148.1, 146.6, 140.5, 134.3, 131.5, 131.1, 131.0, 121.9, 121.9, 120.7, 119.0, 115.7, 115.6, 112.2, 110.7, 110.2, 73.4, 70.9, 70.9, 70.5, 69.3, 68.9, 51.1, 35.2; HR MS (ESI) *m/z*: calcd for C_30_H_29_O_4_N_6_FNa [M+Na]^+^ 579.2127, found 579.2134.

#### IDO1 Enzymatic Inhibition Assay

To perform the Hela cell based IDO1 assay, Hela cells were seeded at 50,000 cells per well into 96-well microplate in 100 μl of DMEM 10% fetal bovine serum 1% Penicillin-Streptomycin. Cells were incubated at 37°C and 5% CO_2_ overnight.

The next day 100 μl per well of diluted inhibitor in growth medium was added at a final concentration of 100 ng/ml human IFN-γ. Cells were incubated at 37°C in a CO_2_ incubator for 18 h. The next day 140 μl of medium was moved into a new 96-well plate and 20 μl of 3.05 N trichloroacetic acid (TCA) was added. The plate was incubated at 50°C for 30 min to hydrolyze N-formylkynurenine. The plate was then centrifuged at 2,500 rpm for 10 min to remove sediments. 100 μl of supernatant per well was transferred to another 96-well plate and mixed with 100 μl of 2% (w/v) 4-(Dimethylamino)benzaldehyde in acetic acid. The plate was incubated at room temperature for 10 min, the yellow color derived from kynurenine was recorded by measuring absorbance at 480 nm using a microplate reader (PerkinElmer, USA).

#### Molecular Modeling

Molecular docking studies were performed with the *Glide*6.6 module in Schrödinger 2015, and the IDO1 complex with Amg-1 (PDB:4pk5) was used. The Protein preparation module in Maestro 10.1 was used to assign bond orders, add hydrogens, create zero-order bond to metals, create disulfide bonds, delete water molecules beyond 5 Å from het group, assign partial charge, assign protonation states, and minimize the structure with OPLS-2005 force field. The *Ligprep*3.3 module in Maestro 10.1 was used to generate stereoisomers, and the protonation states of ligands at pH 7.0 ± 2.0 were generated with *Epik*3.1. For the other parameters. the molecular interactions between ligand and receptor were visualized with Pymol software.

#### Cytotoxicity Assay

Cytotoxity of the chosen compounds was evaluated by the Cell Counting Kit-8 (CCK8, DOJINDO, Japan) assay. The cells were seeded at a density of 2,000 cells per well into 96-well microplate in 100 μl of growth medium. Cells were incubated at 37°C and 5% CO_2_ overnight. The next day, 100 μl per well of diluted inhibitor in growth medium was added with the final concentration from 0.1nM to 100 μM. The cells were treated with DMSO as control. A series of dilutions were made in 0.1% DMSO in assay medium so that the final concentration of DMSO was 0.1% in all the treatments. Cells were incubated at 37°C and 5% CO_2_ for 72 h. Then, 10 μl of CCK8 was added to each well. The plates were incubated at 37°C for 2 h, and the plates were recorded by measuring the absorbance at 450 nm with the reference wavelength of 630 nm using an EnVisionMultilabel Reader (PerkinElmer). The IC_50_ values were calculated and determined by the concentration causing a half-maximal percent activity. All assays were conducted with three parallel samples and three repetitions.

## Results and Discussion

### IDO1 Inhibition Study

To investigate the IDO1 inhibition activities of the synthesized derivatives, all the new compounds and icotinib were screened *via* Hela cell-based functional assay using methods described in the literature (Yue et al., 2009; Malachowski et al., 2016; Qian et al., 2016). BMS-986205 was also used as a positive control and the IC_50_ value was tested as 0.62 nM, which is consistent with the results previously reported by Nelp et al. (IC_50 =_ 0.5 nM) (Nelp et al., 2018).

As demonstrated in **Table 1**, IDO1 inhibitory activity showed that several compounds exhibited higher IDO1 inhibitory activity than icotinib, such as **a4** (IC_50_ = 1.32μM), **a6** (IC_50_ = 0.77μM), **a8** (IC_50_ = 2.50μM), **a9** (IC_50_ = 1.41μM), **a11** (IC_50_ = 1.00μM), **a14** (IC_50_ = 0.79μM), **a15** (IC_50_ = 0.59μM), **a16**(IC_50_ = 1.51μM), **a17** (IC_50_ = 0.37μM), **a18** (IC_50_ = 0.56μM), **a19** (IC_50_ = 1.50μM), **a20** (IC_50_ = 0.76μM), **a21** (IC_50_ = 0.68μM), and **a22** (IC_50_ = 0.81μM), suggesting that the inhibitory activity of the compounds against IDO1 could be enhanced upon introduction of triazolegroups, and the triazole ring might be used as an active group to interact with the IDO1. Carefully examining the results also suggested that when the benzene ring bore the same substituent, the compounds with benzyl-linked triazole groups were generally more active than those with phenyl-linked triazoles especially for those showing submicromolar levels of IC_50_ values against IDO1.

**Table 1:** IDO1 inhibitory activities of compounds **a1–a22**.

Compd no.	n	R^1^	R^2^	R^3^	R^4^	IC_50_ (μM)
						IDO1
**a1**	0	H	F	H	H	4.25 ± 0.08
**a2**	0	H	H	Cl	H	2.90 ± 0.37
**a3**	0	H	H	F	H	18.19 ± 1.47
**a4**	0	OCH_3_	H	H	H	1.32 ± 0.17
**a5**	0	Cl	H	H	H	4.88 ± 0.42
**a6**	0	H	H	H	H	0.77 ± 0.13
**a7**	0	H	Cl	H	H	2.82 ± 0.26
**a8**	0	I	H	H	H	2.50 ± 0.43
**a9**	0	H	OCH_3_	H	H	1.41 ± 0.08
**a10**	0	H	H	Br	H	2.79 ± 0.46
**a11**	0	Br	H	H	H	1.00 ± 0.49
**a12**	0	H	H	CF_3_	H	>100
**a13**	1	F	H	H	H	4.62 ± 0.19
**a14**	1	CH_3_	H	H	H	0.79 ± 0.21
**a15**	1	H	H	CH_3_	H	0.59 ± 0.05
**a16**	1	I	H	H	H	1.51 ± 0.11
**a17**	1	H	Br	H	H	0.37 ± 0.02
**a18**	1	H	H	H	H	0.56 ± 0.16
**a19**	1	Br	H	H	H	1.50 ± 0.45
**a20**	1	H	OCH_3_	H	H	0.76 ± 0.07
**a21**	1	H	Br	H	Br	0.68 ± 0.44
**a22**	2	H	H	F	H	0.81 ± 0.35
**icotinib**						2.57 ± 0.44

### Molecular Docking Studies

Docking experiments were then carried out to explore the potential binding mode between the prepared compounds and IDO1. Compounds **a17** and compound **a18**, which showed the best activity against IDO1, were chosen as model compounds for the experiments. The docking results are depicted in **Figure 3**. The molecular docking results suggested that **a17** and **a18** could be docked into the hydrophobic site of IDO1with docking score of -8.41 and -8.19 kcal/mol, respectively. The docking experiments also suggested that compound **a17** and **a18** could bind to the binding pocket, the triazole structure mainly located on the top of the HEM, and one nitrogen atom tended to form a coordination bond with the iron in the heme. The triazole ring of a17 formed a π-π interaction with the benzene ring of phenylalanine 163. The benzyl group at the N3 position of 1,2,3-triazole could occupied the hydrophobic pocket containing cysteine 129 above the heme, the backbone amino group of glycine 261 formed a hydrogen bond with one oxygen of the hydroxyl group, and the main chain amino group of glycine 236 formed hydrogen bonds with the benzene ring. For compound **a18**, no intermolecular hydrogen bonds are formed except for the formation of coordination bonds. These results were generally in good agreement with previous study that compounds containing coordinating atoms would act as potential inhibitors of IDO1 (Röhrig et al., 2012; Tojo et al., 2014).

Compounds **a3** and **a12** should poor biological activity. These two compounds contained phenyl groups with strong electron withdrawing groups in the para position. Preliminary docking experiments in **Figure 4** suggested that due to the lack of methylene group, insertion of the phenyl groups into the hydrophobic pocket consisting L234 and Y126 residues was difficult. In addition, the distance between the triazolegroup and the HEM ion is 4.7Å and 6.5Å, respectively, which is also consistent with the low activity of **a3** and **a12**.

### Cytotoxicity Study

Next, several compounds with submicromolar level of IDO1 inhibitory activities were chosen for further study. The results are given in **Table 2**. Human renal epithelial cell (293T) was chosen for CCK-8 assay to evaluation the bioactivity of these compounds. As shown in **Table 2**, human renal epithelial cell 293T showed poor sensitivity to **a6** with IC_50_ values of 42.79 ± 11.81 μM, and icotinib-triazole derivatives such as **a15** and **a21** exhibited stronger killing effects on the cell line with IC_50_ values of 0.16 ± 0.05 μM and 0.74 ± 0.15 μM, respectively. Among these compounds, **a17** and **a18** showed medium cytotoxic effects on the 293T cell line, and the IC_50_ values of **a7** and **a18** on the cell line were 3.10 ± 0.20 μM and 3.08 ± 0.59 μM, respectively.

**Table 2:** Cytotoxicity of selected compounds.

Compd no.	IC_50_ (μM)	
	IDO1	293T
**a6**	0.77 ± 0.13	42.79 ± 11.81
**a14**	0.79 ± 0.21	3.35 ± 0.89
**a15**	0.59 ± 0.05	0.16 ± 0.05
**a17**	0.37 ± 0.02	3.10 ± 0.20
**a18**	0.56 ± 0.16	3.08 ± 0.59
**a20**	0.76 ± 0.07	3.30 ± 1.30
**a21**	0.68 ± 0.44	0.74 ± 0.15
**a22**	0.81 ± 0.35	2.60 ± 0.17

## Conclusion

In summary, a series of icotinib derivatives containing 1,2,3-triazole ringsprepared and evaluated for the inhibition of IDO1. Most of the compounds exhibited better IDO1 inhibitory activities than the parent icotinib. For example, submicromolar levels of IC_50_ were observed for compound **a17** and **a18**, with the IC_50_ value of 0.37 μM and 0.56 μM, respectively. Docking experiments suggest that icotinib-1,2,3-triazole derivatives are potential IDO1 inhibitors that preferentially bind to the ferrous form of IDO1 by forming coordinate bond with the haem iron. However, considering the fact that several candidates are currently undergoing clinical trials but none of these has been approved so far, the identification of potent and clinically useful IDO1 inhibitors is still an open challenge. In addition, some toxicity problem arose when triazole functionality was introduced to icotinib, suggesting that one should be very careful when introducing additional pharmacophores into a known drug especially when the mode of interaction was altered after the introduction of the additional functional group. Ideally, the designed compounds should show significant toxicity against the cancer cell on one hand, and good safety against normal cells on the other. We are now designing new structures using the scaffold hopping strategy, and the results will be presented in due time.

## Data Availability Statement

The raw data supporting the conclusions of this article will be made available by the authors, without undue reservation.

## Author Contributions

All authors contributed to the article and approved the submitted version.

## Funding

This study was supported by the Scientific and Technological Project of Henan Province (No. 192102310142), the Tianjin Research Innovation Project for Postgraduate Students (No. 2019YJSB077), the Shaanxi University of Chinese Medicine (No. 2020XG01), and the Subject Innovation Team of Shaanxi University of Chinese Medicine (No. 2019-PY02). Y-ML acknowledged the financial support from the National Natural Science Foundation of China (NSFC 21672106).

## Conflict of Interest

The authors declare that the research was conducted in the absence of any commercial or financial relationships that could be construed as a potential conflict of interest.

## Supplementary Material

The Supplementary Material for this article can be found online at: https://www.frontiersin.org/articles/10.3389/fphar.2020.579024/full#supplementary-material.
