# Supplementary material for: Discovery of Icotinib-1,2,3-Triazole Derivatives as IDO1 Inhibitors
Source: Front Pharmacol. 2020 Sep 30;11:579024. doi: 10.3389/fphar.2020.579024 (PMC7555427; doi:10.3389/fphar.2020.579024)

Supplementary Information

Discovery of icotinib-1,2,3-triazole derivatives as IDO1 inhibitors

Long-fei Mao[1,2†], Yu-wei Wang[3†], Jie Zhao[2], Gui-qing Xu[2], Xiao-jun Yao[4*] and Yue-Ming Li[1*]

[1] State Key Laboratory of Medicinal Chemical Biology, College of Pharmacy and Tianjin Key Laboratory of Molecular Drug Research, Nankai University, Tianjin, China

[2] School of Chemistry and Chemical Engineering, Henan Engineering Research Center of Chiral Hydroxyl Pharmaceutical, Henan Normal University, Xinxiang, China,

[3] College of Pharmacy, Shaanxi University of Chinese Medicine, Xi'an-Xianyang New Economic Zone, China

[4] State Key Laboratory of Quality Research in Chinese Medicine/Macau Institute for Applied Research in Medicine and Health, Macau University of Science and Technology, Macau (SAR), China

Table of Contents

Figure S1-1. ^{1}H NMR spectrum (600MHz, DMSO-d6) of compound a1 1
Figure S1-2. ^{13}C NMR spectrum (150MHz, DMSO-d6) of compound a1 2
Figure S1-3. HR MS of compound a1 3
Figure S2-1. ^{1}H NMR spectrum (600MHz, DMSO-d6) of compound a2 4
Figure S2-2. HR MS of compound a2 5
Figure S3-1. ^{1}H NMR spectrum (600MHz, DMSO-d6) of compound a3 6
Figure S3-2. ^{13}C NMR spectrum (150MHz, DMSO-d6) of compound a3 7
Figure S3-3. HR MS of compound a3 8
Figure S4-1. ^{1}H NMR spectrum (600MHz, DMSO-d6) of compound a4 9
Figure S4-2. ^{13}C NMR spectrum (150MHz, DMSO-d6) of compound a4 10
Figure S4-3. HR MS of compound a4 11
Figure S5-1. ^{1}H NMR spectrum (600MHz, DMSO-d6) of compound a5 12
Figure S5-2. ^{13}C NMR spectrum (150MHz, DMSO-d6) of compound a5 13
Figure S5-3. HR MS of compound a5 14
Figure S6-1. ^{1}H NMR spectrum (600MHz, DMSO-d6) of compound a6 15
Figure S6-2. ^{13}C NMR spectrum (150MHz, DMSO-d6) of compound a6 16
Figure S6-3. HR MS of compound a6 17
Figure S7-1. ^{1}H NMR spectrum (600MHz, DMSO-d6) of compound a7 18
Figure S7-2. ^{13}C NMR spectrum (150MHz, DMSO-d6) of compound a7 19
Figure S7-3. HR MS of compound a7 20
Figure S8-1. ^{1}H NMR spectrum (600MHz, DMSO-d6) of compound a8 21
Figure S8-2. ^{13}C NMR spectrum (150MHz, DMSO-d6) of compound a8 22
Figure S8-3. HR MS of compound a8 23
Figure S9-1. ^{1}H NMR spectrum (600MHz, DMSO-d6) of compound a9 24
Figure S9-2. ^{13}C NMR spectrum (150MHz, DMSO-d6) of compound a9 25
Figure S9-3. HR MS of compound a9 26
Figure S10-1. ^{1}H NMR spectrum (600MHz, DMSO-d6) of compound a10 27
Figure S10-2. ^{13}C NMR spectrum (150MHz, DMSO-d6) of compound a10 28
Figure S10-3. HR MS of compound a10 29
Figure S11-1. ^{1}H NMR spectrum (600MHz, DMSO-d6) of compound a11 30
Figure S11-2. ^{13}C NMR spectrum (150MHz, DMSO-d6) of compound a11 31
Figure S11-3. HR MS of compound a11 32
Figure S12-1. ^{1}H NMR spectrum (600MHz, DMSO-d6) of compound a12 33
Figure S12-2. ^{12}C NMR spectrum (150MHz, DMSO-d6) of compound a12 34
Figure S12-3. HR MS of compound a12 35
Figure S13-1. ^{1}H NMR spectrum (600MHz, DMSO-d6) of compound a13 36
Figure S13-2. ^{13}C NMR spectrum (150MHz, DMSO-d6) of compound a13 37
Figure S13-3. HR MS of compound a13 38
Figure S14-1. ^{1}H NMR spectrum (600MHz, DMSO-d6) of compound a14 39
Figure S14-2. ^{13}C NMR spectrum (150MHz, DMSO-d6) of compound a14 40
Figure S14-3. HR MS of compound a14 41

Figure S15-1. ^{1}H NMR spectrum (600MHz, DMSO-d6) of compound a15 42
Figure S15-2. ^{13}C NMR spectrum (150MHz, DMSO-d6) of compound a15 43
Figure S15-3. HR MS of compound a15 44
Figure S16-1. ^{1}H NMR spectrum (600MHz, DMSO-d6) of compound a16 45
Figure S16-2. ^{13}C NMR spectrum (150MHz, DMSO-d6) of compound a16 46
Figure S16-3. HR MS of compound a16 47
Figure S17-1. ^{1}H NMR spectrum (600MHz, DMSO-d6) of compound a17 48
Figure S17-2. ^{13}C NMR spectrum (150MHz, DMSO-d6) of compound a17 49
Figure S17-3. HR MS of compound a17 50
Figure S18-1. ^{1}H NMR spectrum (600MHz, DMSO-d6) of compound a18 51
Figure S18-2. ^{13}C NMR spectrum (150MHz, DMSO-d6) of compound a18 52
Figure S18-3. HR MS of compound a18 53
Figure S19-1. ^{1}H NMR spectrum (600MHz, DMSO-d6) of compound a19 54
Figure S19-2. HR MS of compound a19 55
Figure S20-1. ^{1}H NMR spectrum (600MHz, DMSO-d6) of compound a20 56
Figure S20-2. ^{13}C NMR spectrum (150MHz, DMSO-d6) of compound a20 57
Figure S20-3. HR MS of compound a20 58
Figure S21-1. ^{1}H NMR spectrum (600MHz, DMSO-d6) of compound a21 59
Figure S21-2. ^{13}C NMR spectrum (150MHz, DMSO-d6) of compound a21 60
Figure S21-3. HR MS of compound a21....61
Figure S22-1. ^{1}H NMR spectrum (600MHz, DMSO-d6) of compound a22 62
Figure S22-2. ^{13}C NMR spectrum (150MHz, DMSO-d6) of compound a22 63
Figure S22-3. HR MS of compound a22 64

Figure S1-1. ^{1}H NMR spectrum (600MHz, DMSO-d6) of compound a1

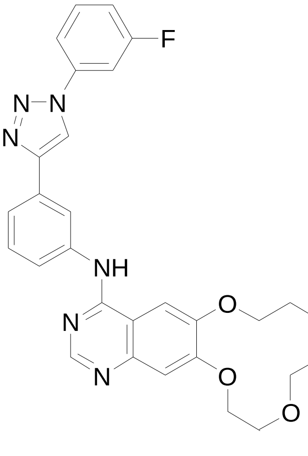

a1

Chemical Formula: $C_{28}H_{25}FN_6O_4$
Exact Mass: 528.19

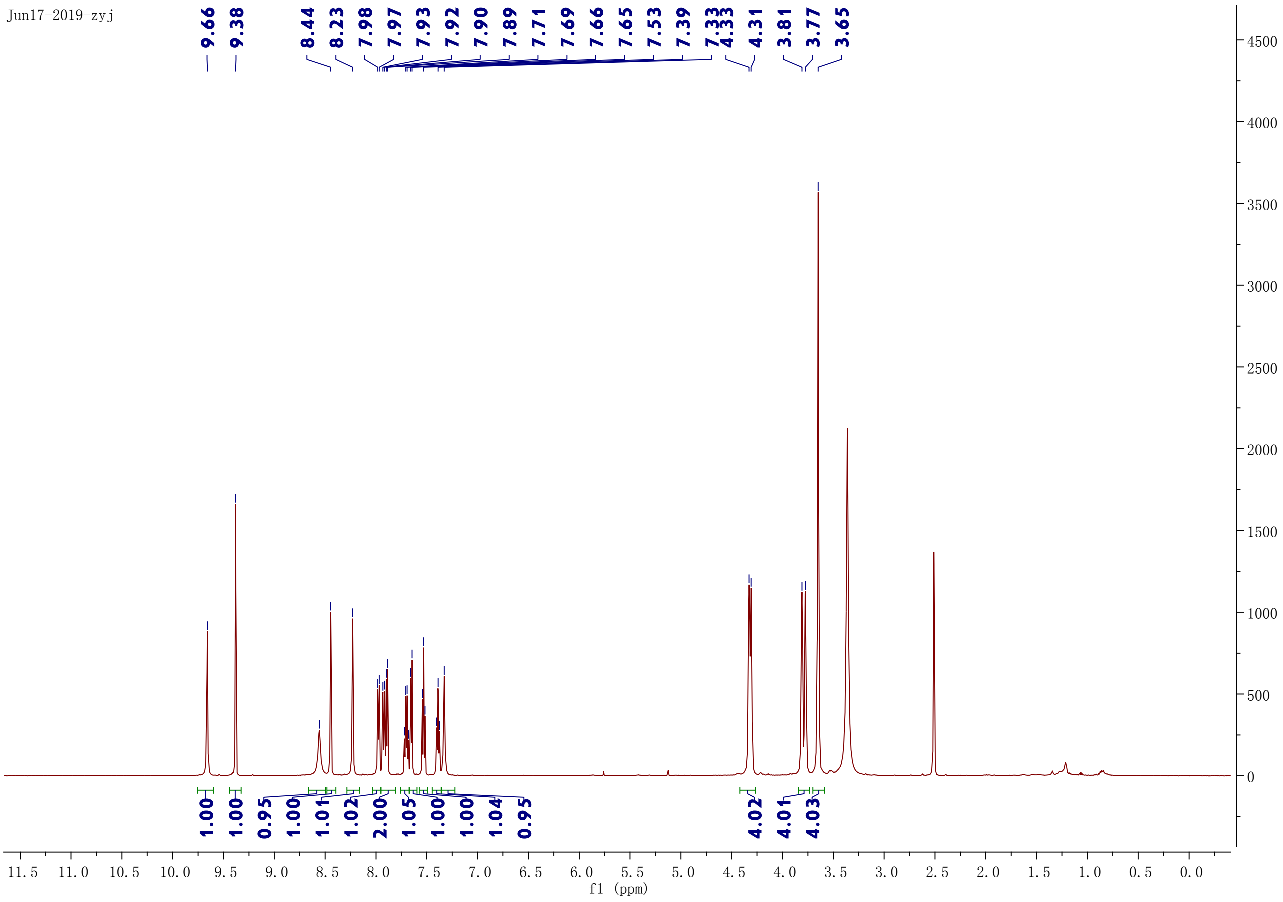

Figure S1-2. ^{13}C NMR spectrum (150MHz, DMSO-d6) of compound a1

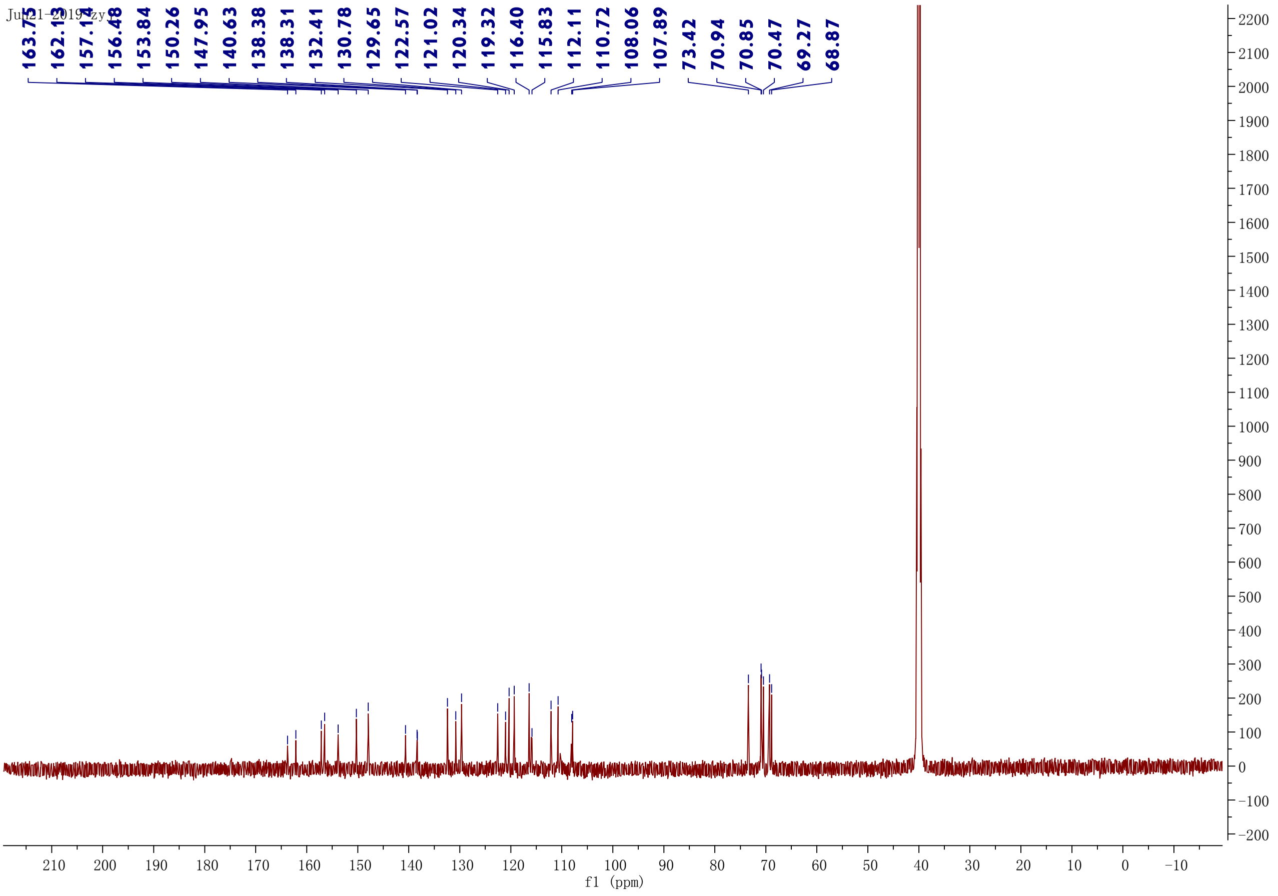

Figure S1-3. HR MS of compound a1

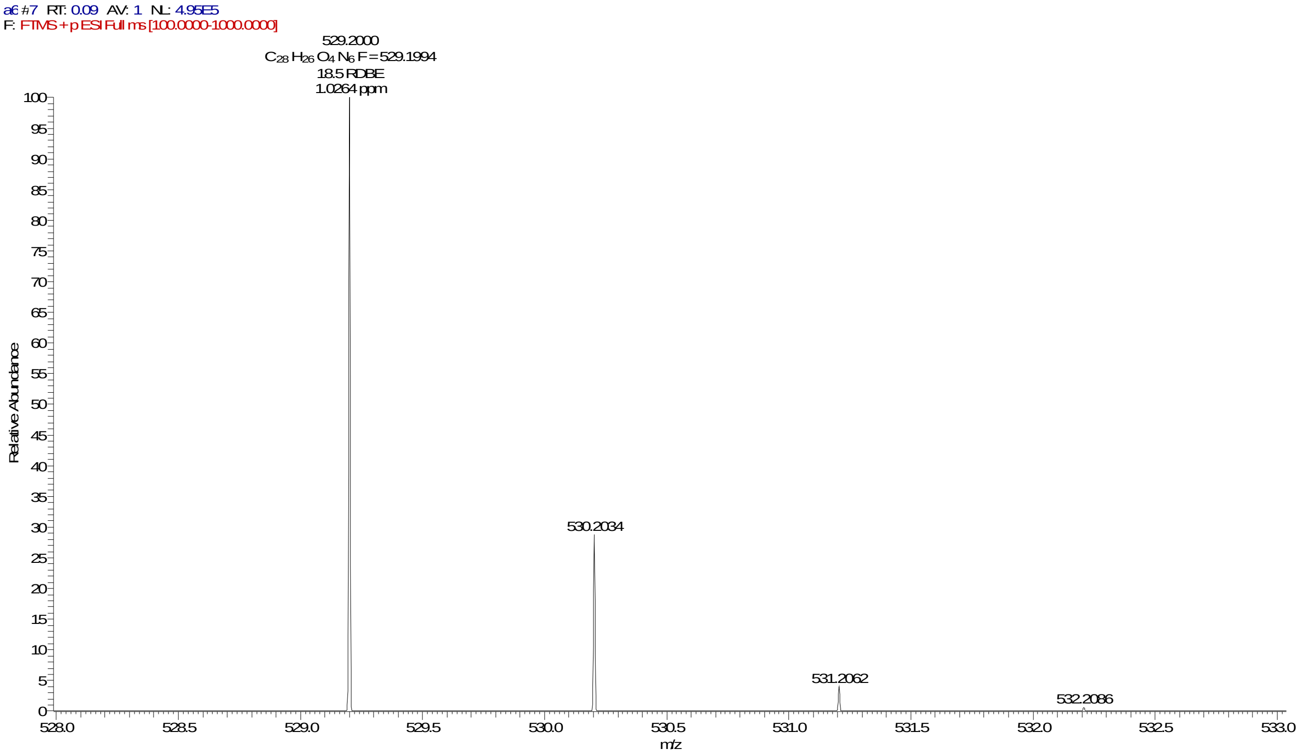

Figure S2-1. ^{1}H NMR spectrum (600MHz, DMSO-d6) of compound a2

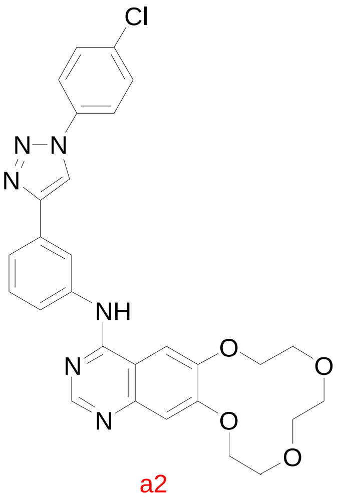

a2

Chemical Formula: $C_{28}H_{25}ClN_6O_4$
Exact Mass: 544.16

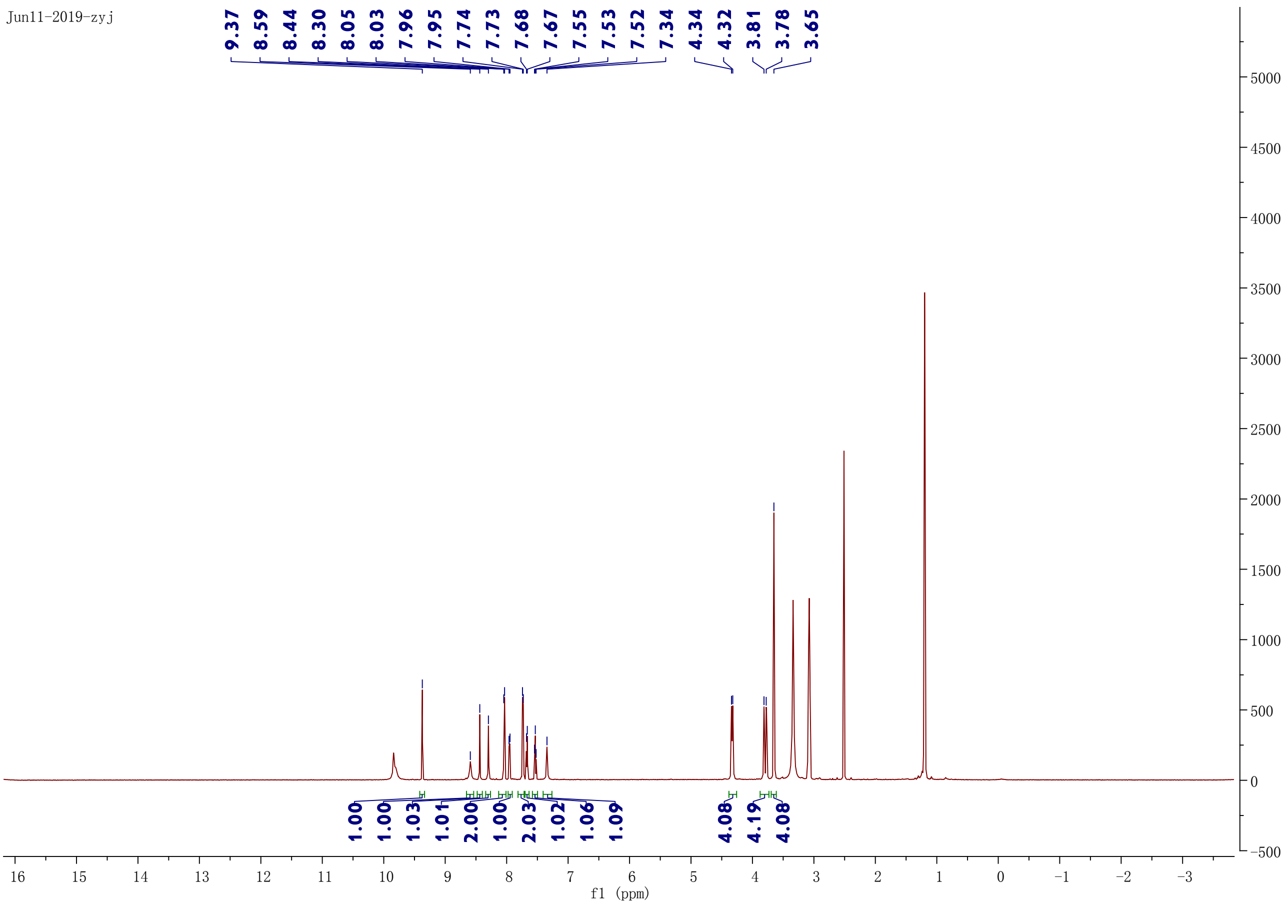

Figure S2-2. HR MS of compound a2

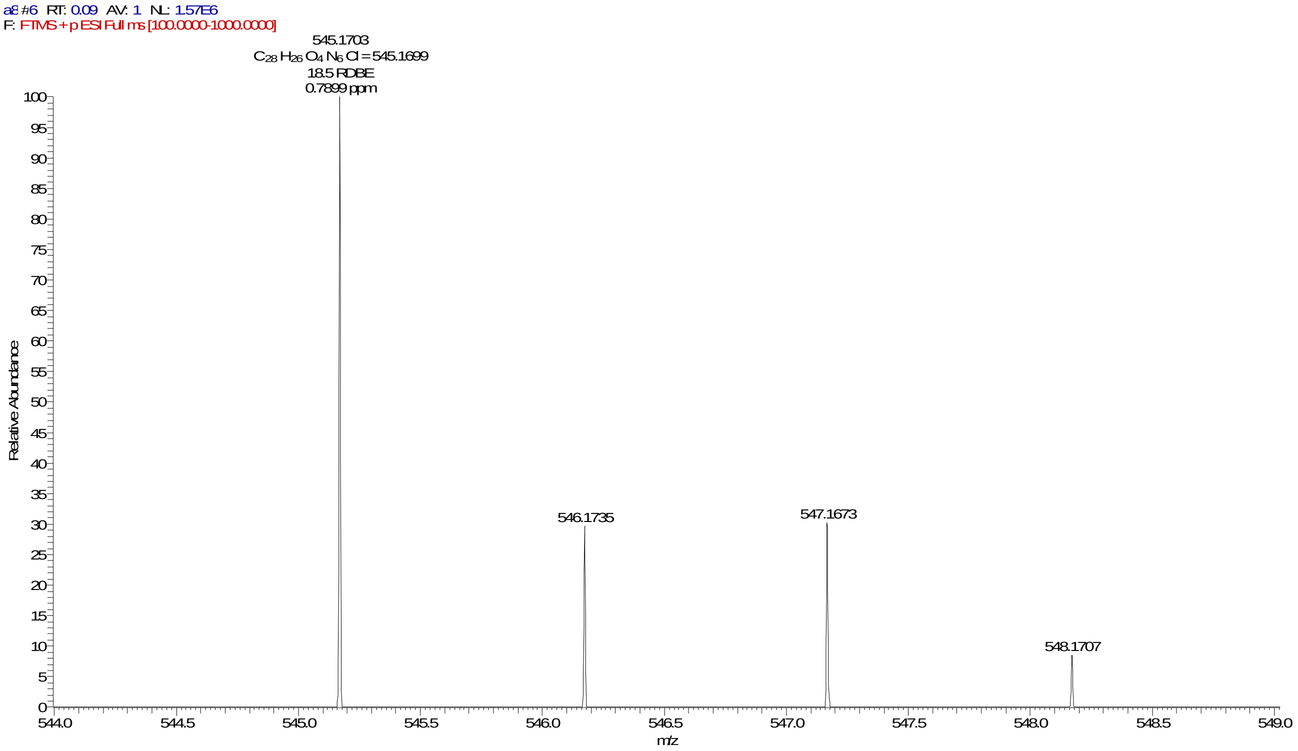

Figure S3-1. ^{1}H NMR spectrum (600MHz, DMSO-d6) of compound a3

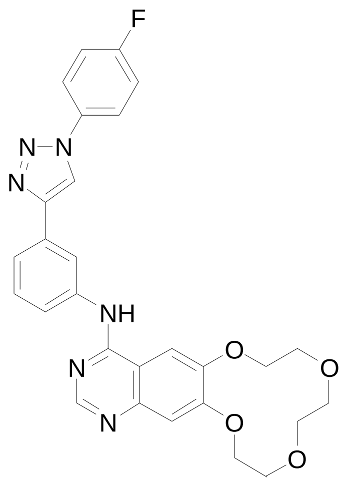

a3

Chemical Formula: $C_{28}H_{25}FN_6O_4$
Exact Mass: 528.19

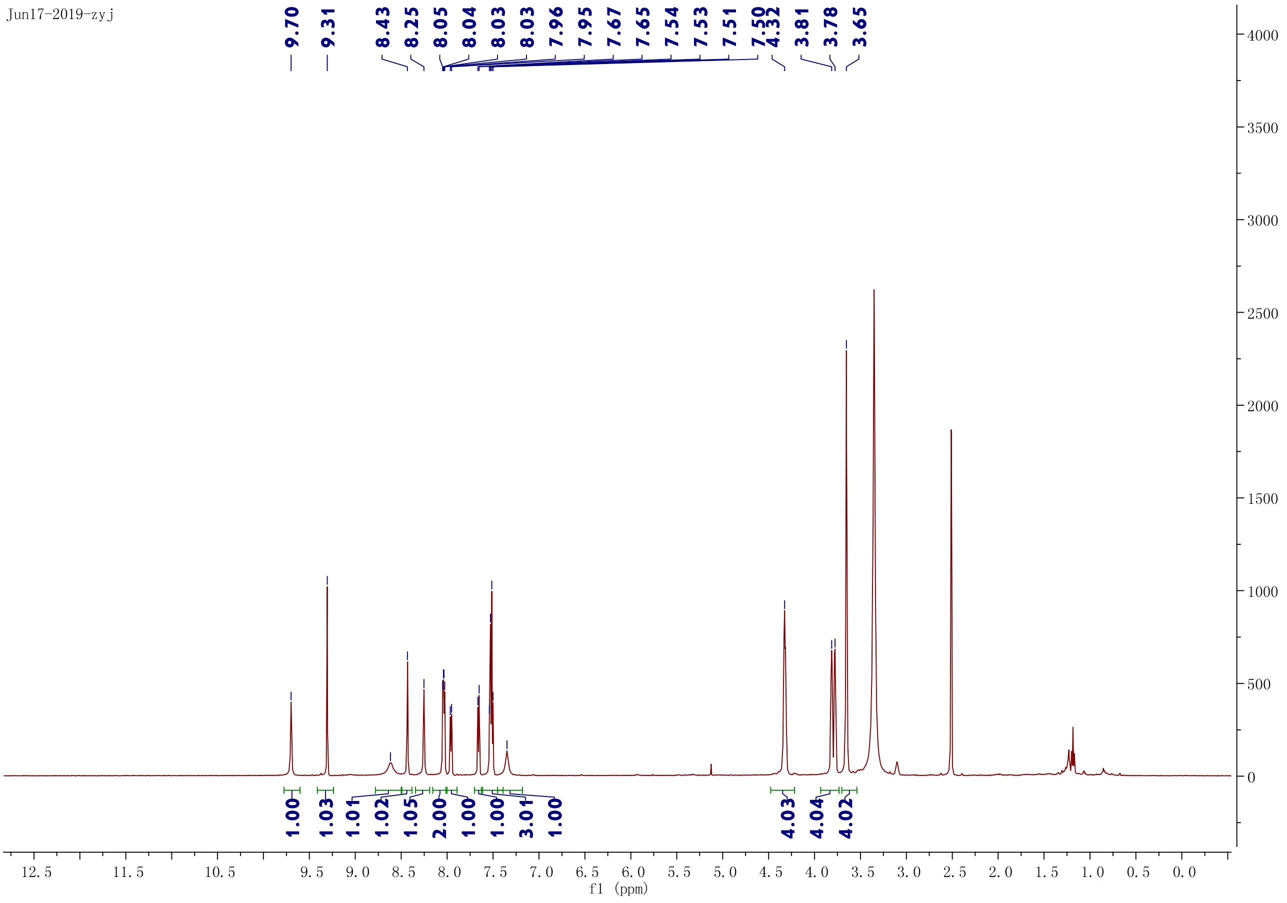

Figure S3-2. ^{13}C NMR spectrum (150MHz, DMSO-d6) of compound a3

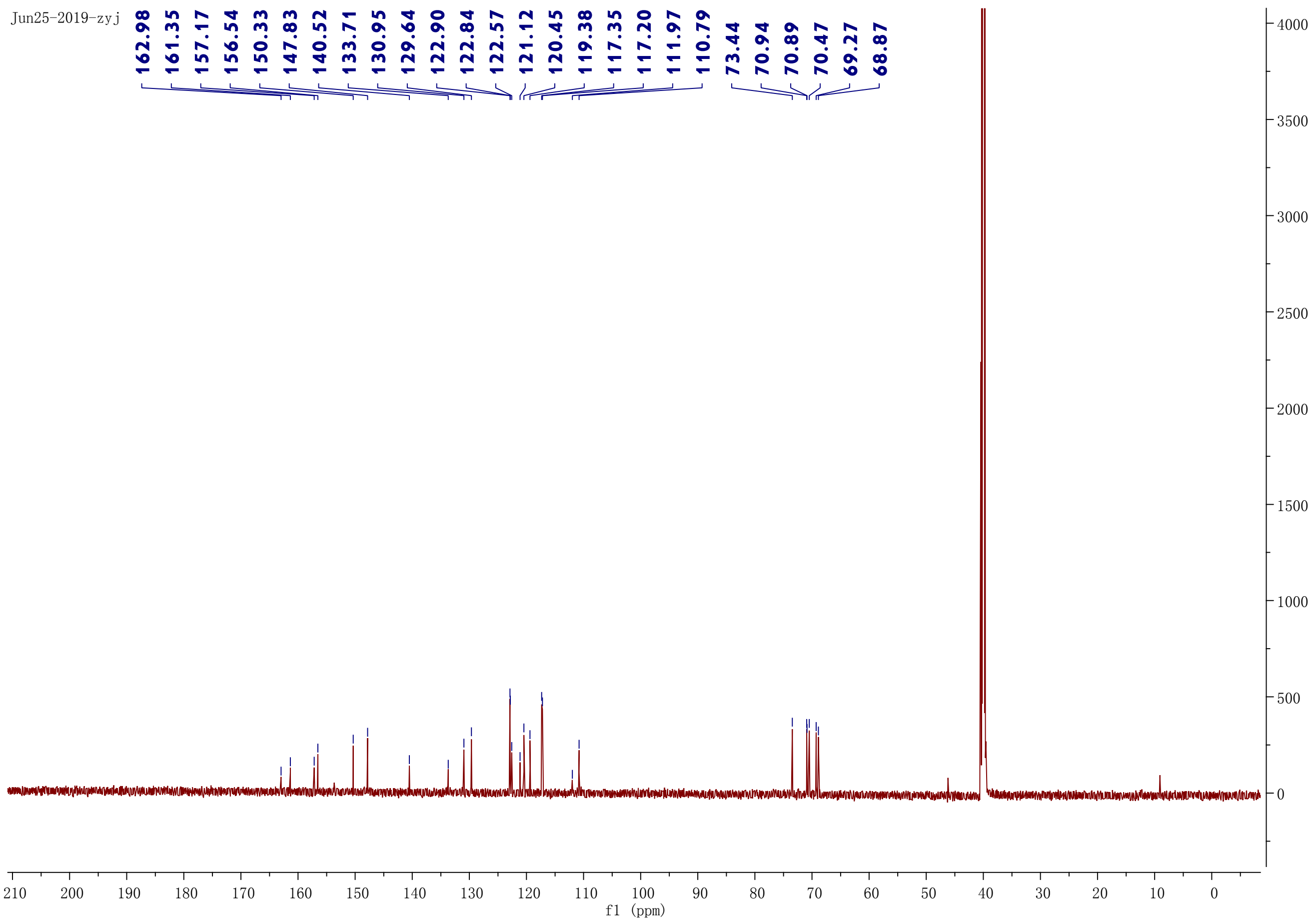

Figure S3-3. HR MS of compound a3

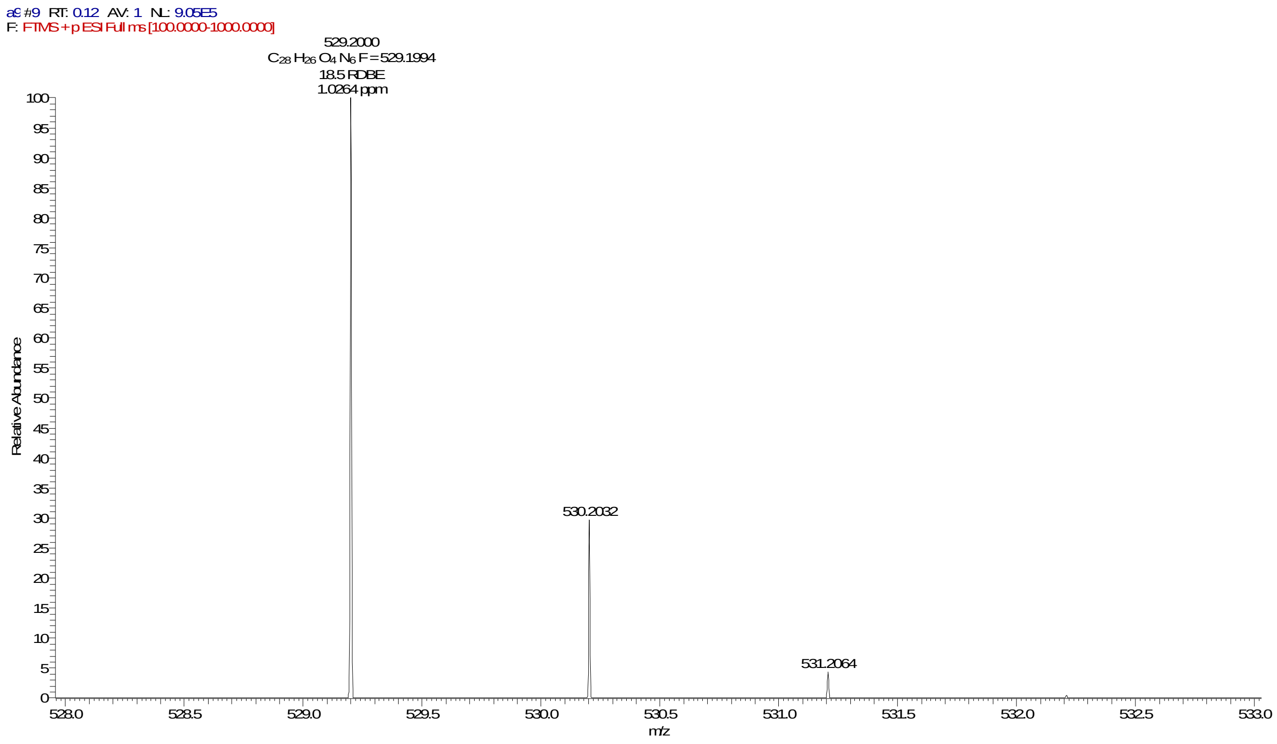

Figure S4-1. ^{1}H NMR spectrum (600MHz, DMSO-d6) of compound a4

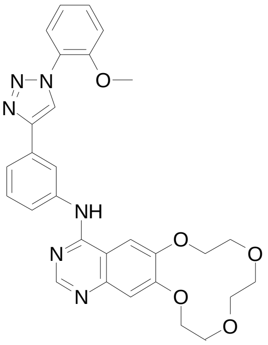

Chemical Formula: $C_{29}H_{28}N_6O_5$
Exact Mass: 540.21

a4

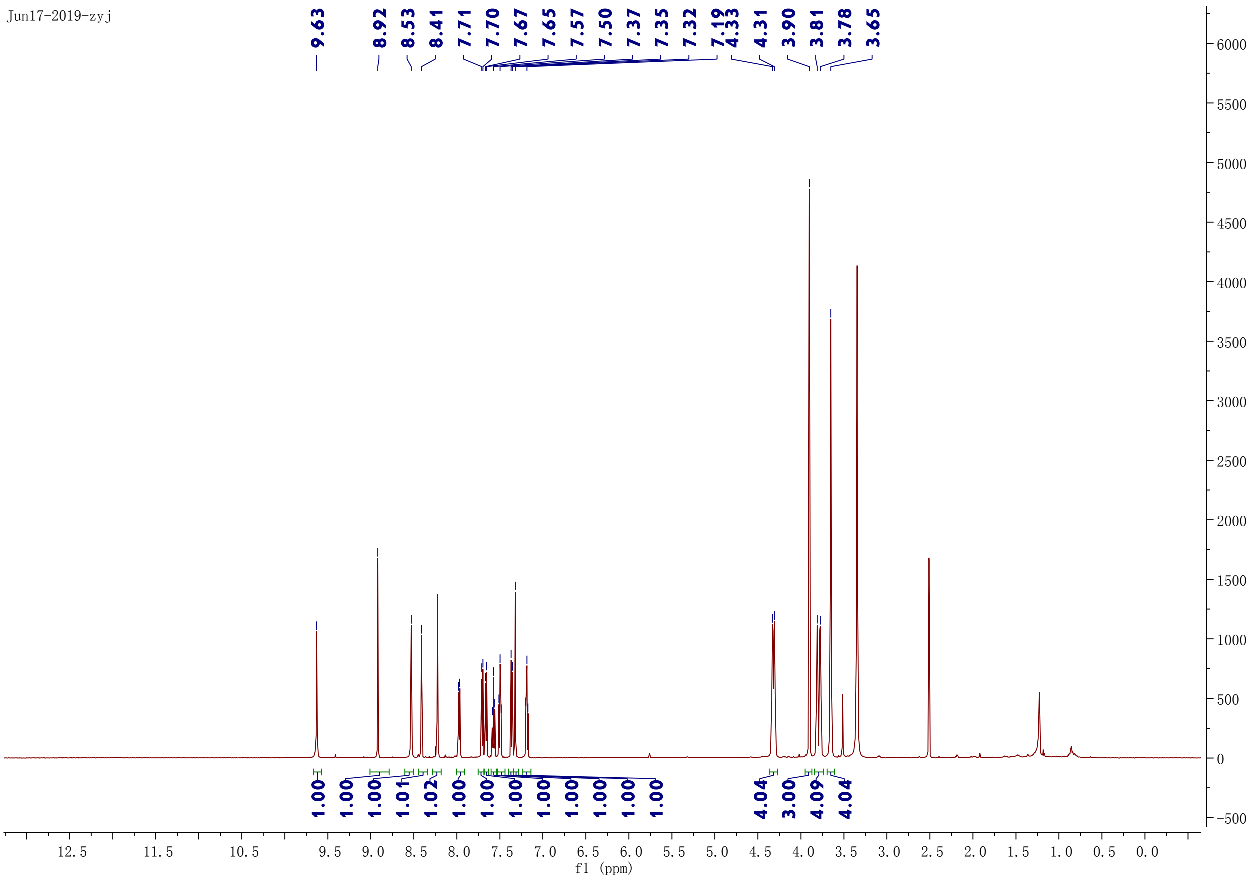

Figure S4-2. ^{13}C NMR spectrum (150MHz, DMSO-d6) of compound a4

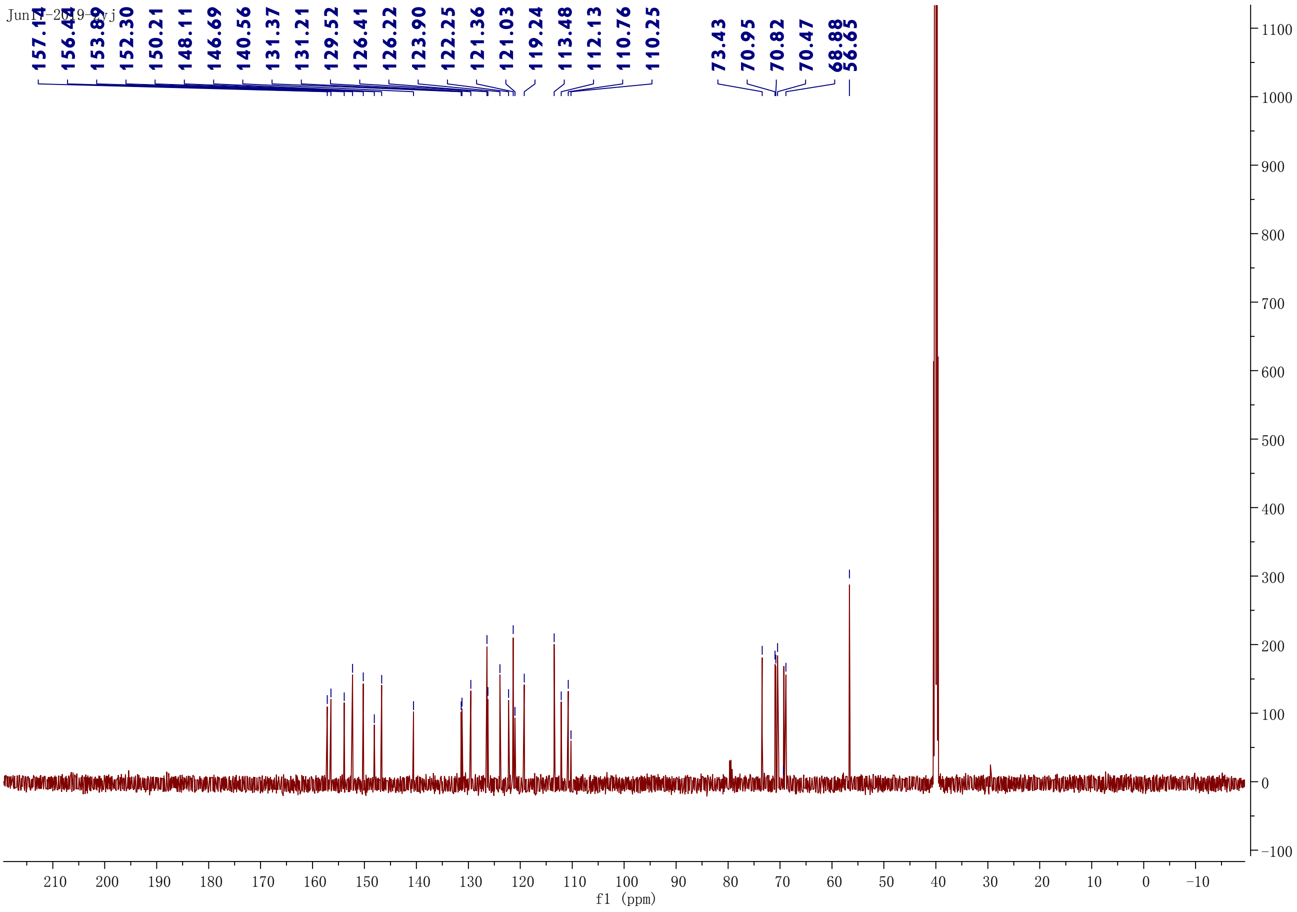

Figure S4-3. HR MS of compound a4

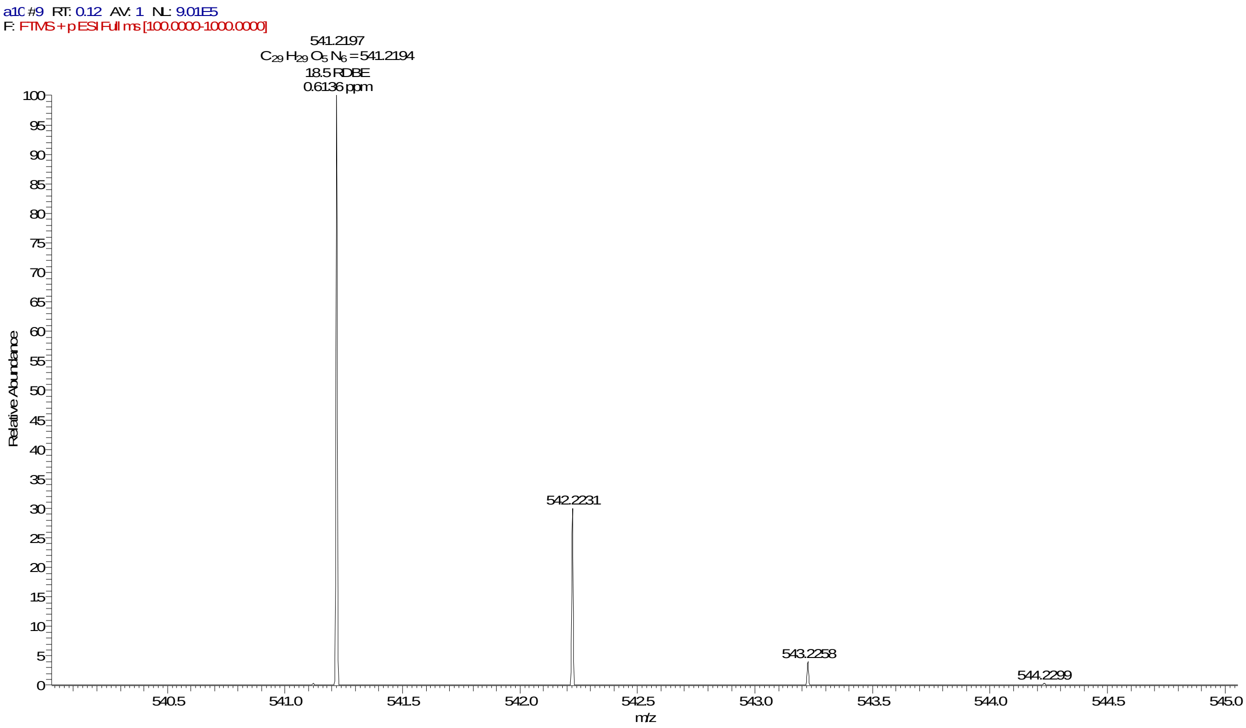

Figure S5-1. ^{1}H NMR spectrum (600MHz, DMSO-d6) of compound a5

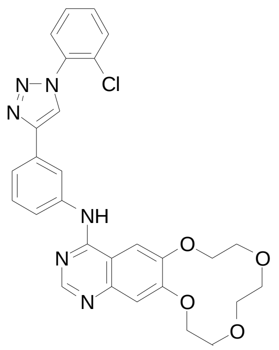

Chemical Formula: $C_{28}H_{25}ClN_6O_4$
Exact Mass: 544.16

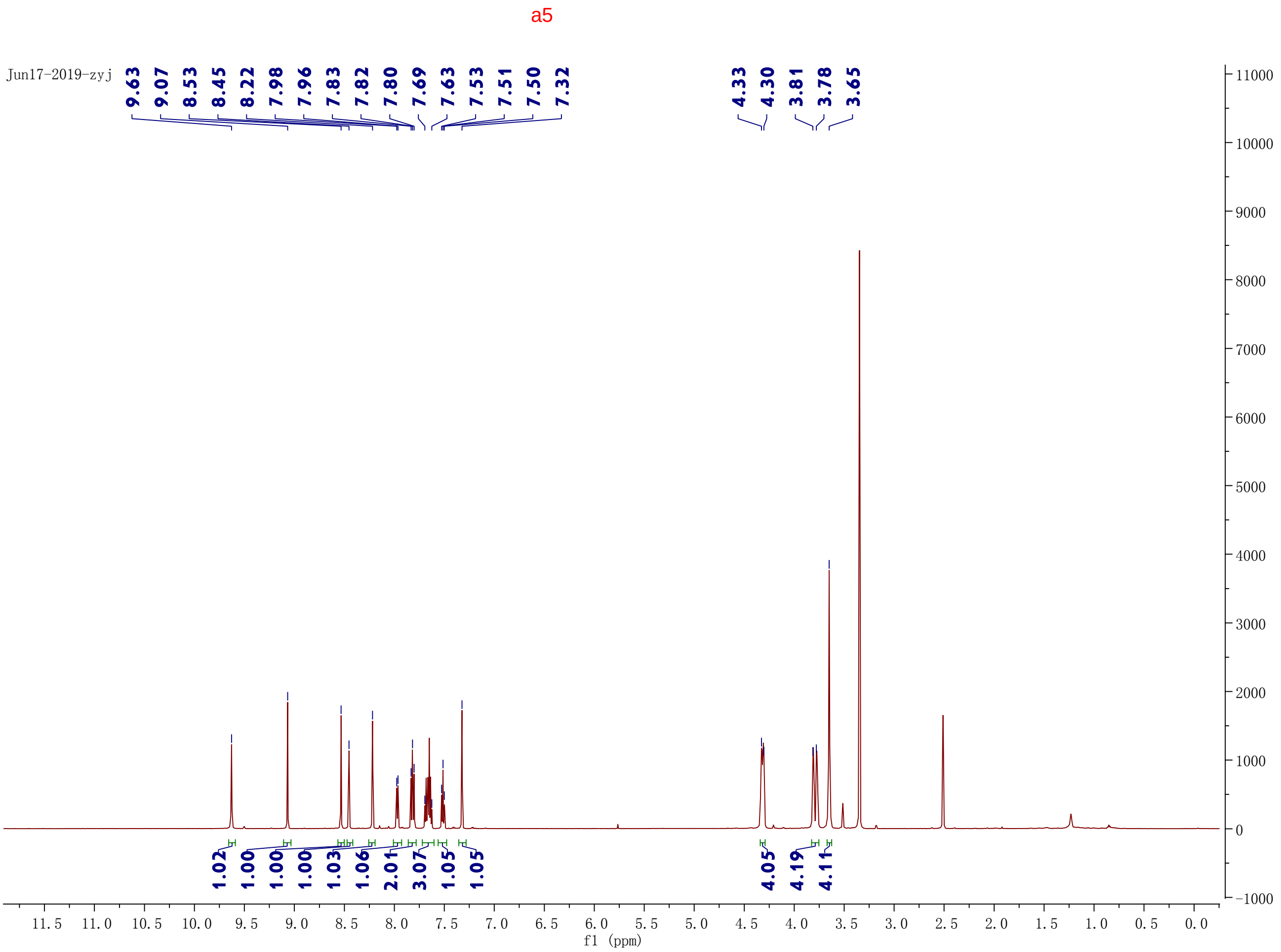

Figure S5-2. ^{13}C NMR spectrum (150MHz, DMSO-d6) of compound a5

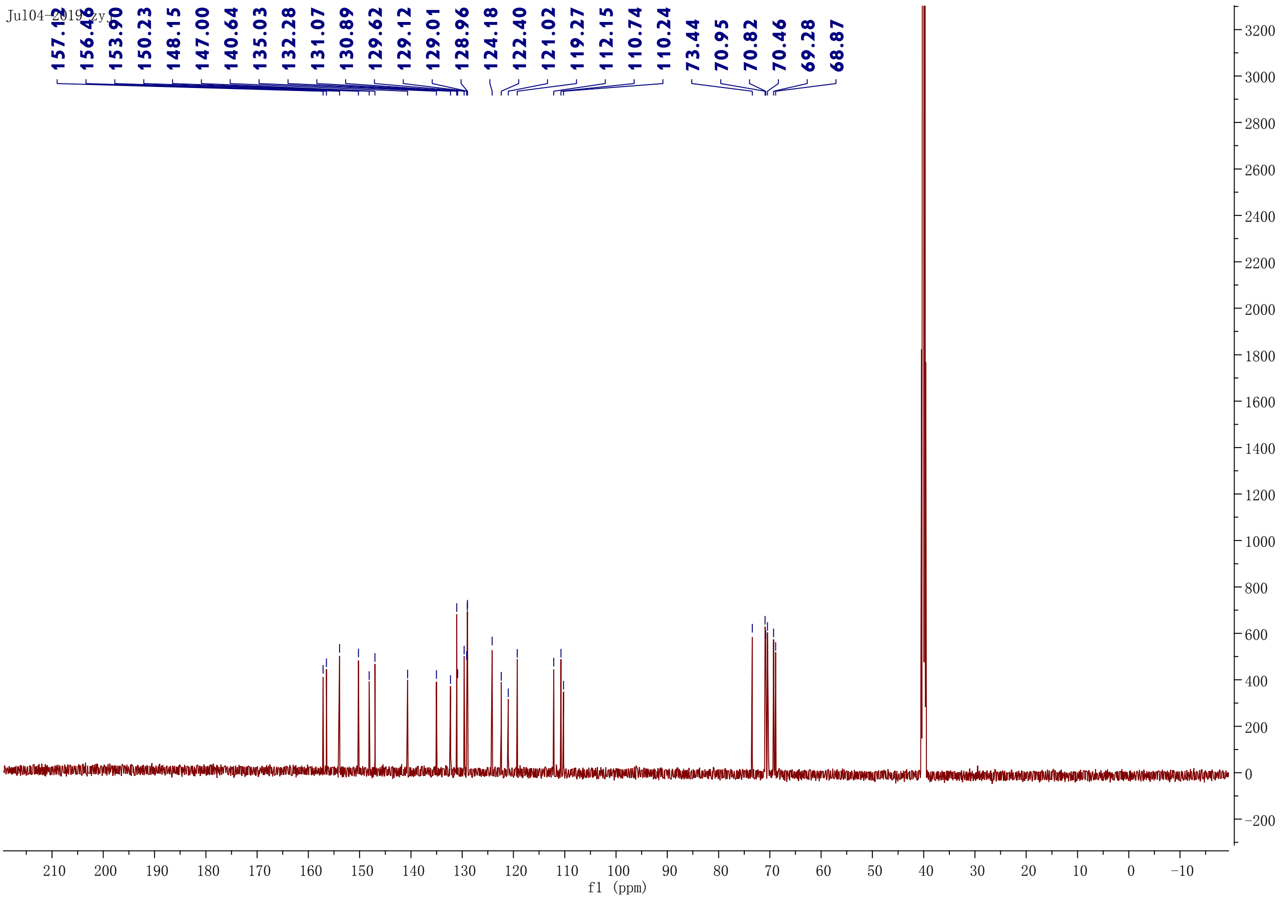

Figure S5-3. HR MS of compound a5

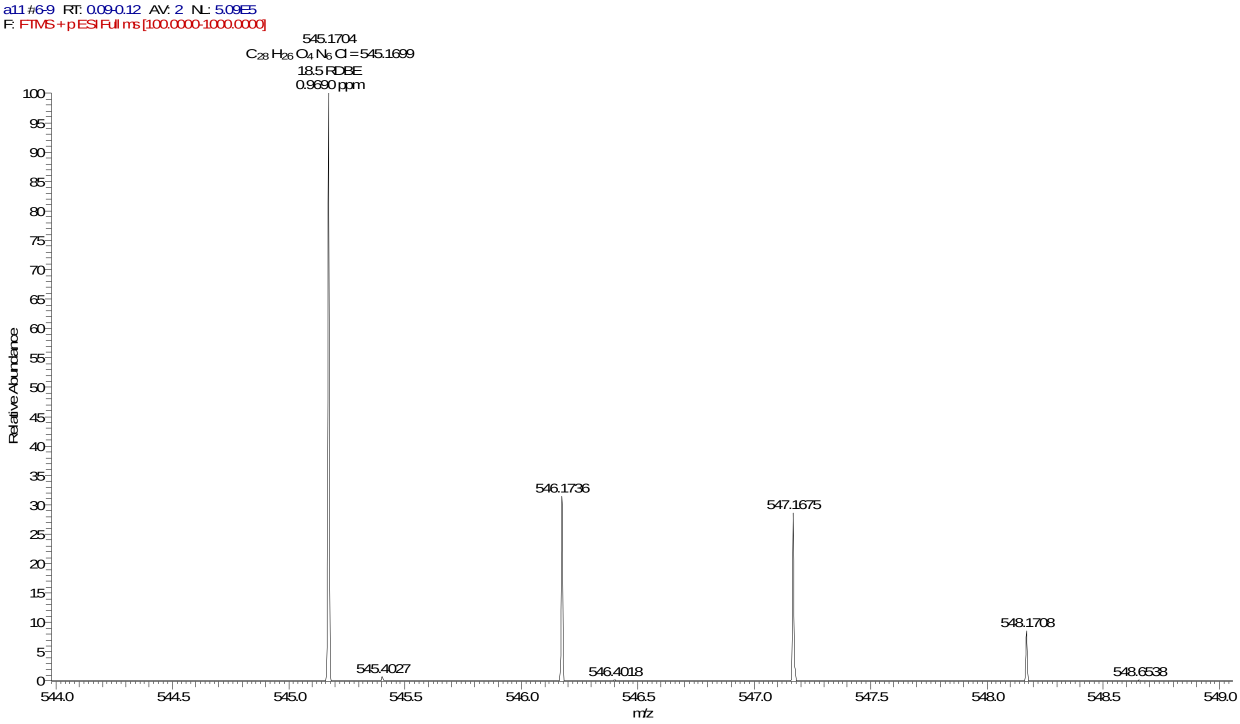

Figure S6-1. ^{1}H NMR spectrum (600MHz, DMSO-d6) of compound a6

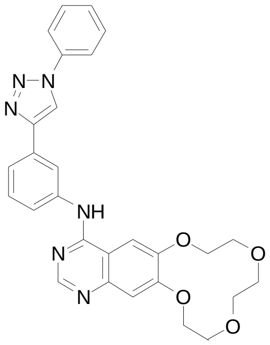

Chemical Formula: $C_{28}H_{26}N_6O_4$
Exact Mass: 510.20

a6

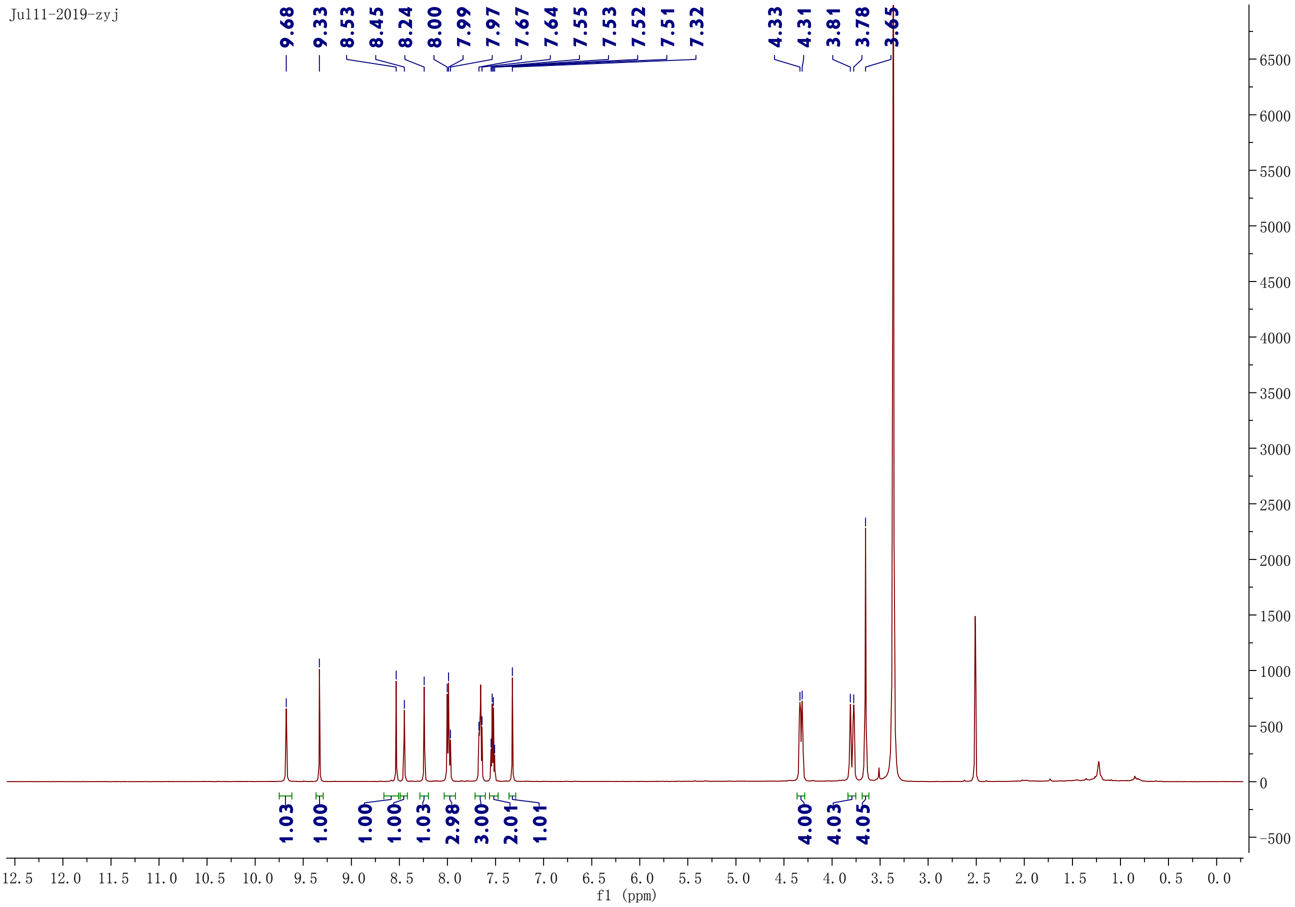

Figure S6-2. ^{13}C NMR spectrum (150MHz, DMSO-d6) of compound a6

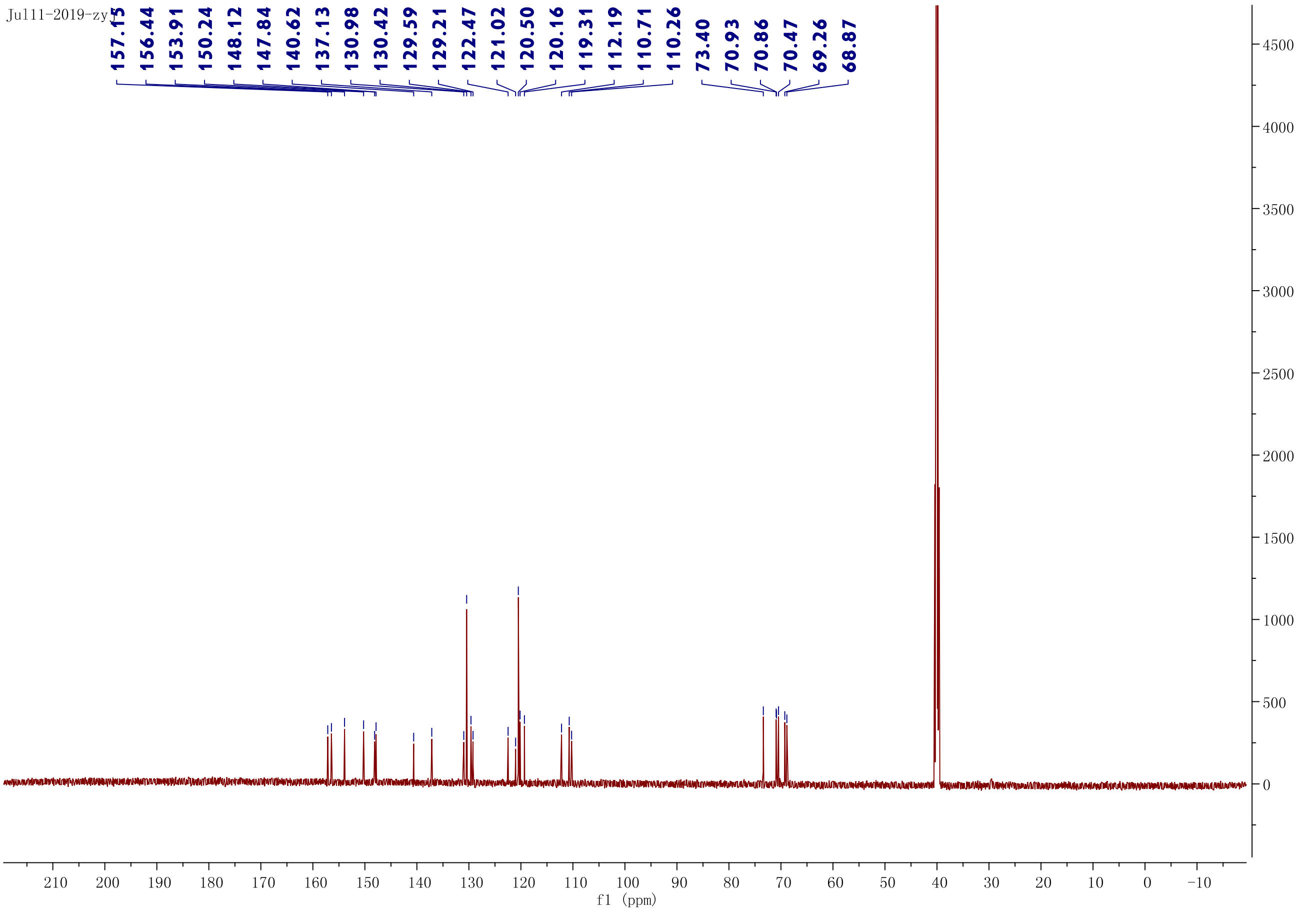

Figure S6-3. HR MS of compound a6

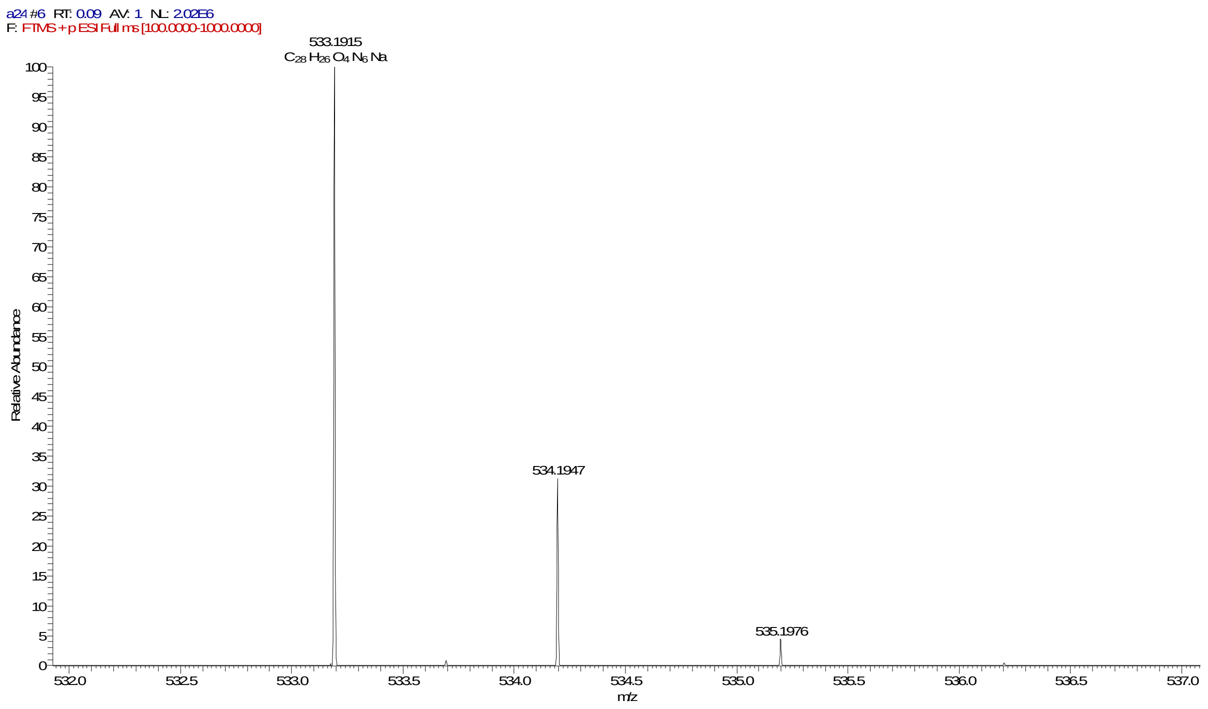

Figure S7-1. ^{1}H NMR spectrum (600MHz, DMSO-d6) of compound a7

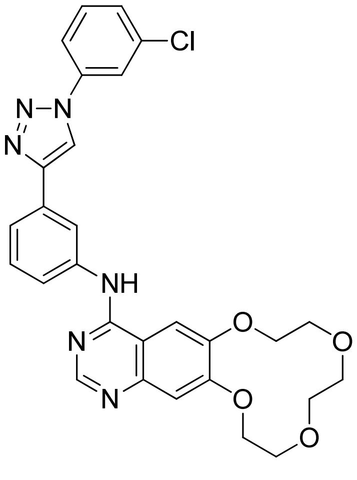

a7

Chemical Formula: $C_{28}H_{25}ClN_6O_4$

Exact Mass: 544.16

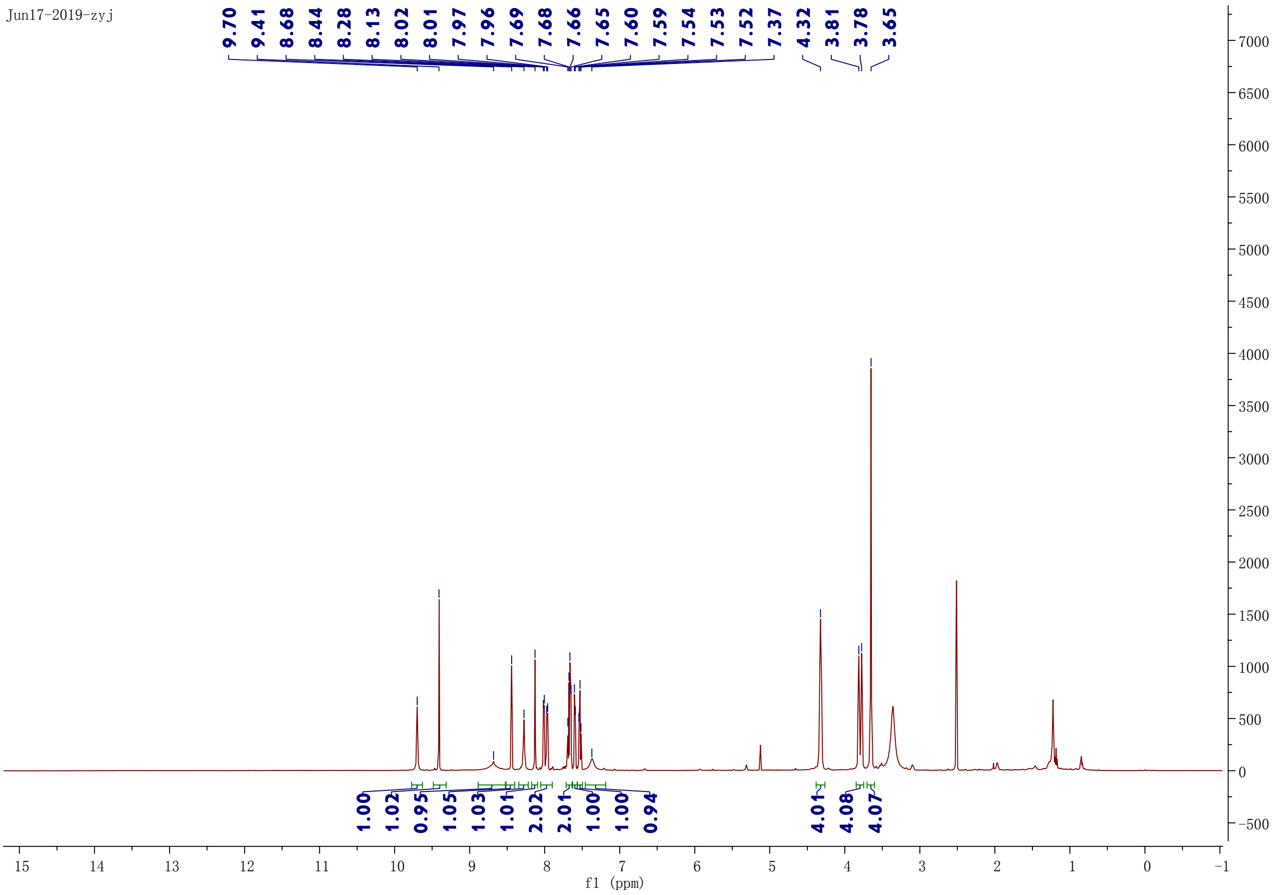

Figure S7-2. ^{13}C NMR spectrum (150MHz, DMSO-d6) of compound a7

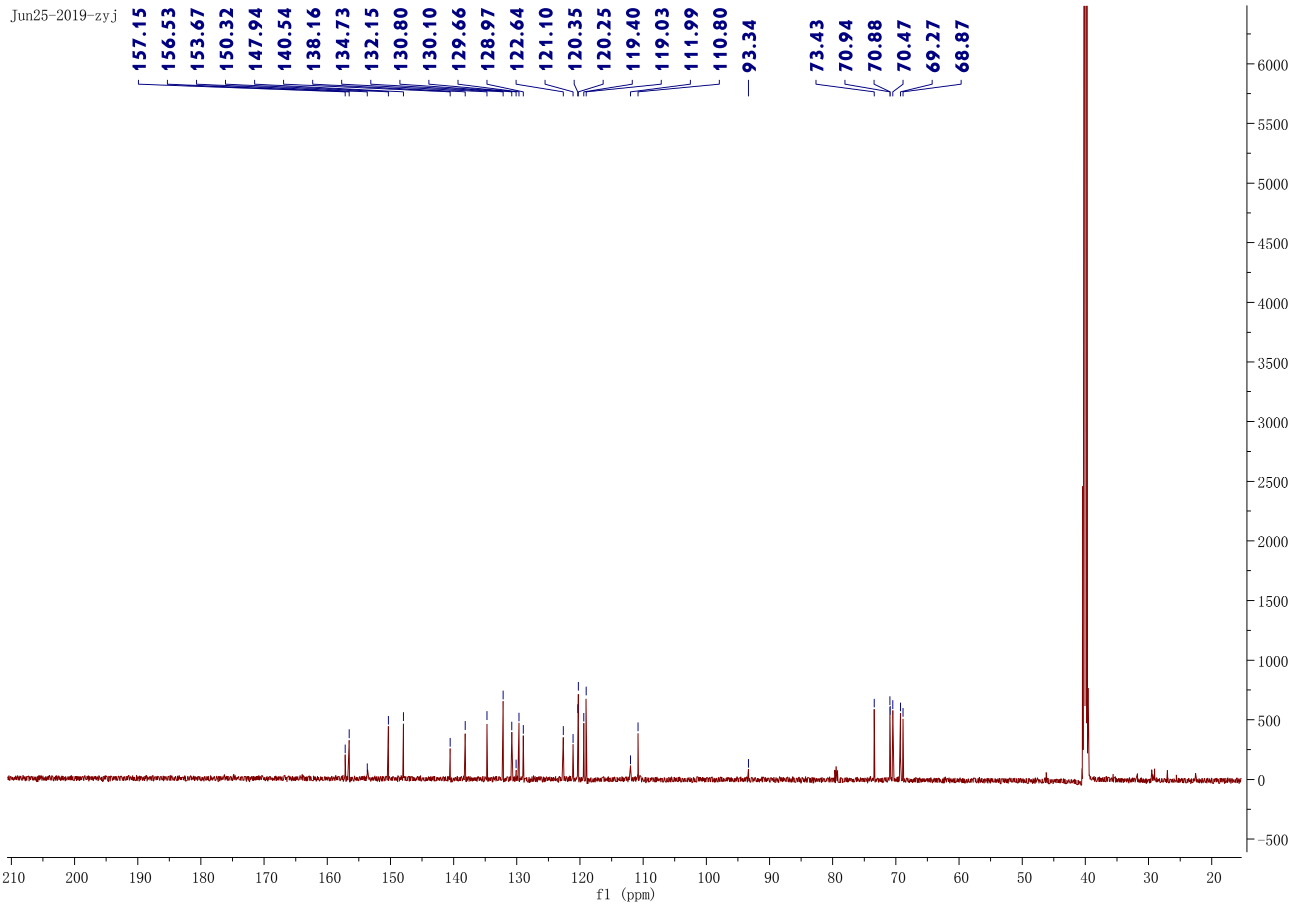

Figure S7-3. HR MS of compound a7

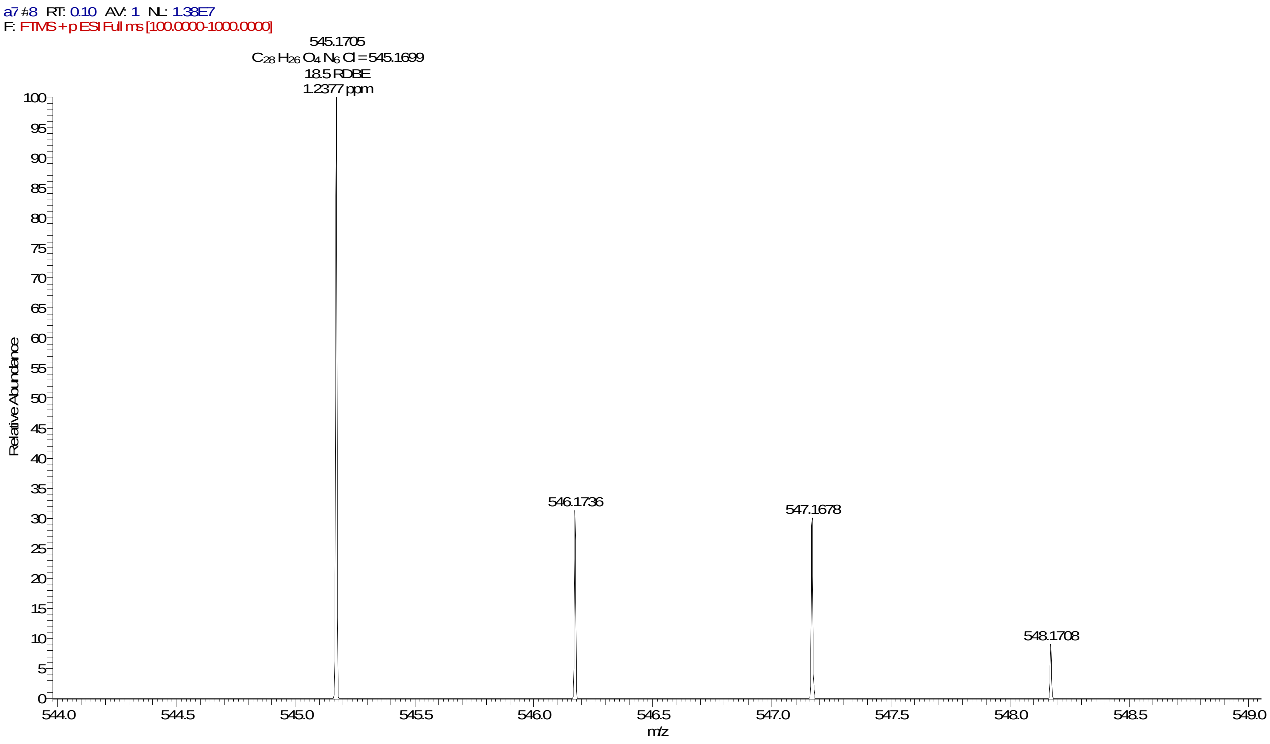

Figure S8-1. ^{1}H NMR spectrum (600MHz, DMSO-d6) of compound a8

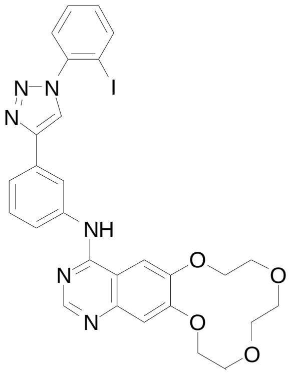

Chemical Formula: $C_{28}H_{25}IN_6O_4$
Exact Mass: 636.10

a8

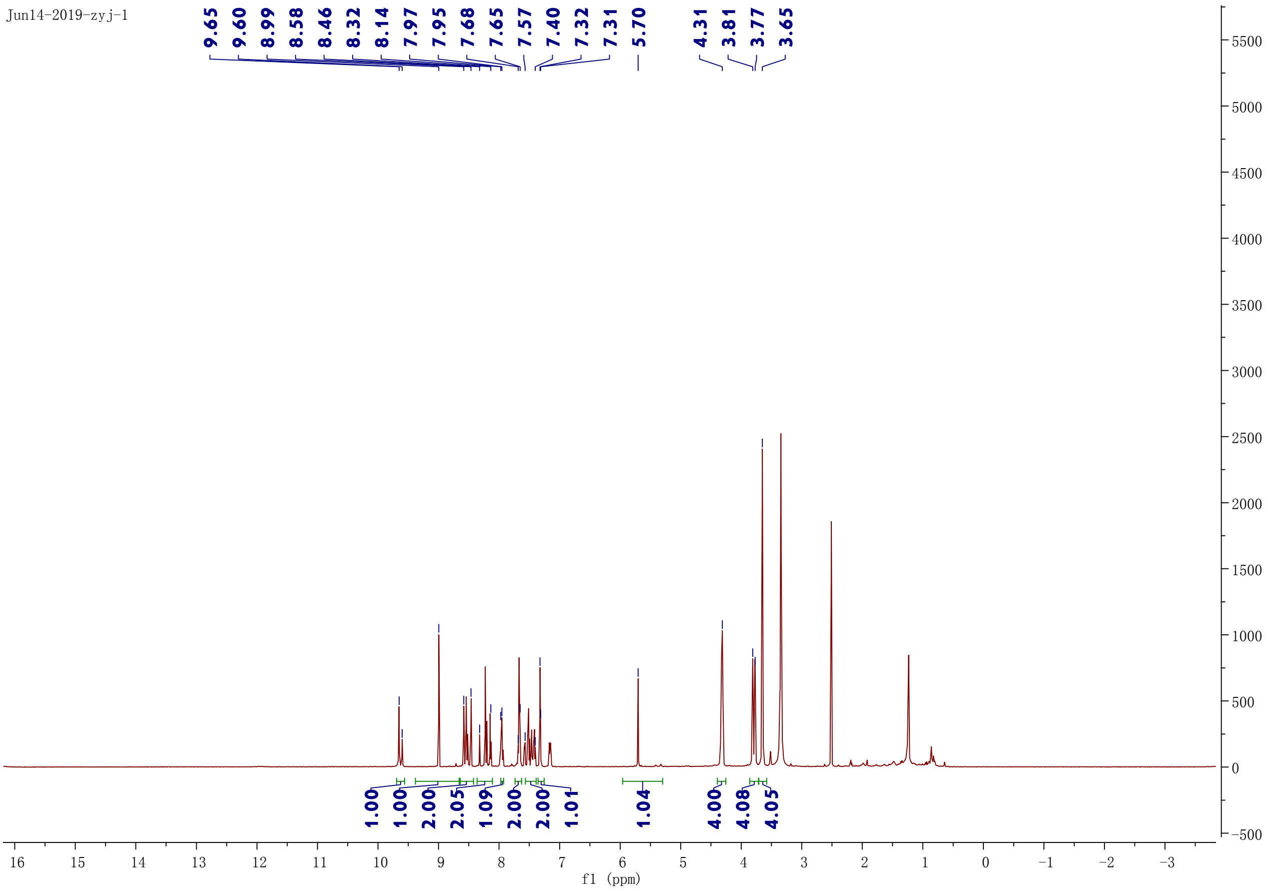

Figure S8-2. ^{13}C NMR spectrum (150MHz, DMSO-d6) of compound a8

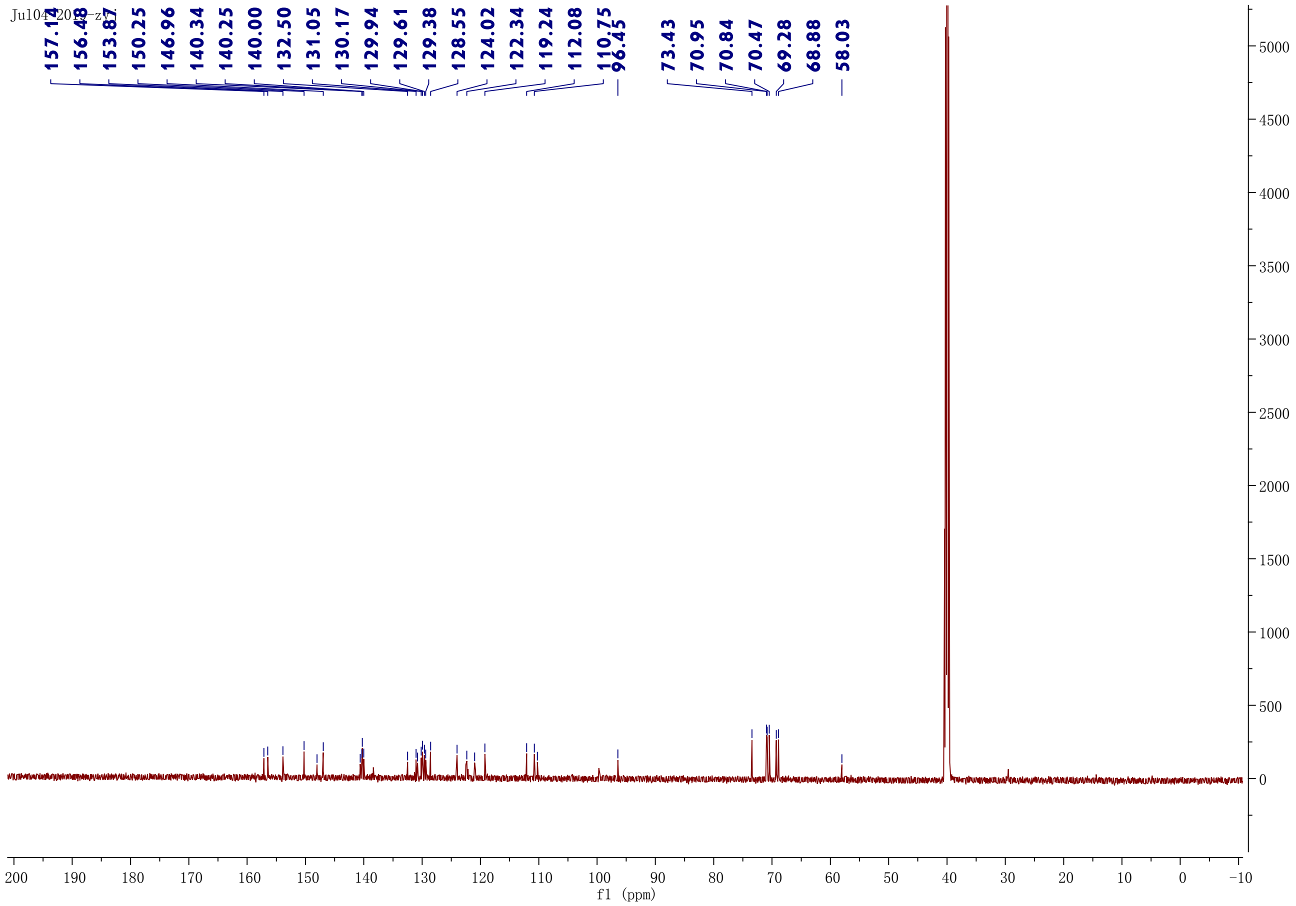

Figure S8-3. HR MS of compound a8

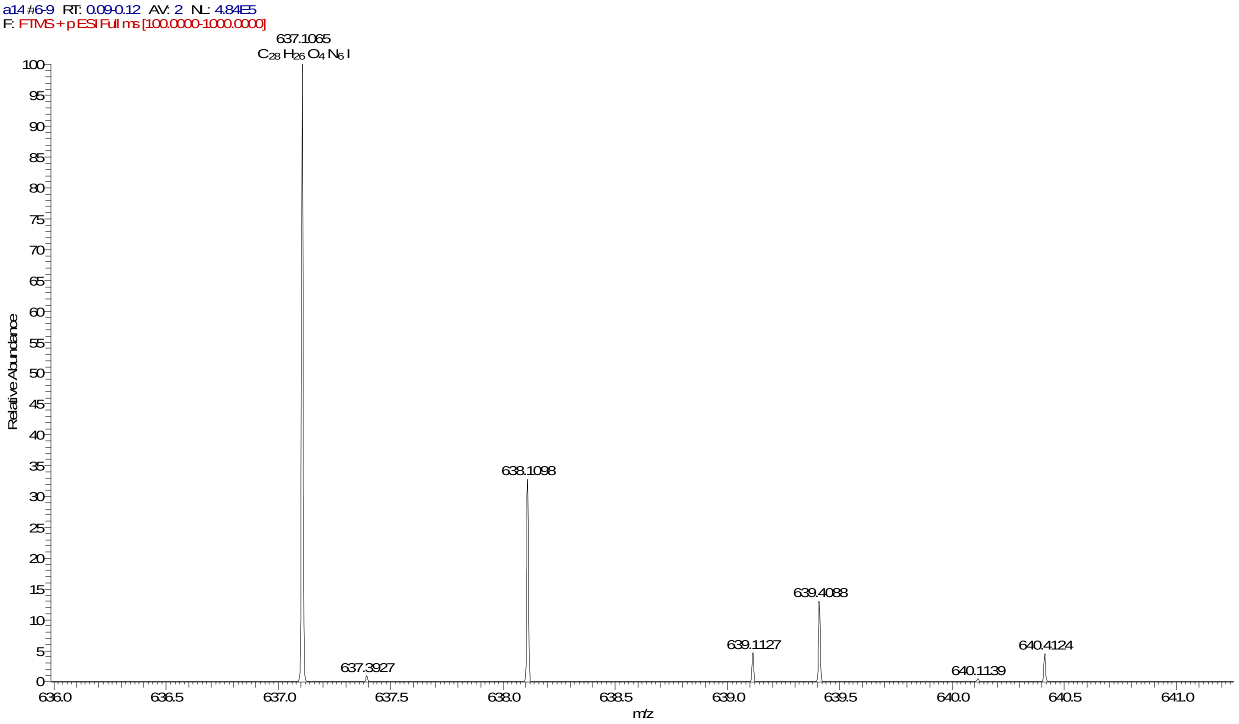

Figure S9-1. ^{1}H NMR spectrum (600MHz, DMSO-d6) of compound a9

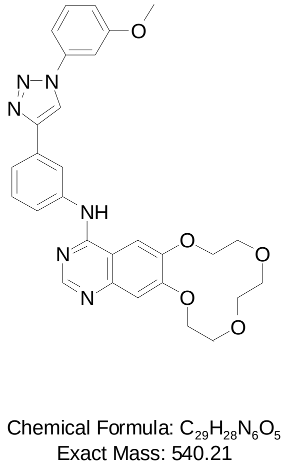

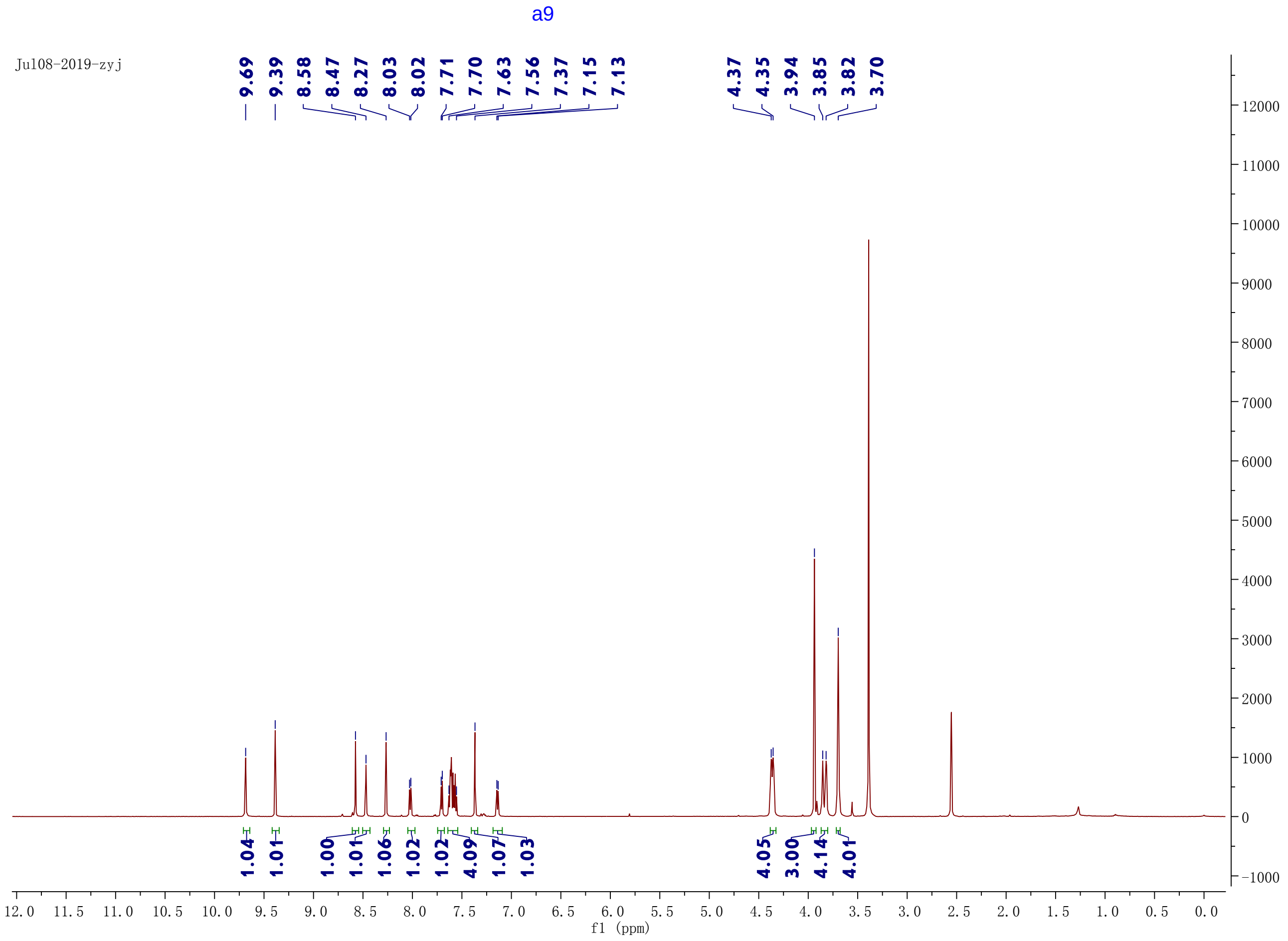

Figure S9-2. ^{13}C NMR spectrum (150MHz, DMSO-d6) of compound a9

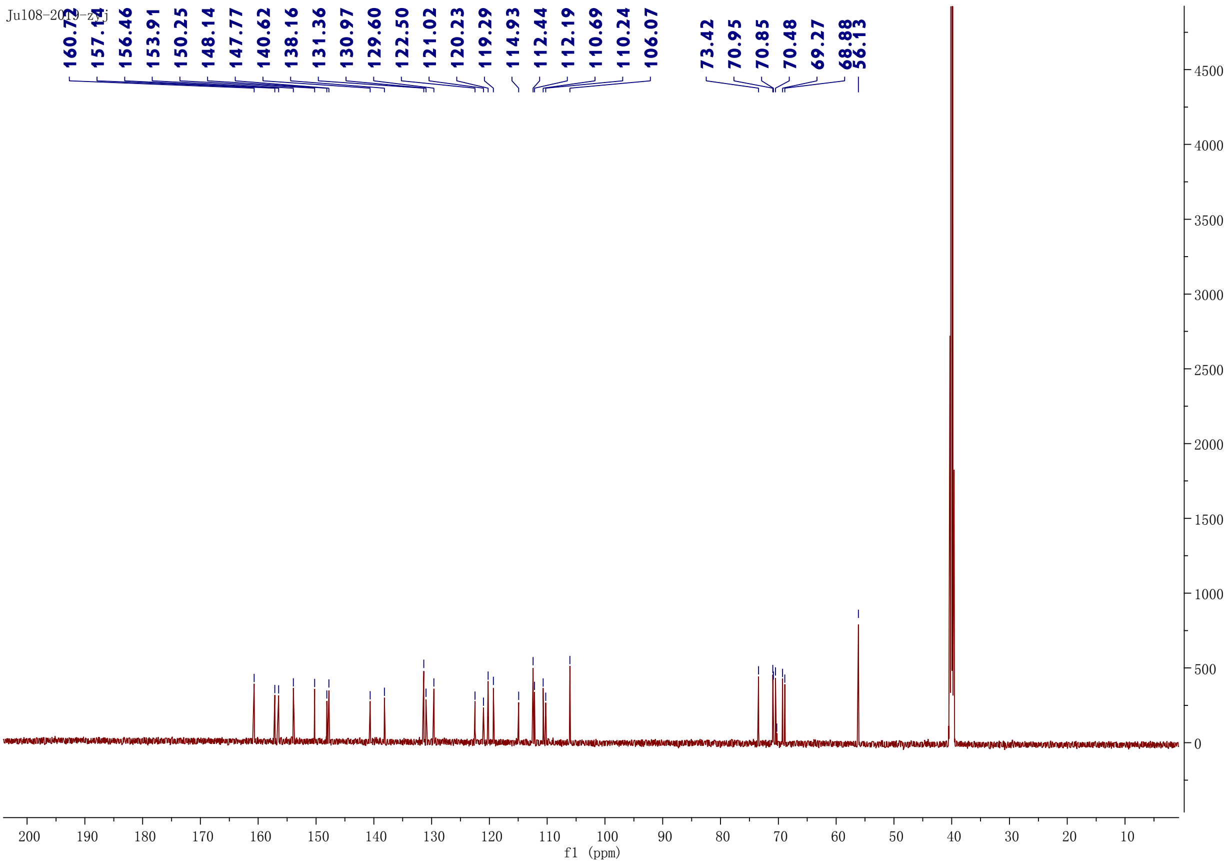

Figure S9-3. HR MS of compound a9

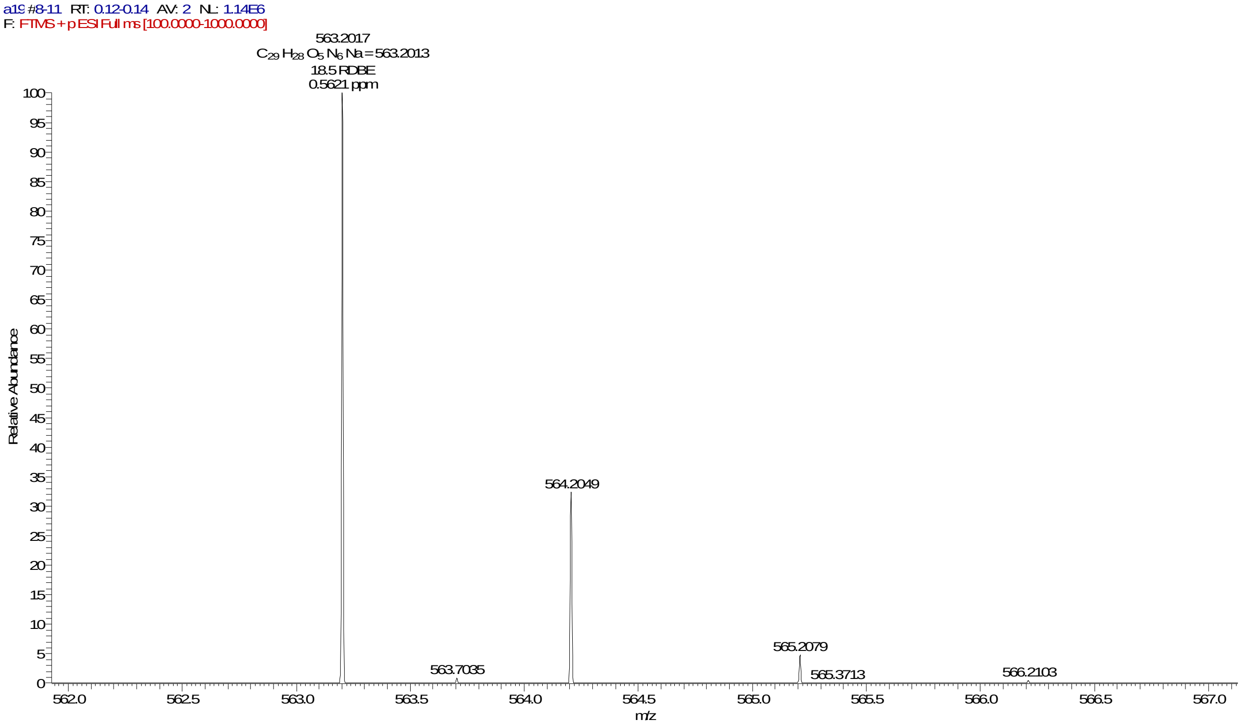

Figure S10-1. ^{1}H NMR spectrum (600MHz, DMSO-d6) of compound a10

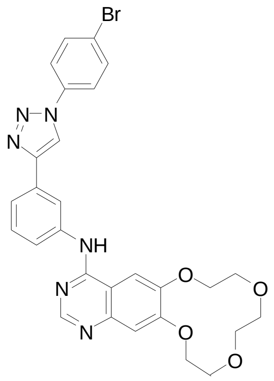

Chemical Formula: $C_{28}H_{25}BrN_6O_4$
Exact Mass: 588.11

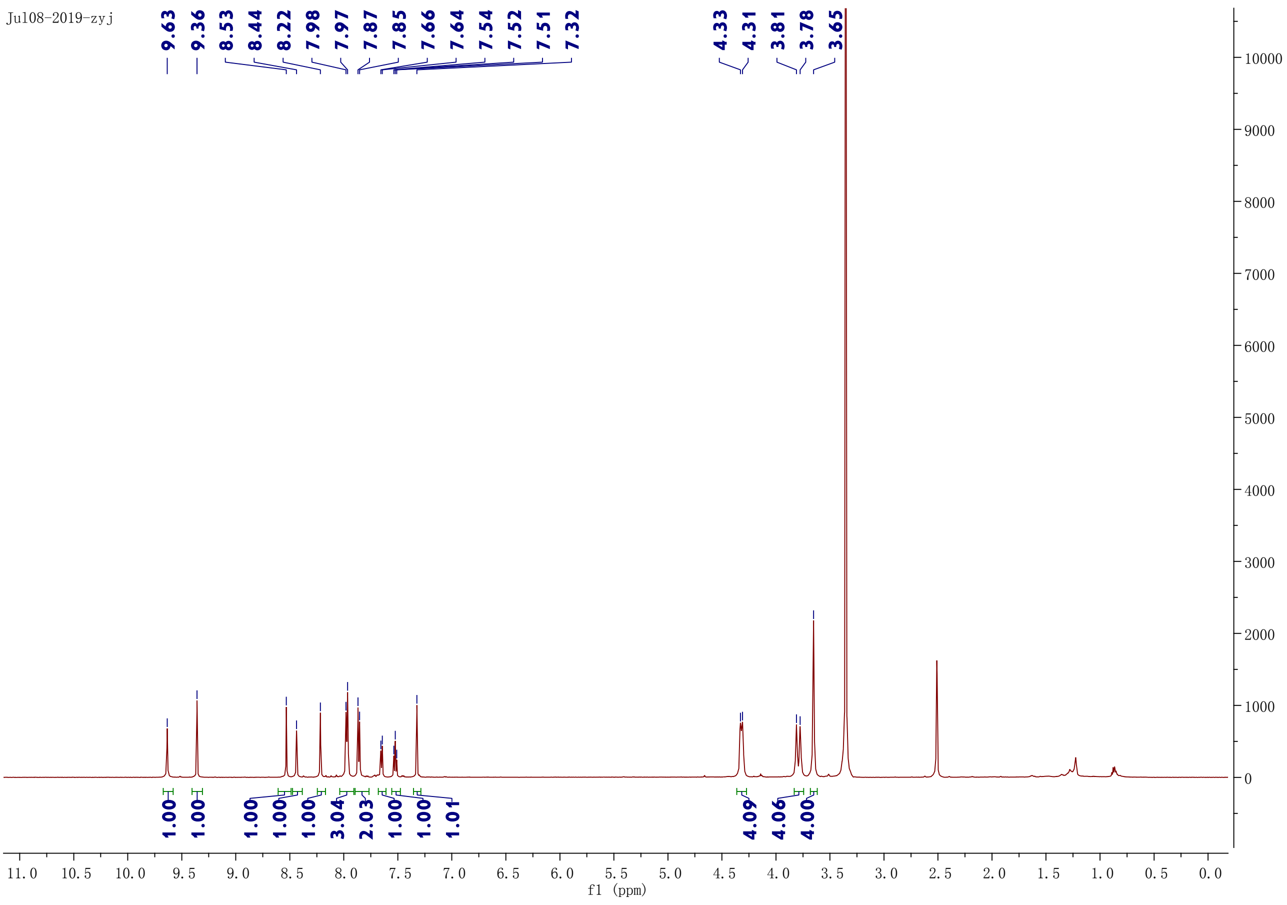

Figure S10-2. ^{13}C NMR spectrum (150MHz, DMSO-d6) of compound a10

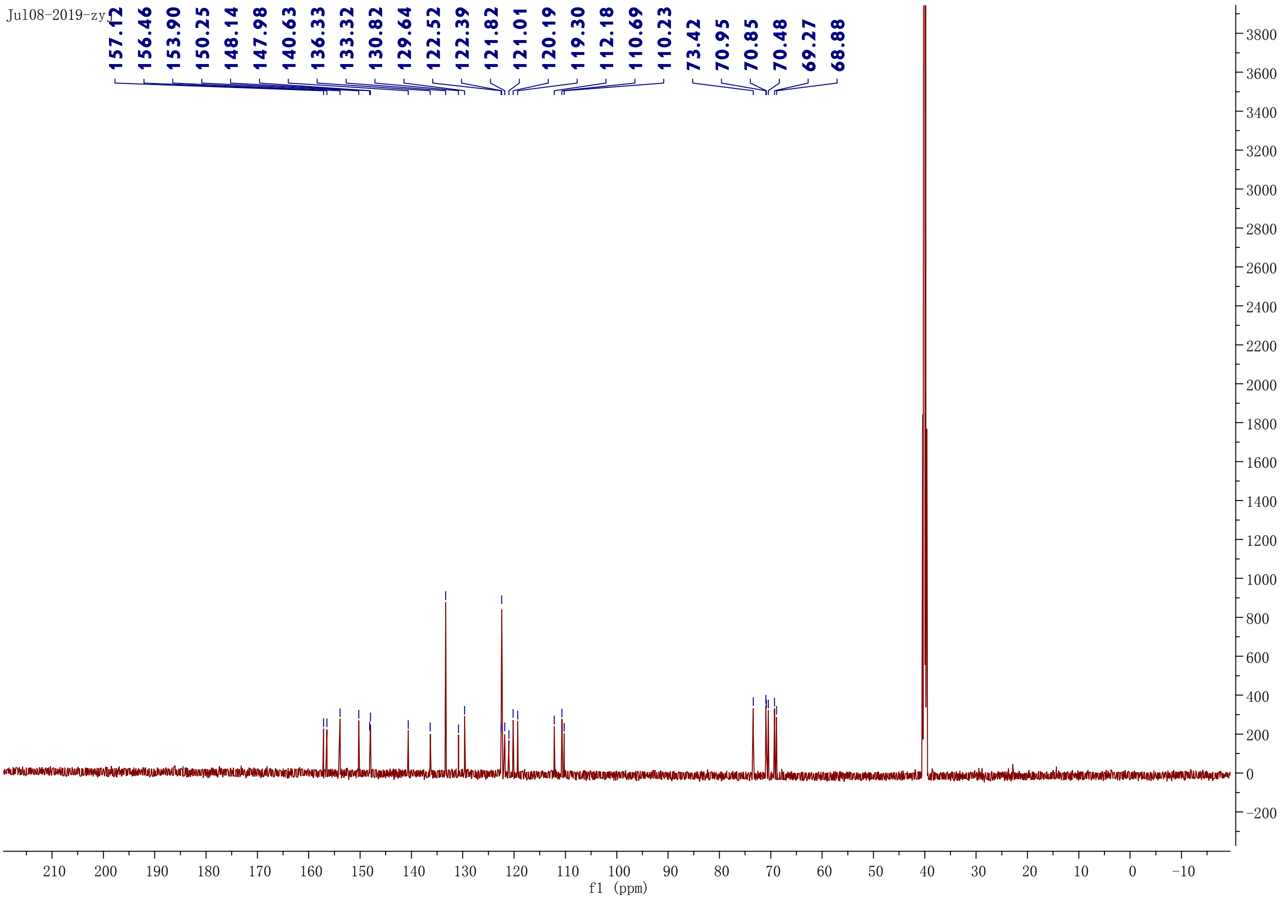

Figure S10-3. HR MS of compound a10

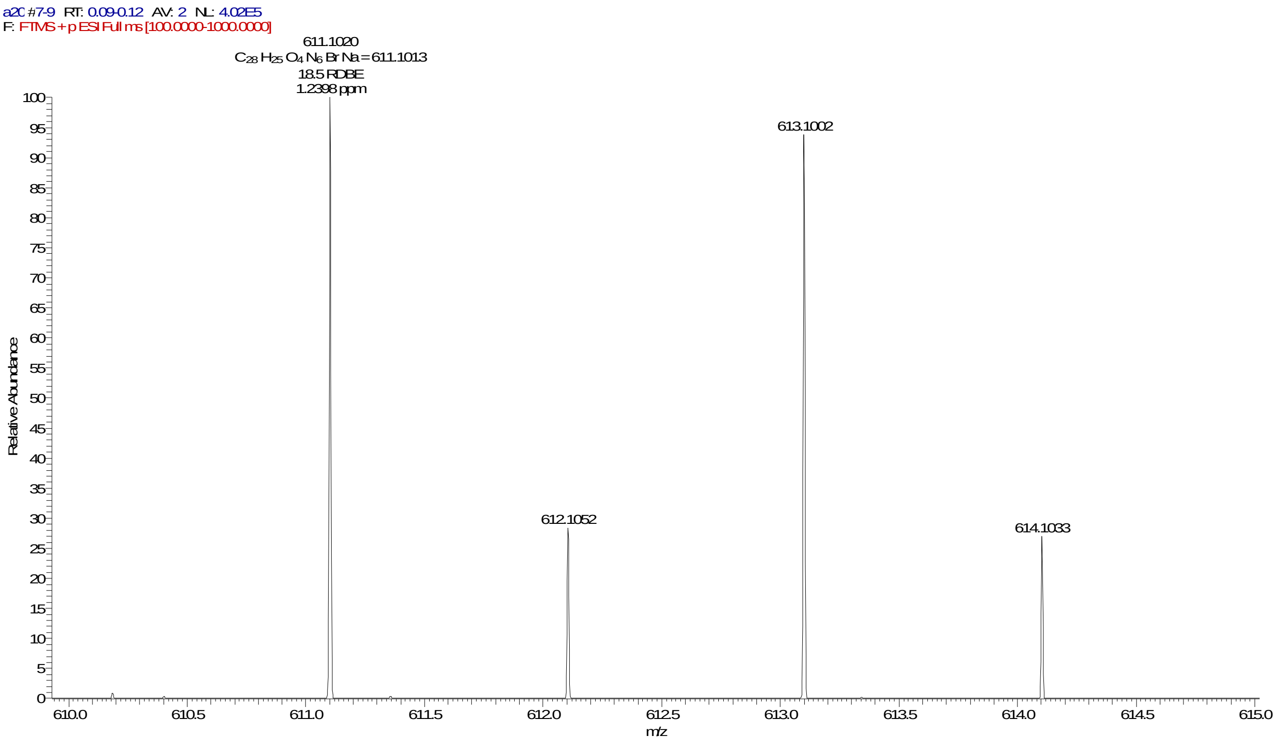

Figure S11-1. ^{1}H NMR spectrum (600MHz, DMSO-d6) of compound a11

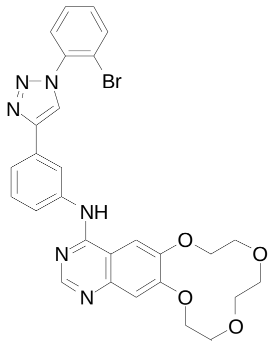

Chemical Formula: $C_{28}H_{25}BrN_6O_4$
Exact Mass: 588.11

a11

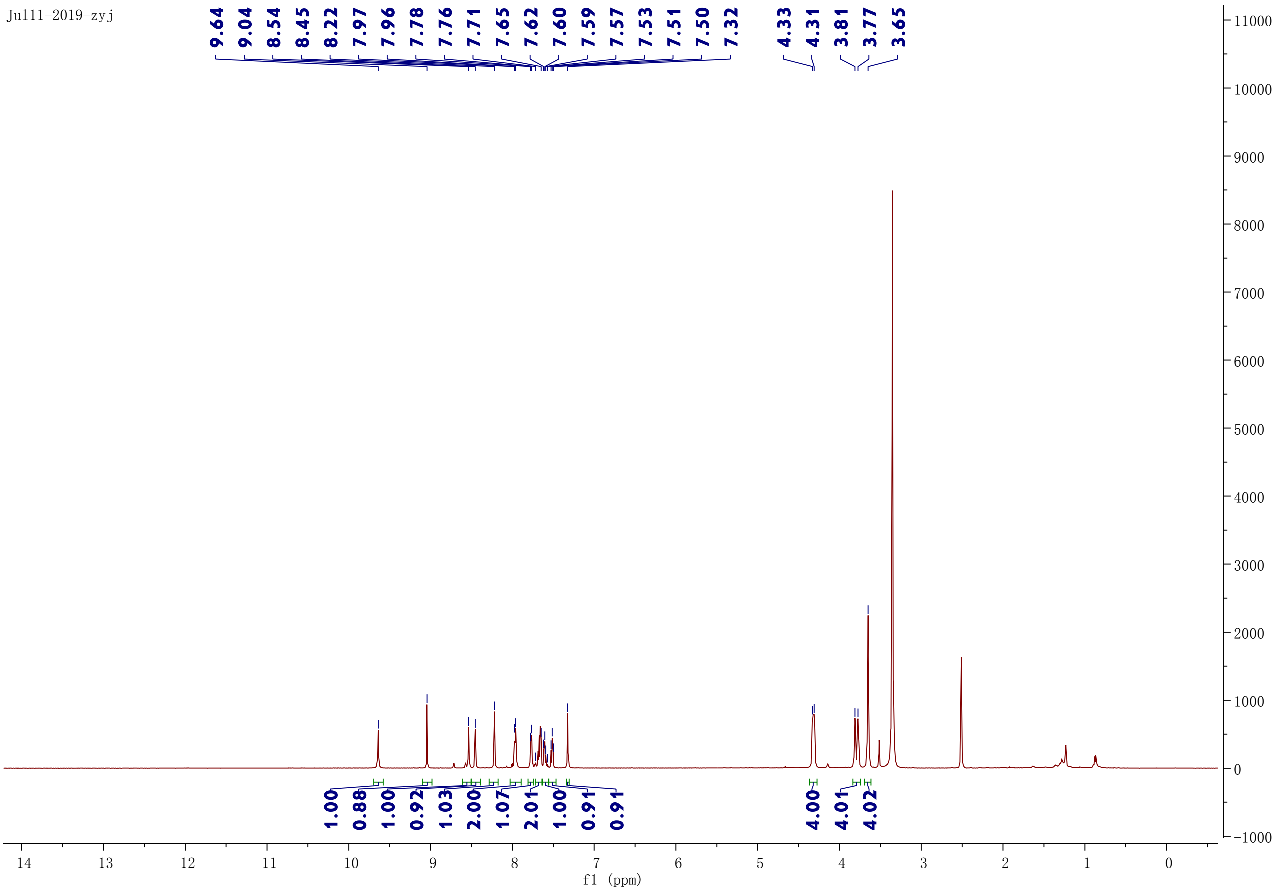

Figure S11-2. ^{13}C NMR spectrum (150MHz, DMSO-d6) of compound a11

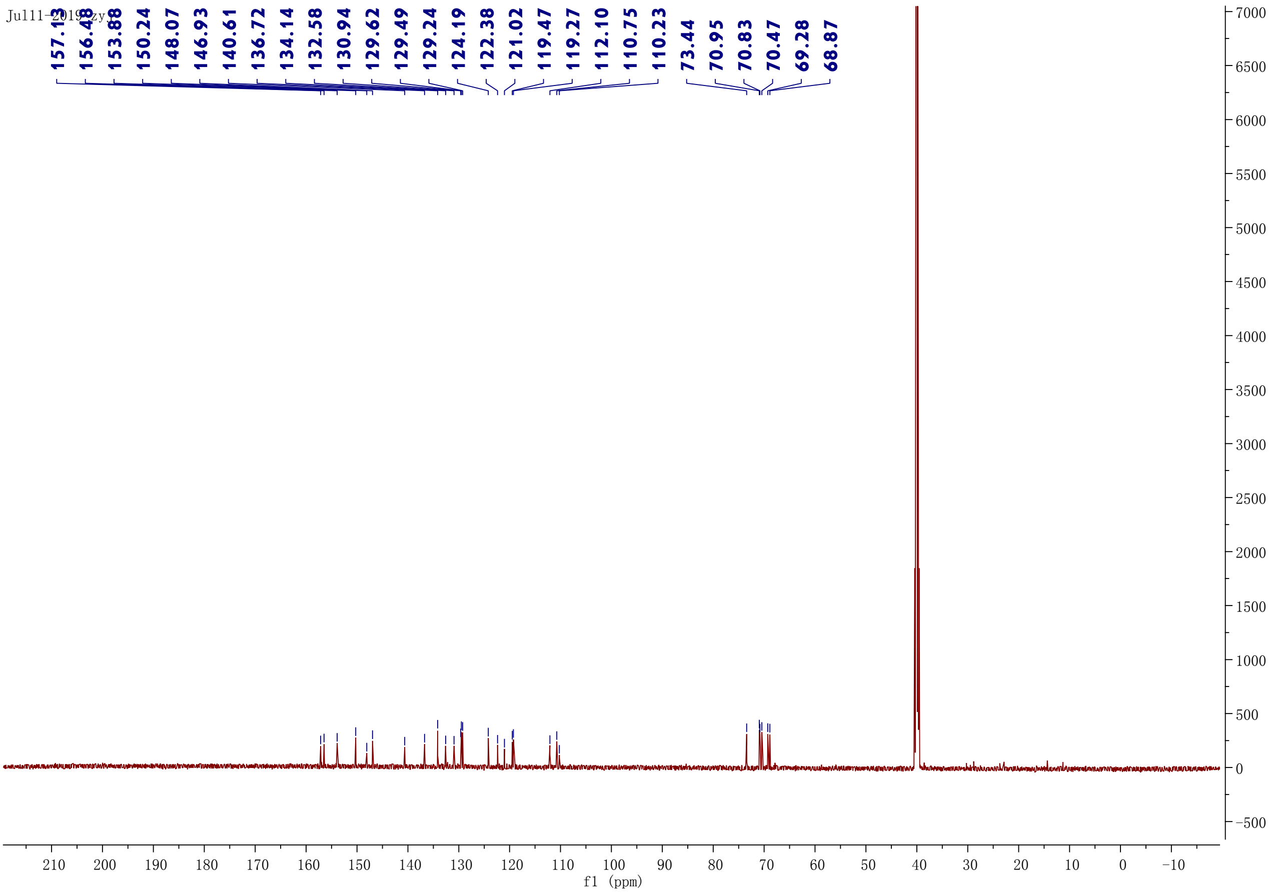

Figure S11-3. HR MS of compound a11

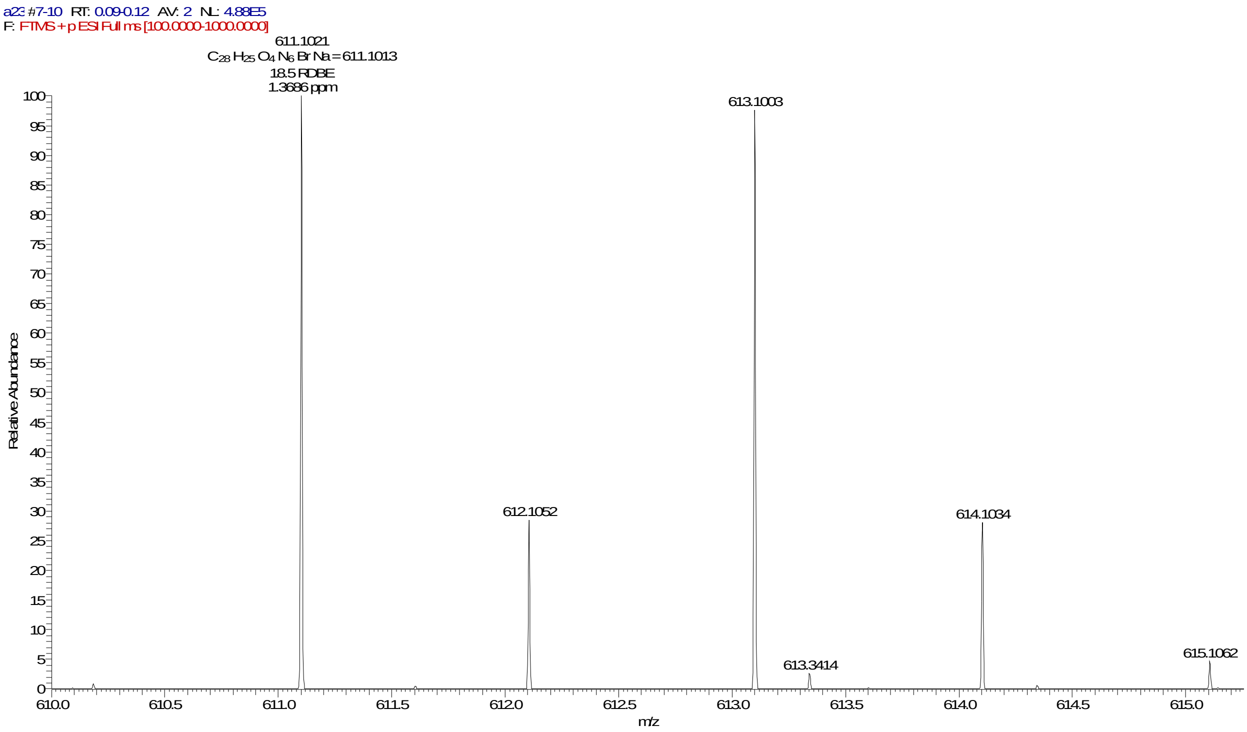

Figure S12-1. ^{1}H NMR spectrum (600MHz, DMSO-d6) of compound a12

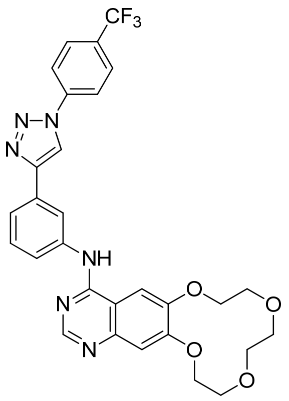

Chemical Formula: $C_{29}H_{25}F_3N_6O_4$
Exact Mass: 578.19

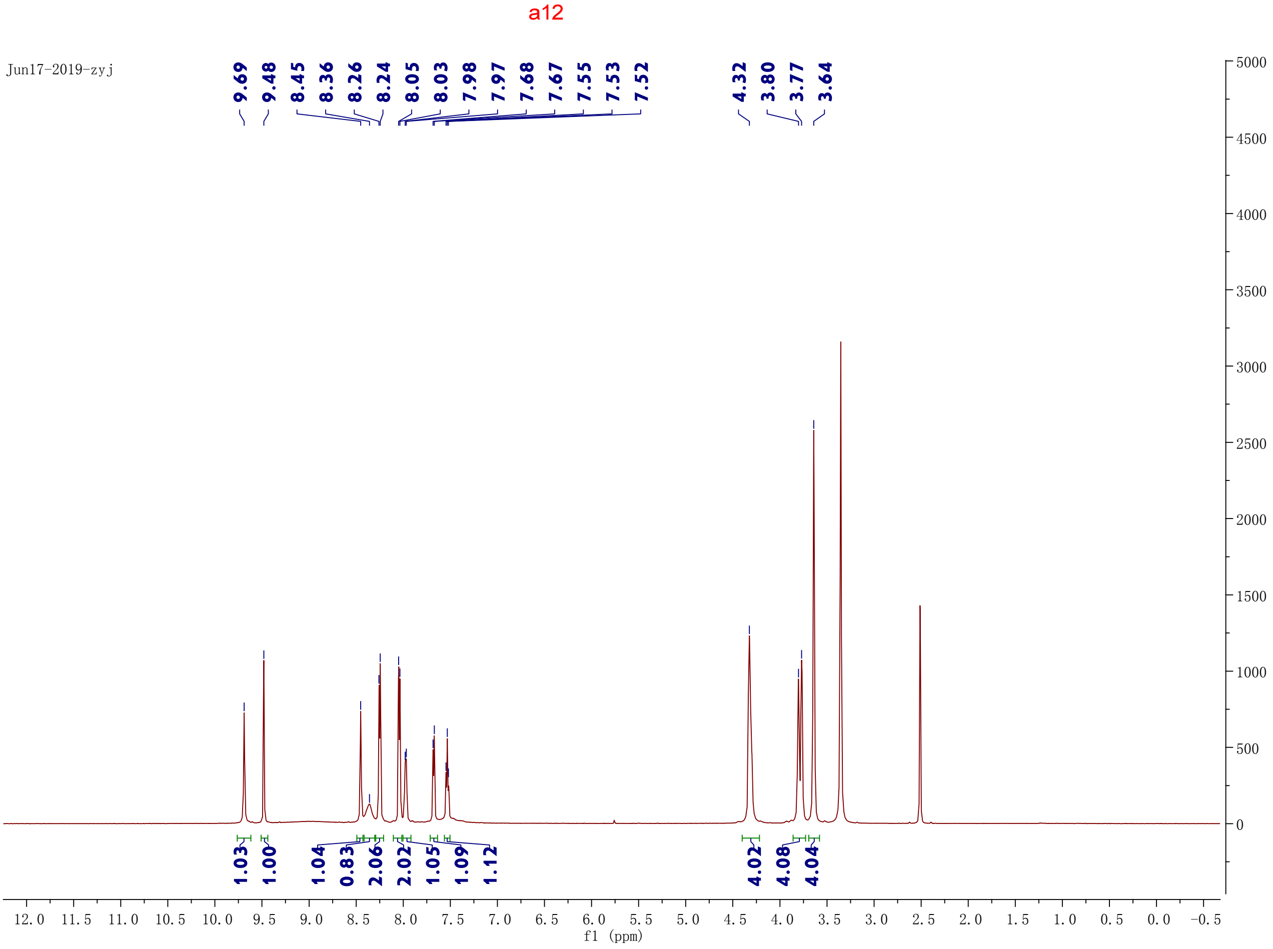

Figure S12-2. [12]C NMR spectrum (150MHz, DMSO-d6) of compound a12

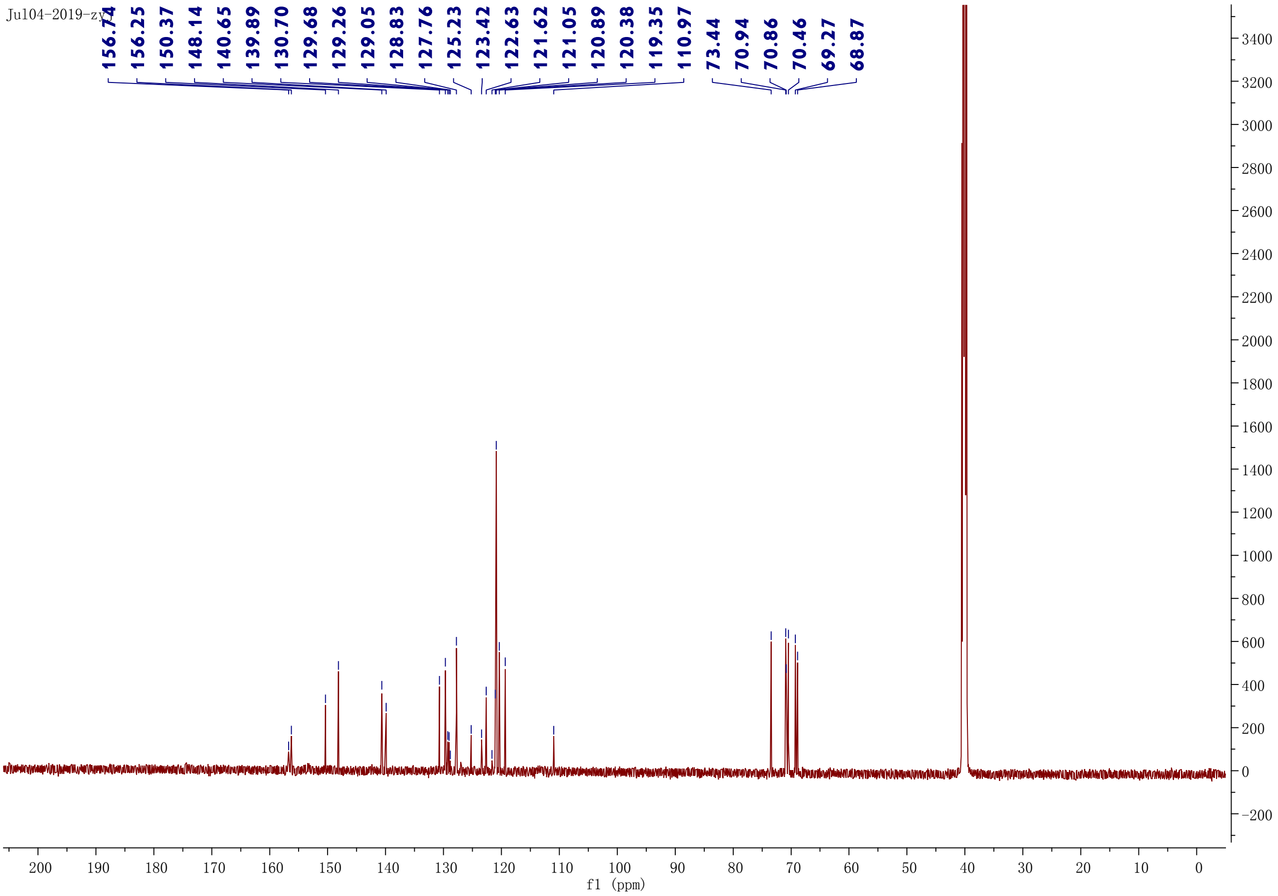

Figure S12-3. HR MS of compound a12

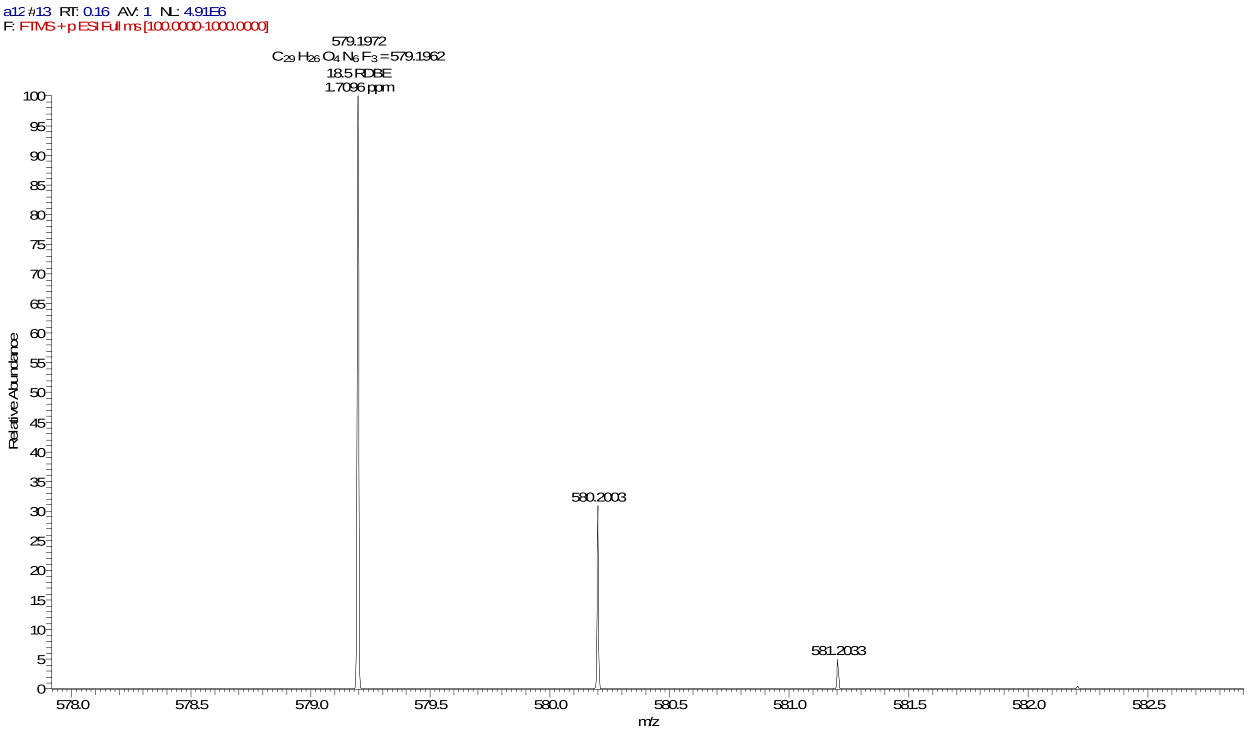

Figure S13-1. ^{1}H NMR spectrum (600MHz, DMSO-d6) of compound a13

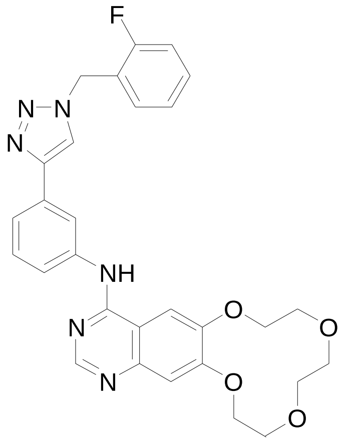

a13

Chemical Formula: $C_{29}H_{27}FN_6O_4$
Exact Mass: 542.21

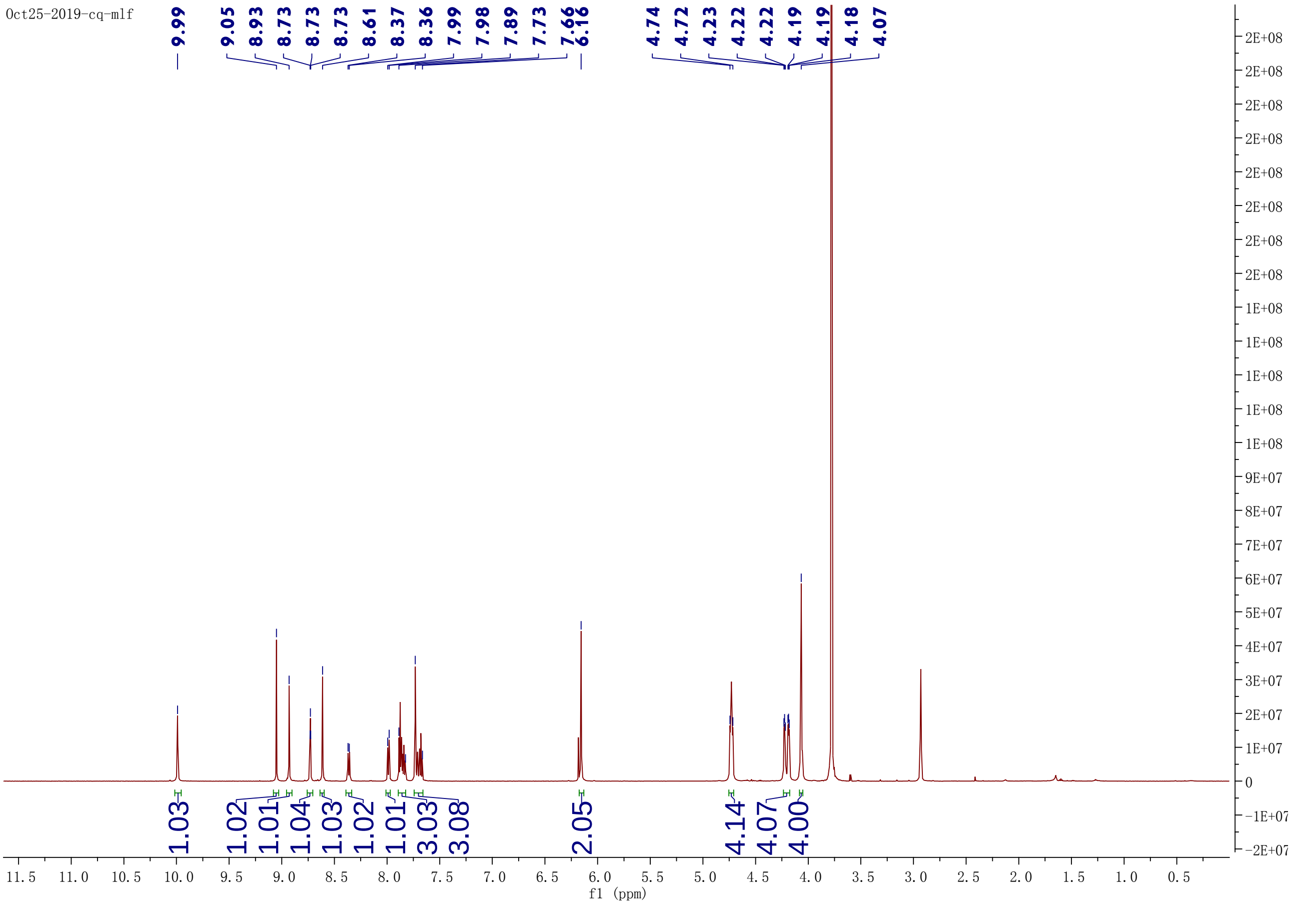

Figure S13-2. ^{13}C NMR spectrum (150MHz, DMSO-d6) of compound a13

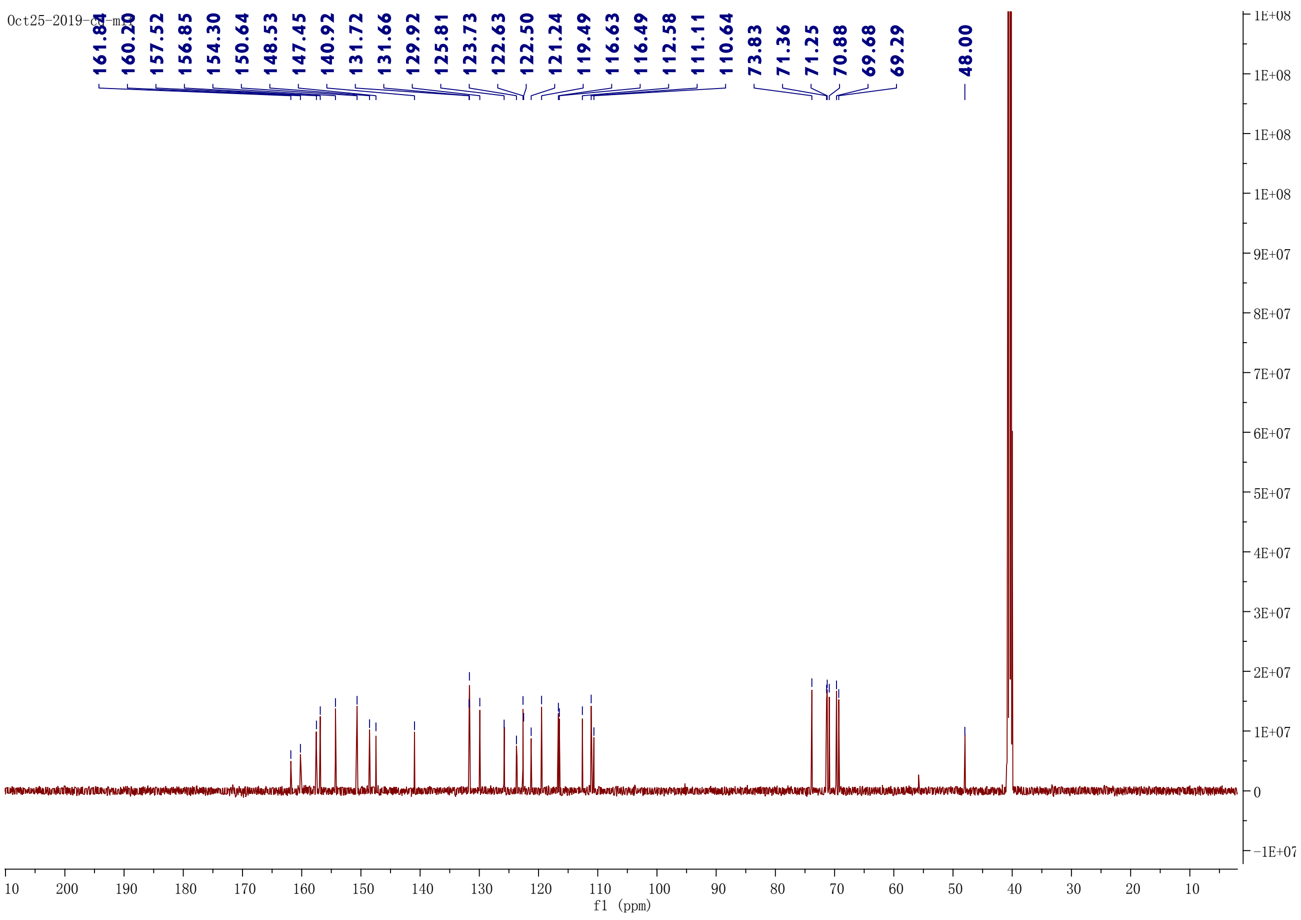

Figure S13-3. HR MS of compound a13

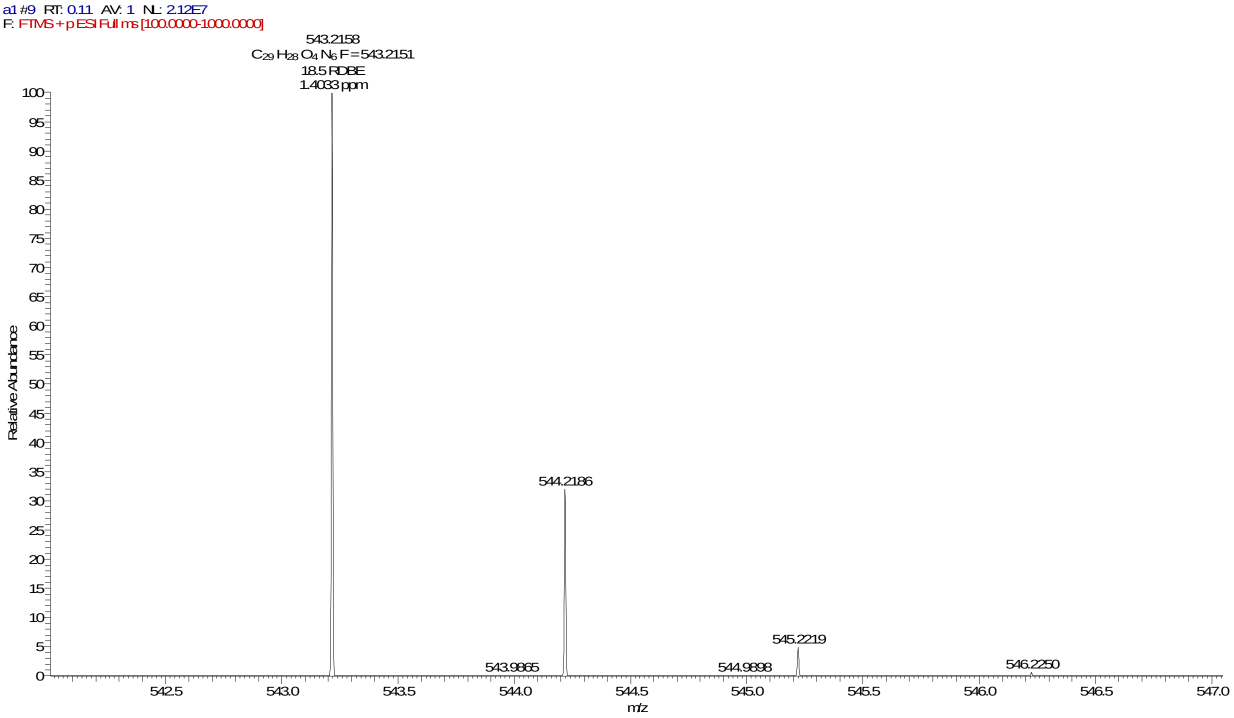

Figure S14-1. ^{1}H NMR spectrum (600MHz, DMSO-d6) of compound a14

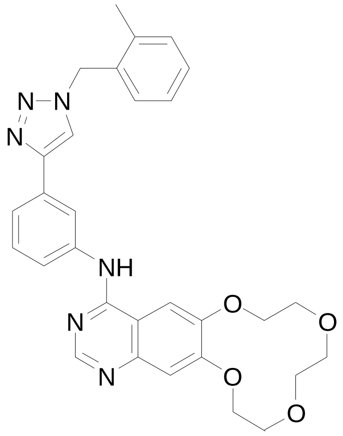

a14

Chemical Formula: $C_{30}H_{30}N_6O_4$
Exact Mass: 538.23

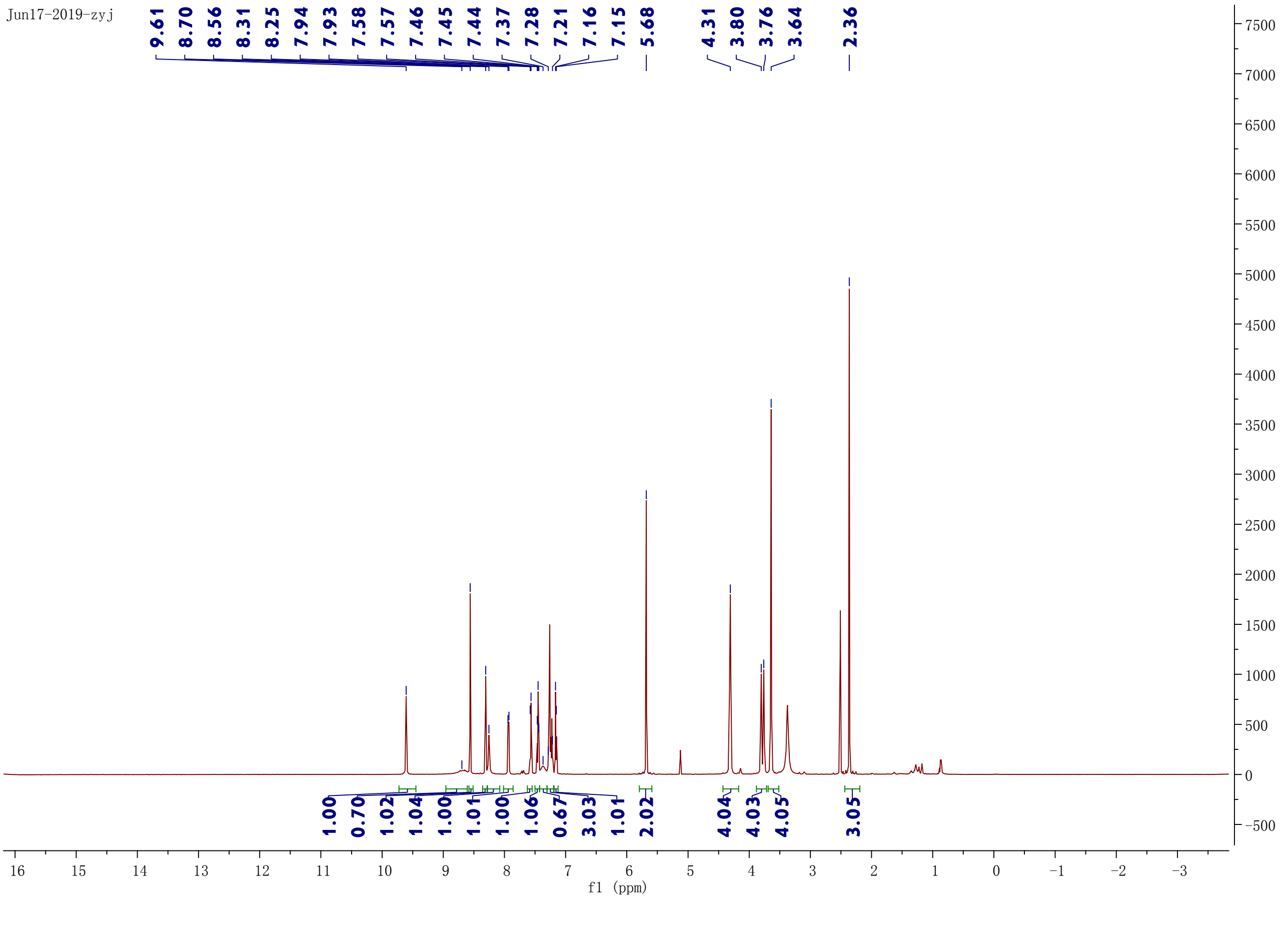

Figure S14-2. ^{13}C NMR spectrum (150MHz, DMSO-d6) of compound a14

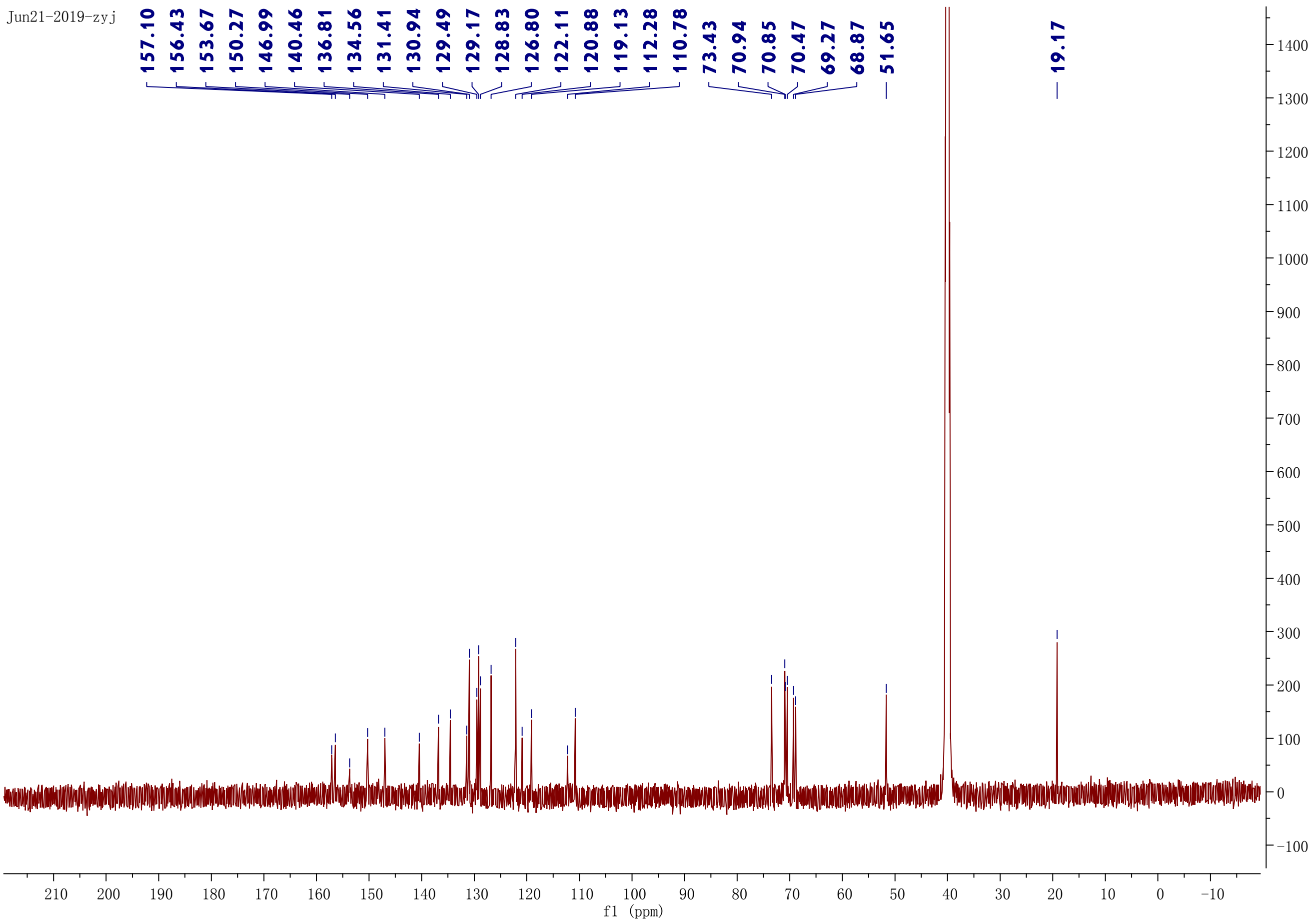

Figure S14-3. HR MS of compound a14

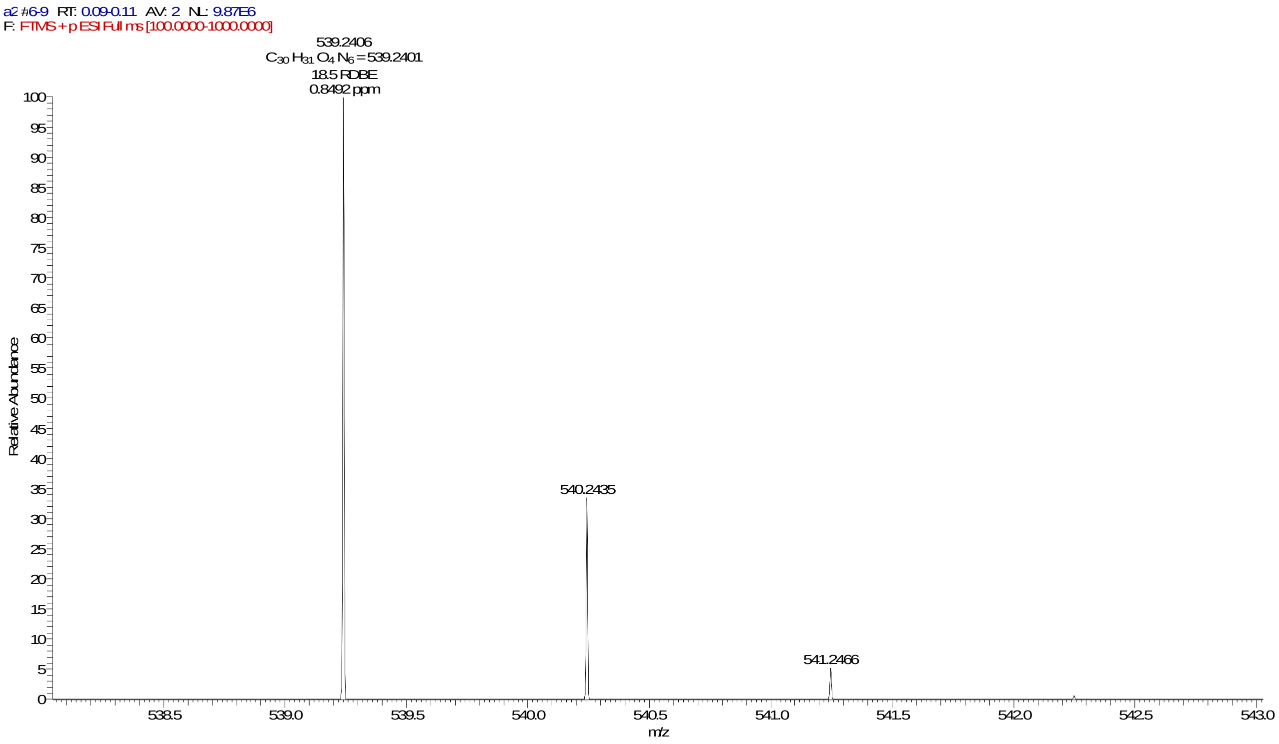

Figure S15-1. ^{1}H NMR spectrum (600MHz, DMSO-d6) of compound a15

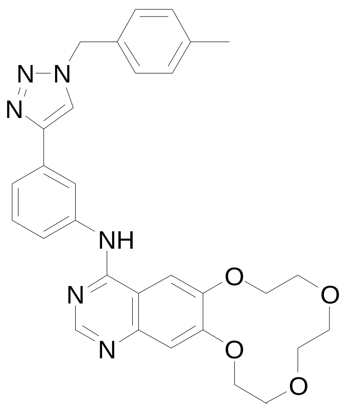

a15

Chemical Formula: $C_{30}H_{30}N_6O_4$
Exact Mass: 538.23

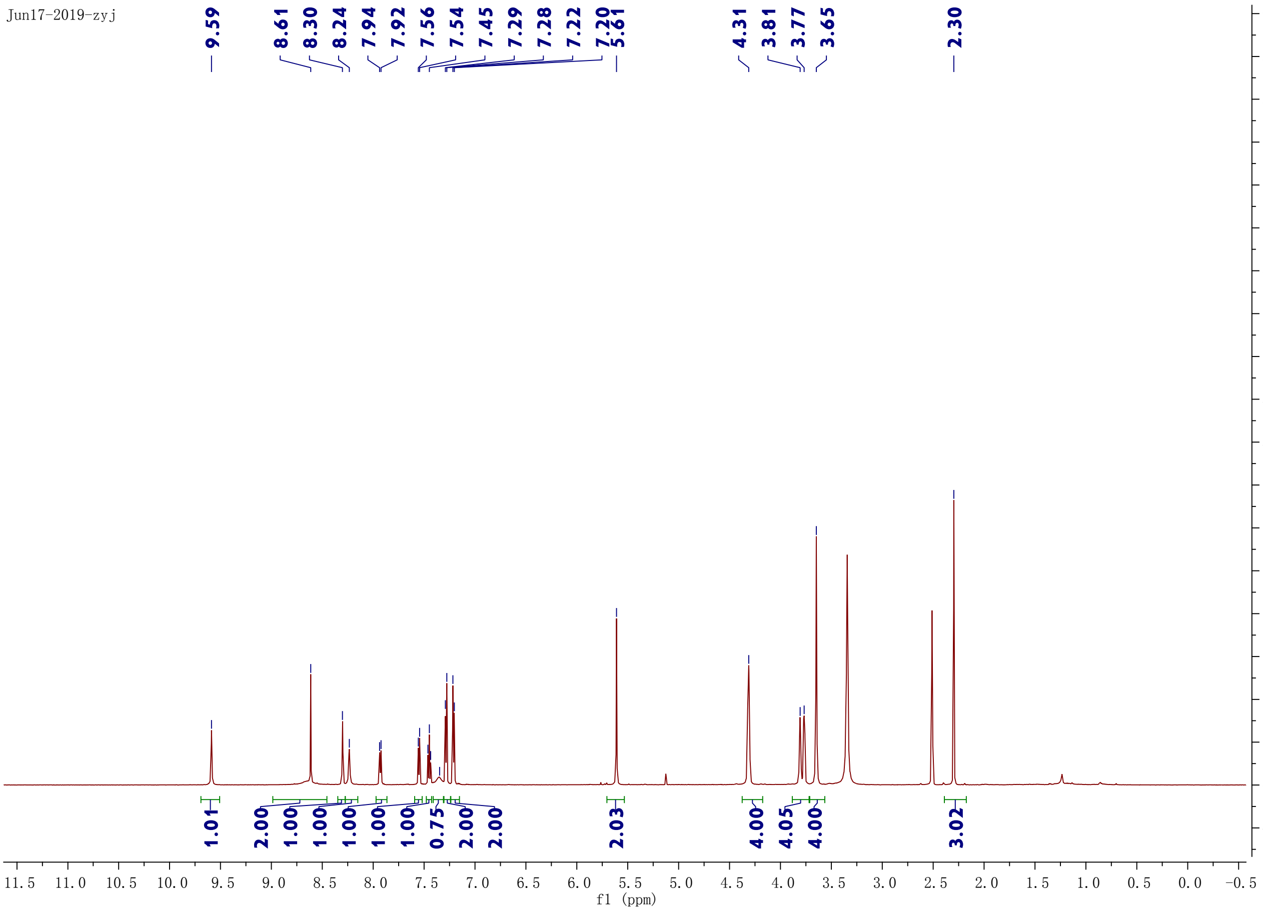

Figure S15-2. ^{13}C NMR spectrum (150MHz, DMSO-d6) of compound a15

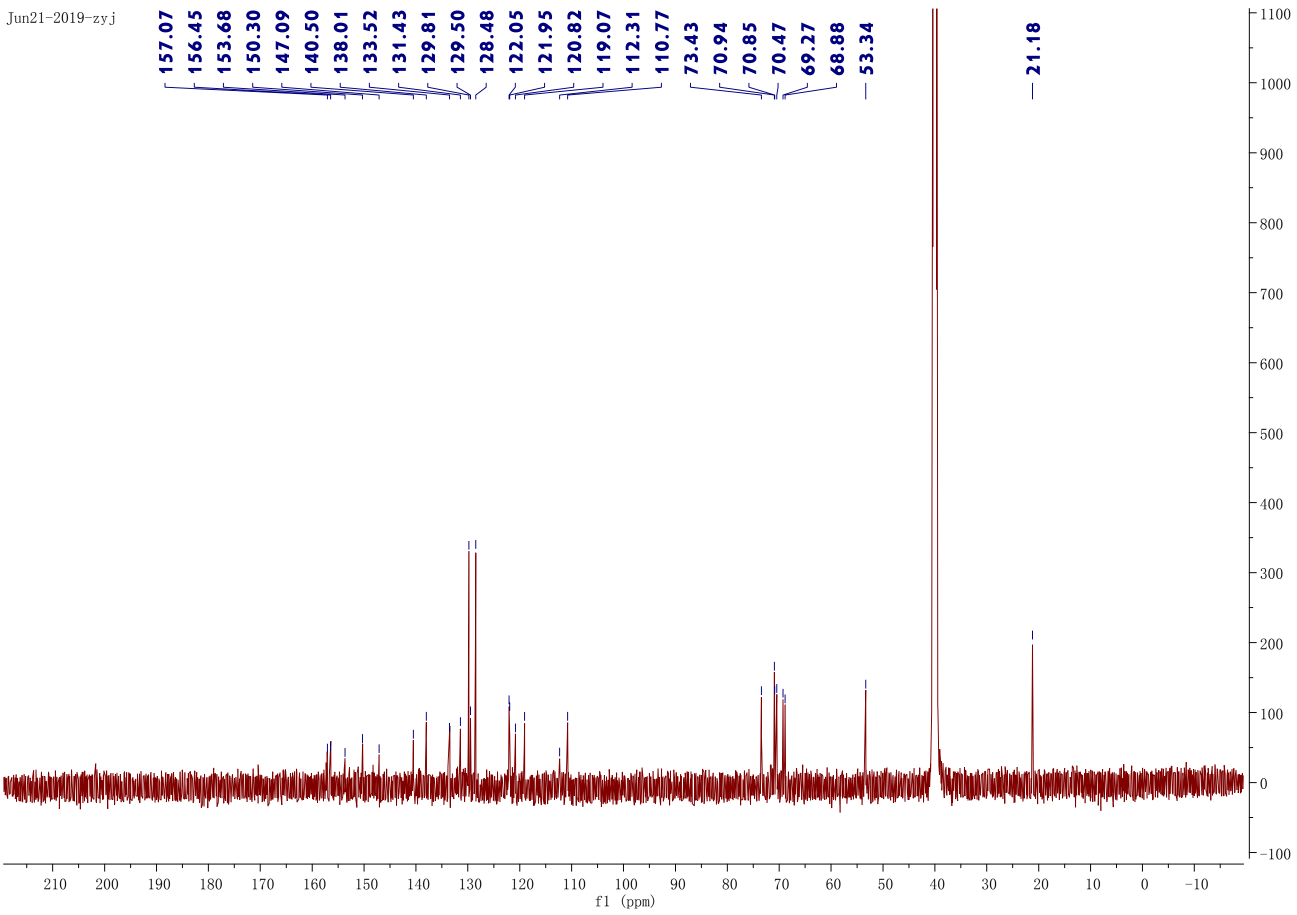

Figure S15-3. HR MS of compound a15

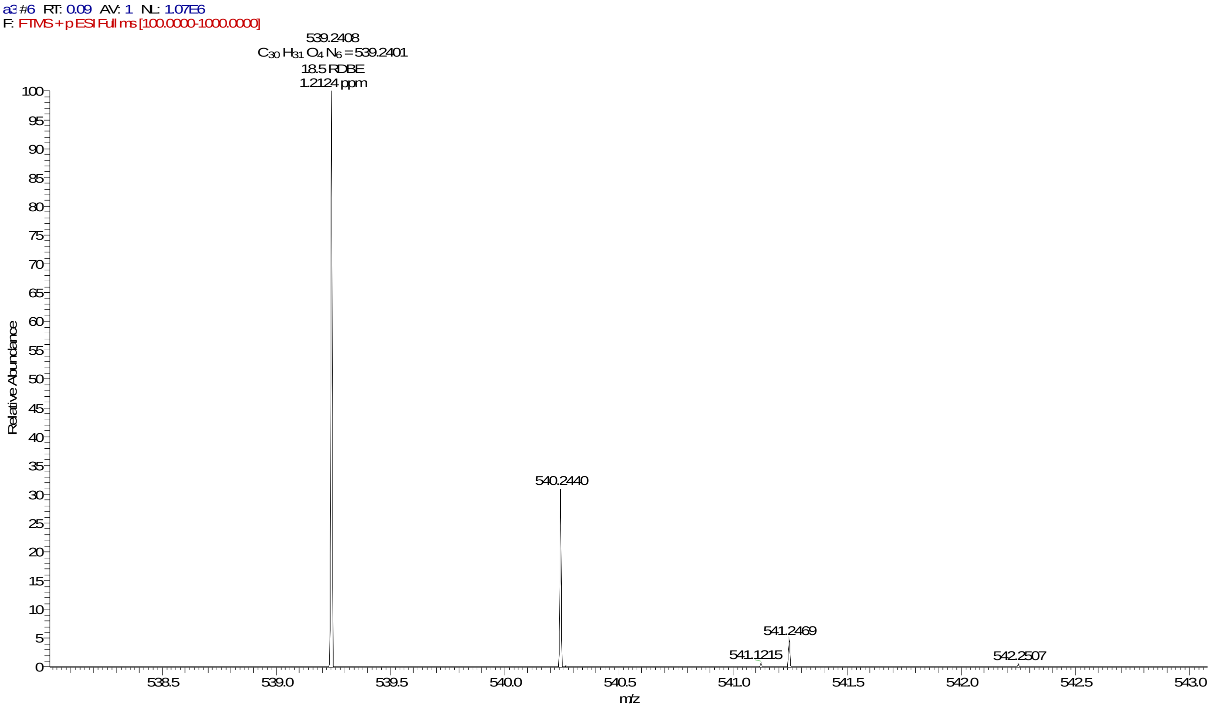

Figure S16-1. ^{1}H NMR spectrum (600MHz, DMSO-d6) of compound a16

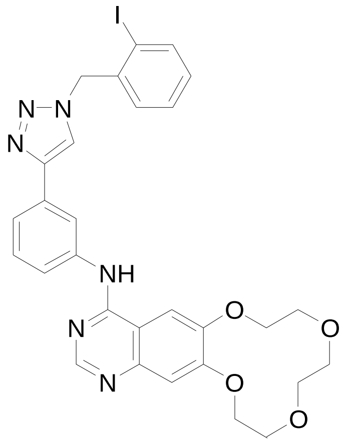

a16

Chemical Formula: $C_{29}H_{27}IN_6O_4$
Exact Mass: 650.11

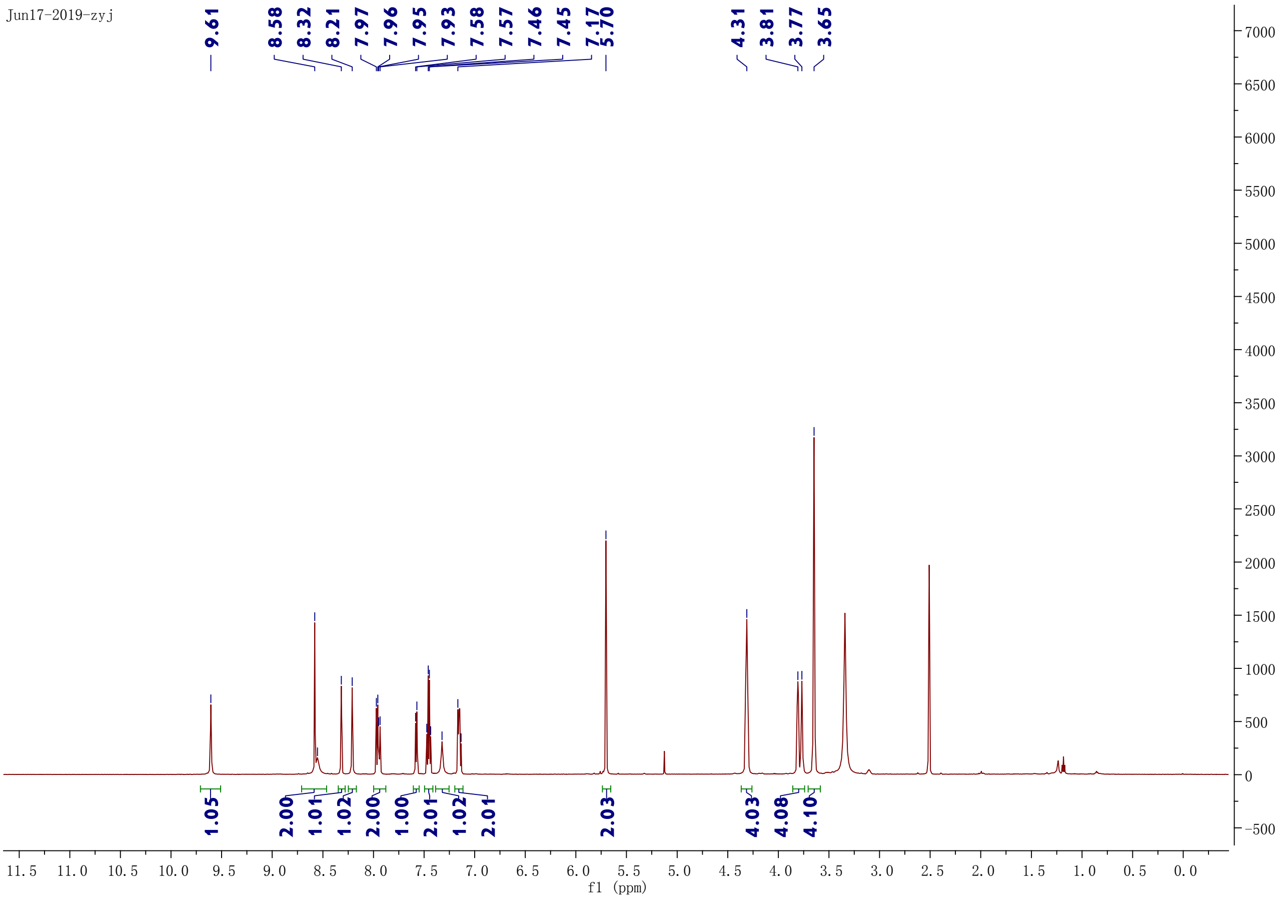

Figure S16-2. ^{13}C NMR spectrum (150MHz, DMSO-d6) of compound a16

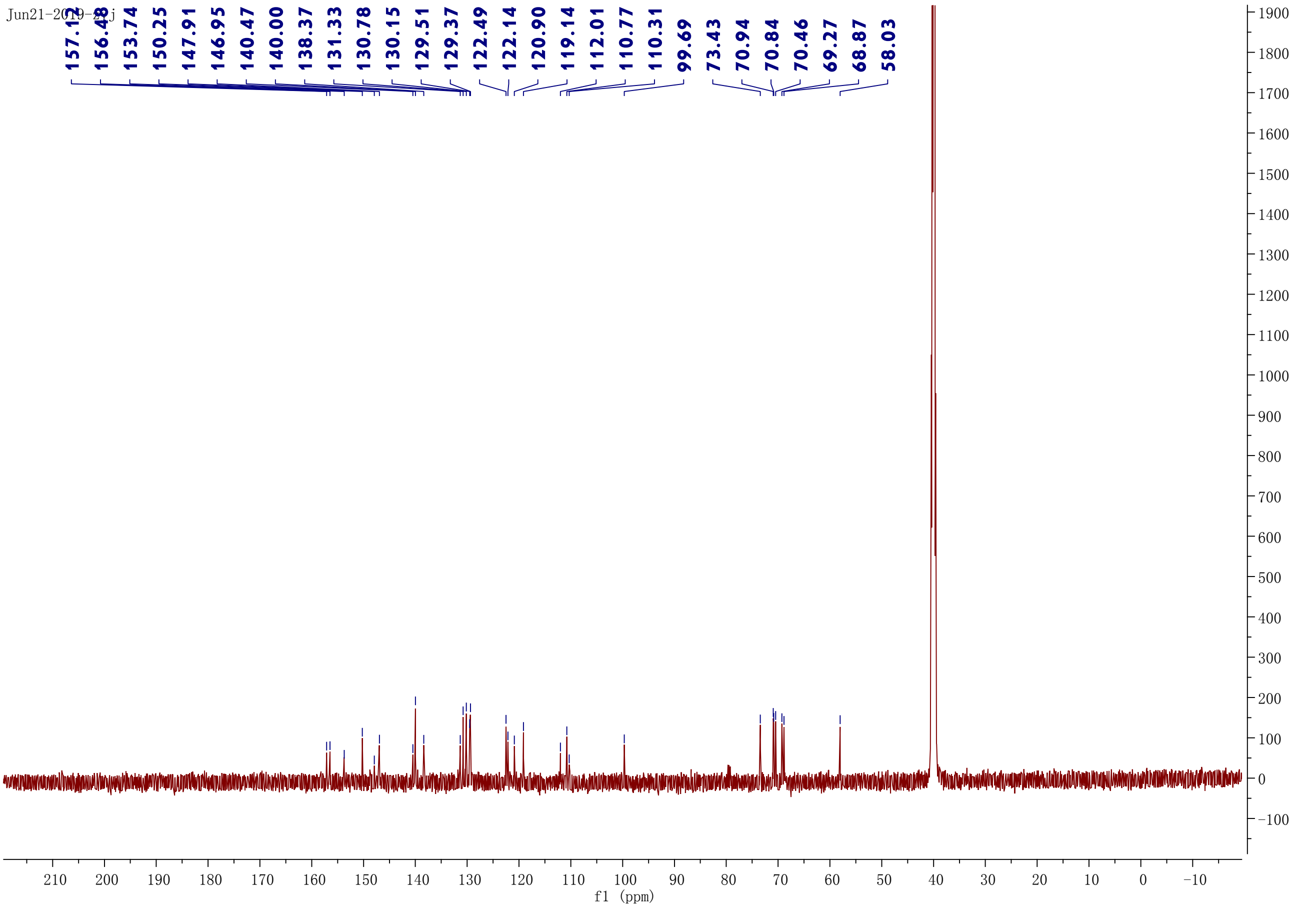

Figure S16-3. HR MS of compound a16

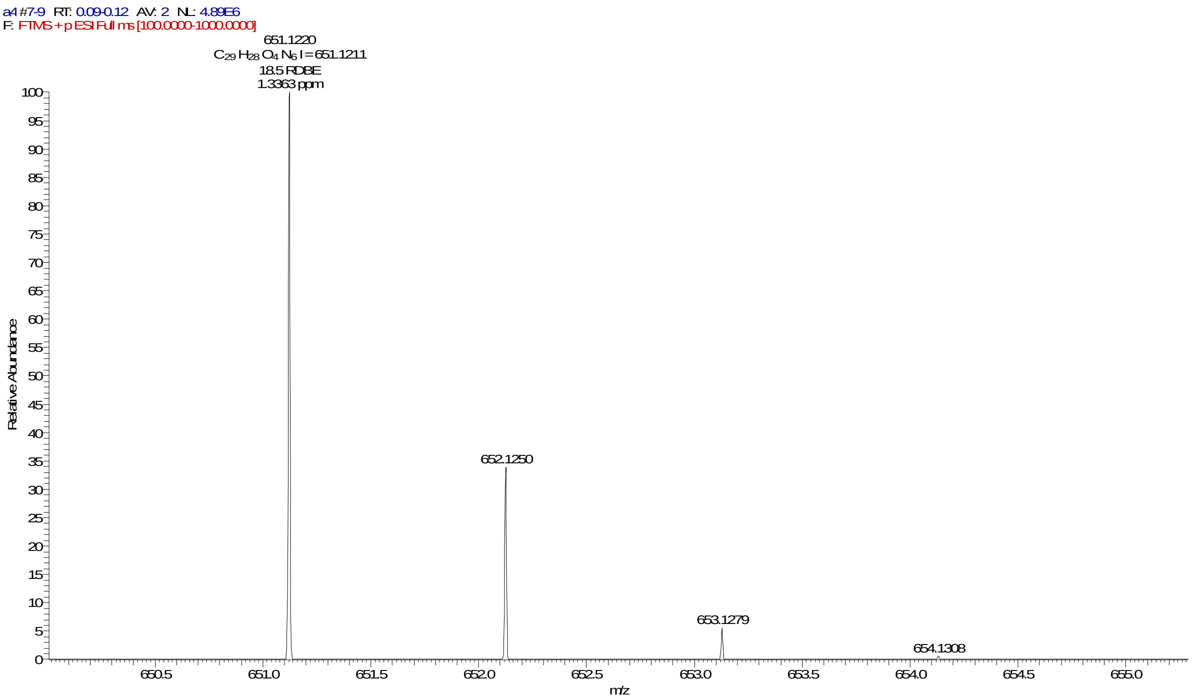

Figure S17-1. ^{1}H NMR spectrum (600MHz, DMSO-d6) of compound a17

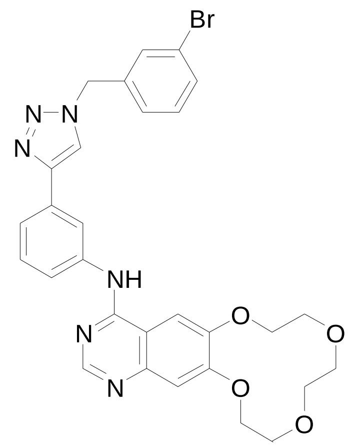

a17

Chemical Formula: $C_{29}H_{27}BrN_6O_4$
Exact Mass: 602.13

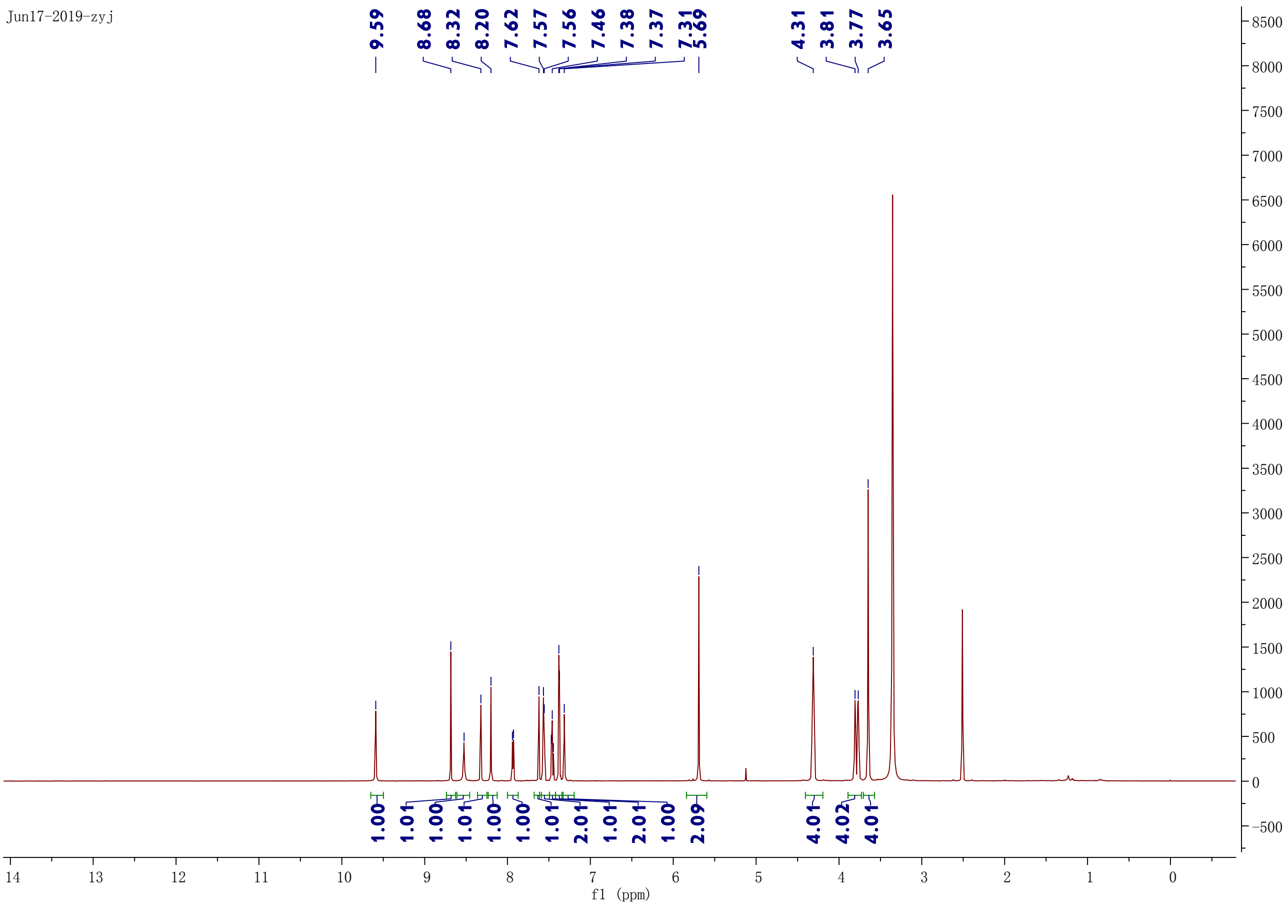

Figure S17-2. ^{13}C NMR spectrum (150MHz, DMSO-d6) of compound a17

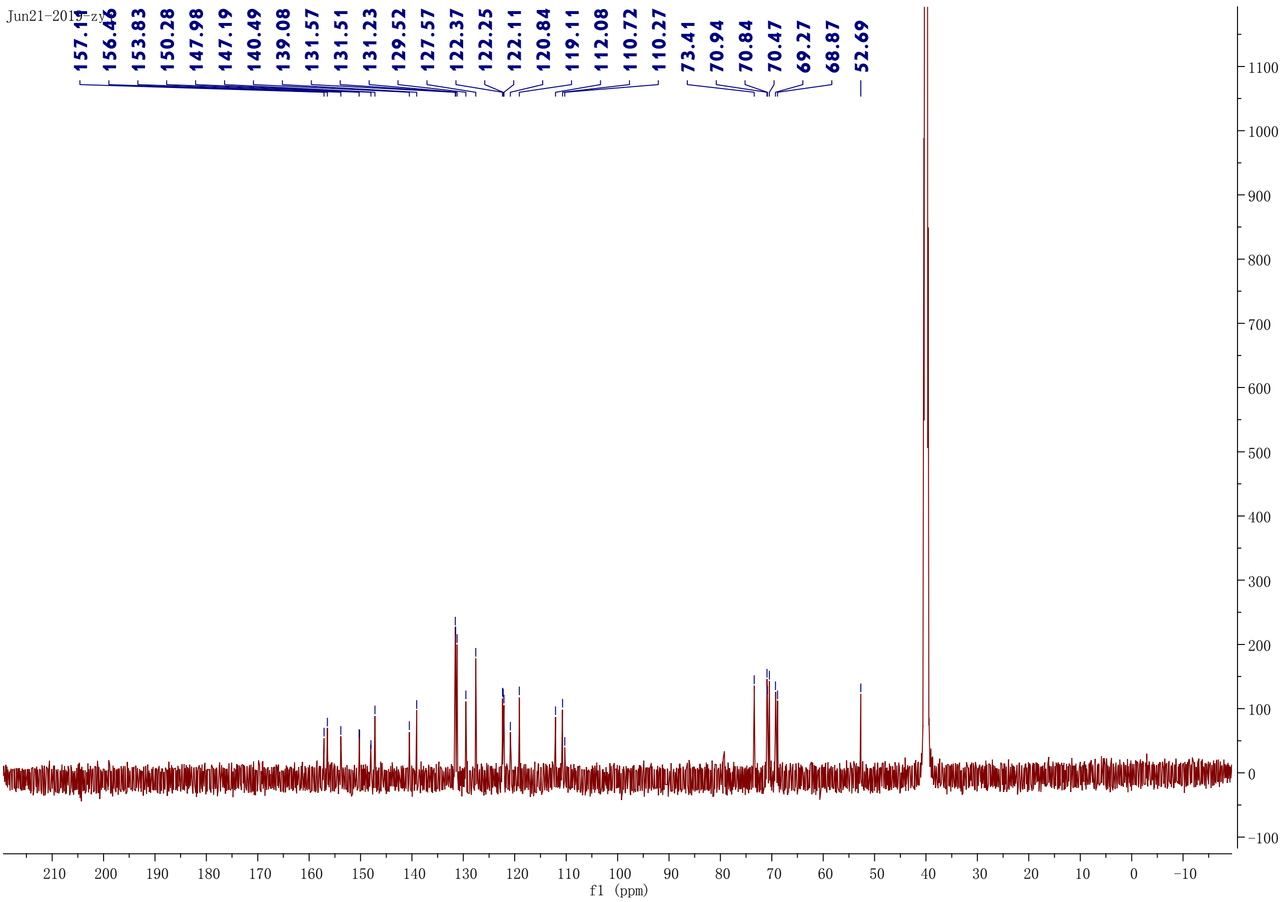

Figure S17-3. HR MS of compound a17

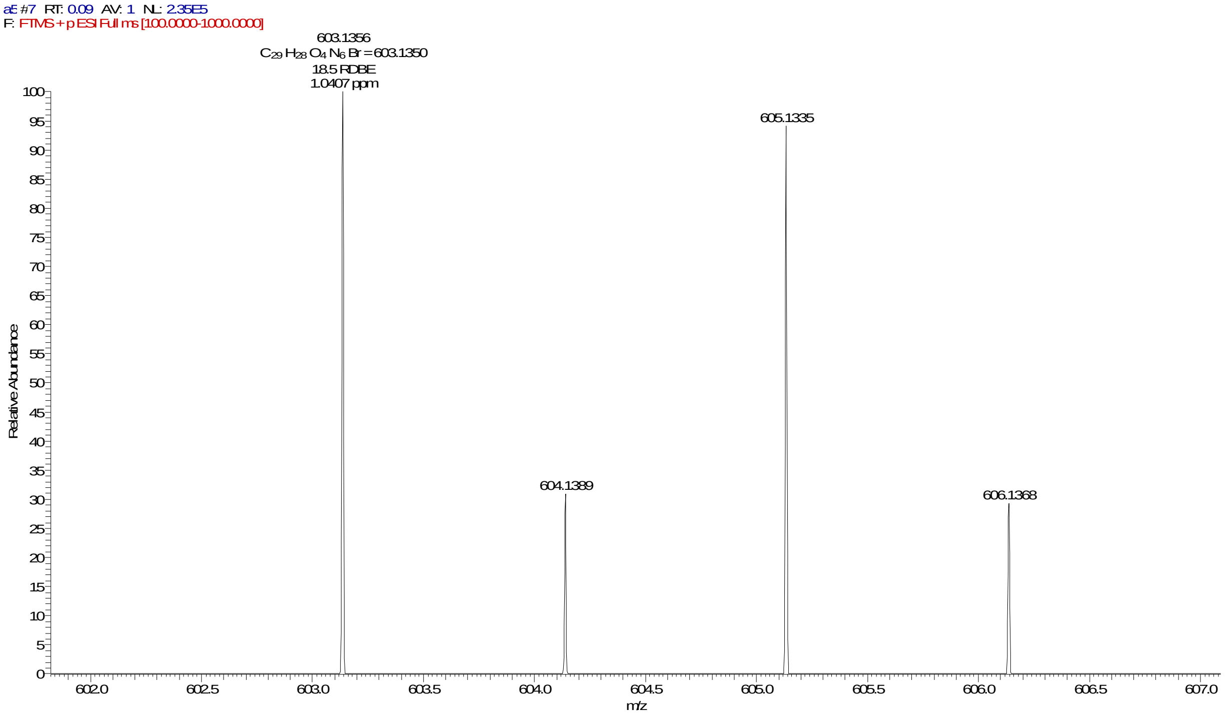

Figure S18-1. ^{1}H NMR spectrum (600MHz, DMSO-d6) of compound a18

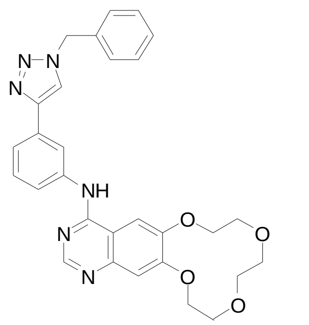

Chemical Formula: $C_{29}H_{28}N_6O_4$
Exact Mass: 524.22

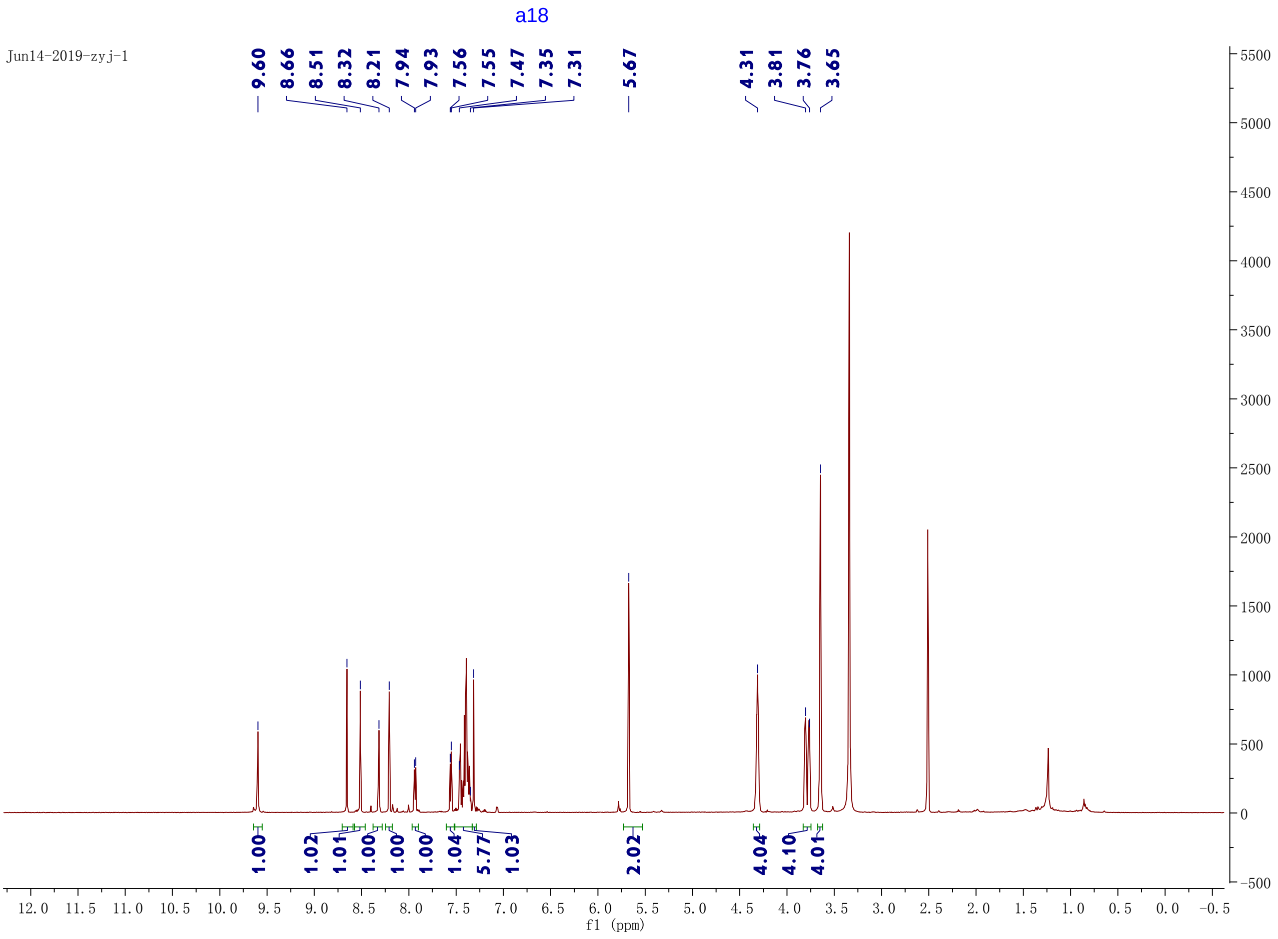

Figure S18-2. ^{13}C NMR spectrum (150MHz, DMSO-d6) of compound a18

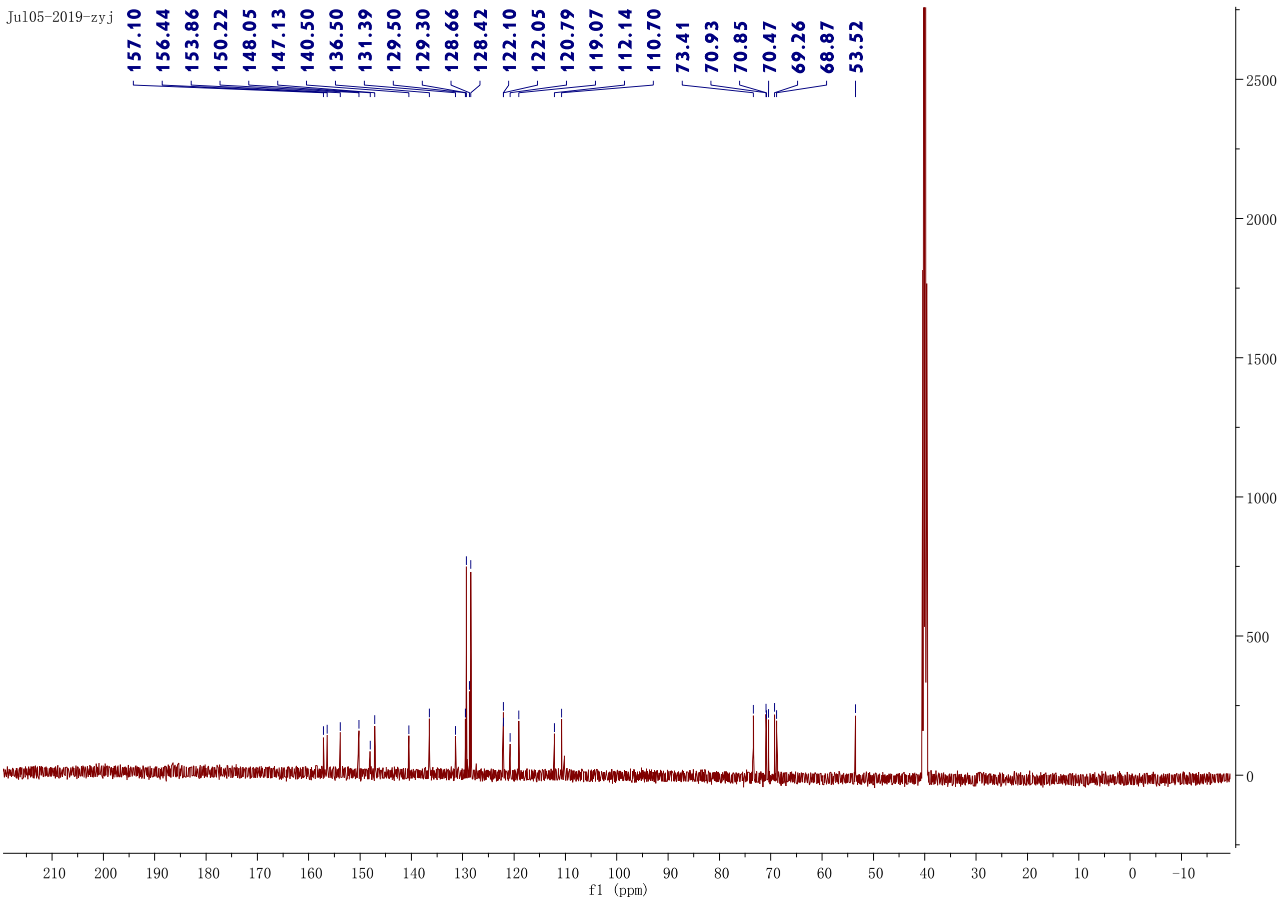

Figure S18-3. HR MS of compound a18

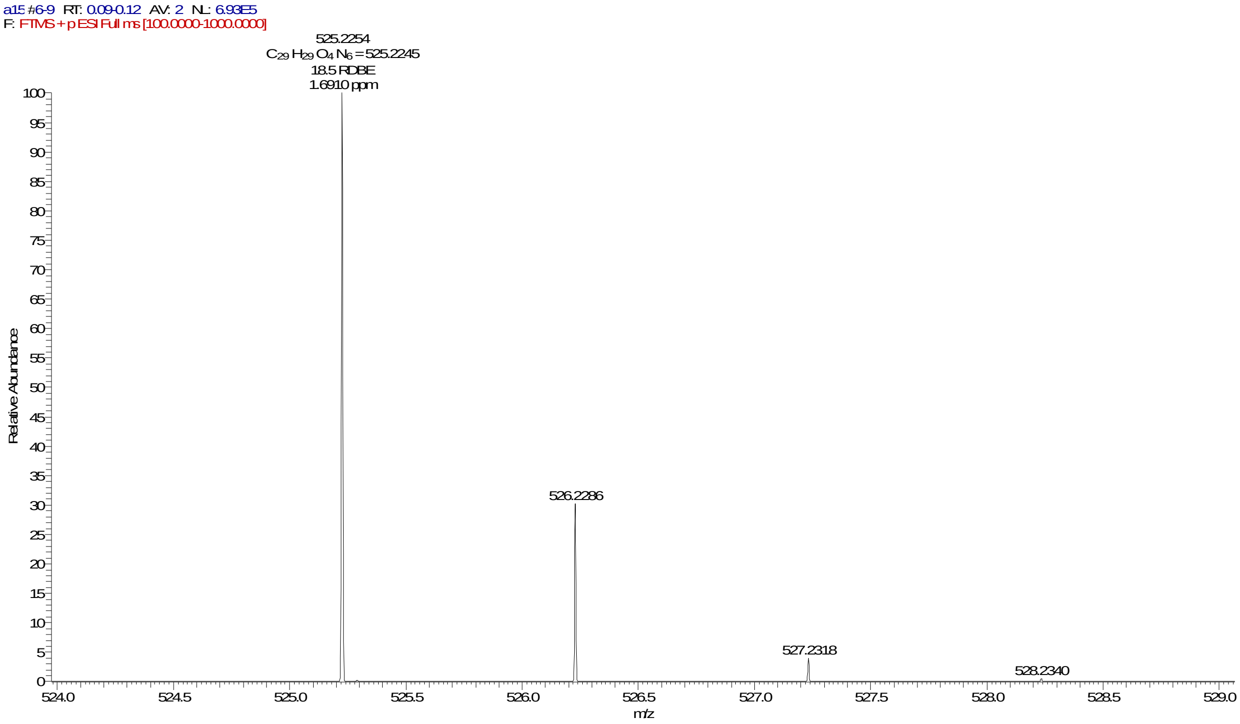

Figure S19-1. ^{1}H NMR spectrum (600MHz, DMSO-d6) of compound a19

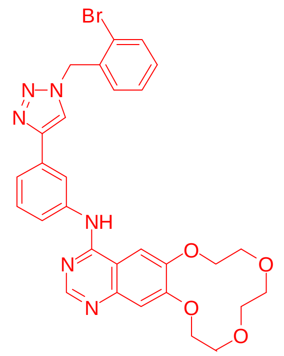

Chemical Formula: $C_{29}H_{27}BrN_6O_4$
Exact Mass: 602.13

a19

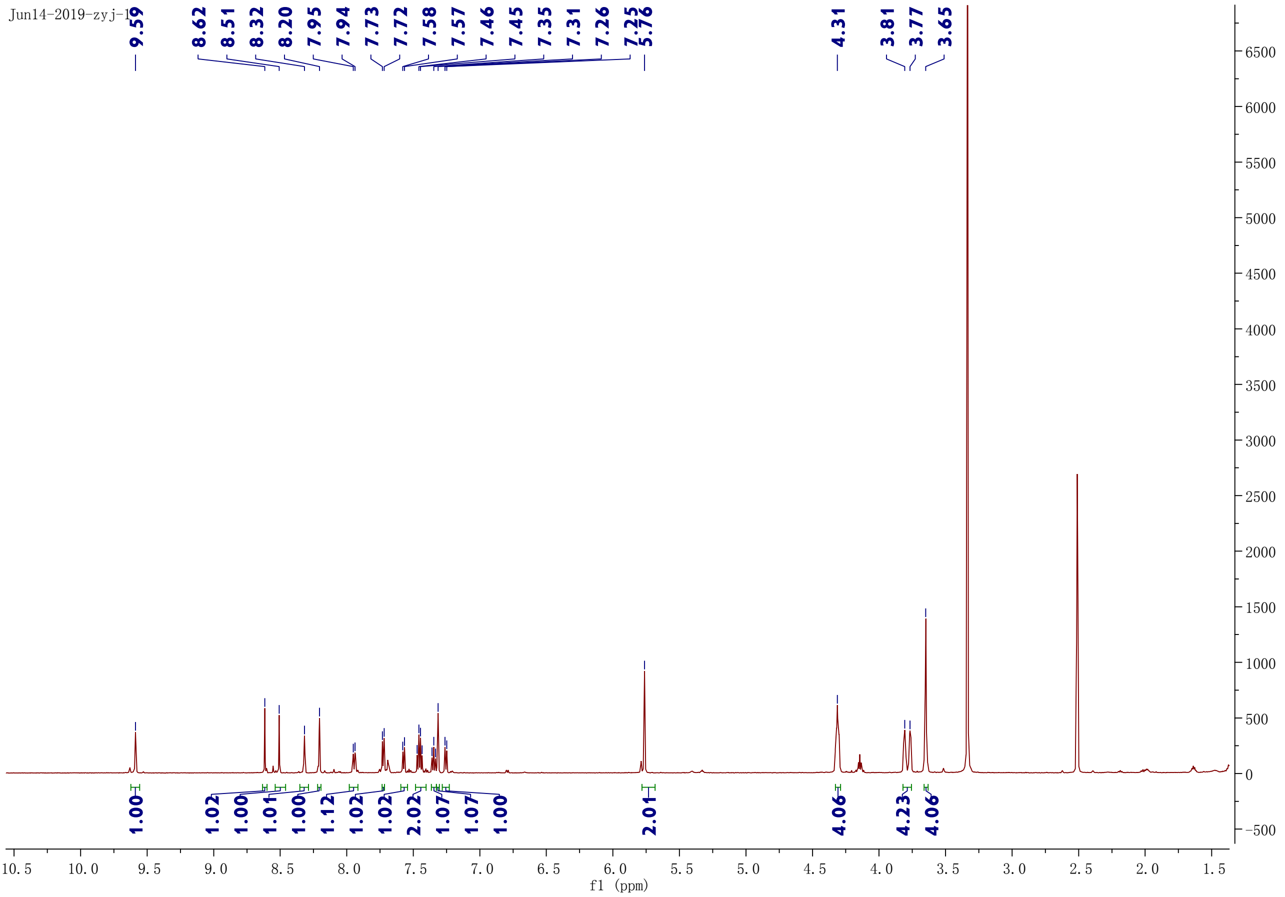

Figure S19-2. HR MS of compound a19

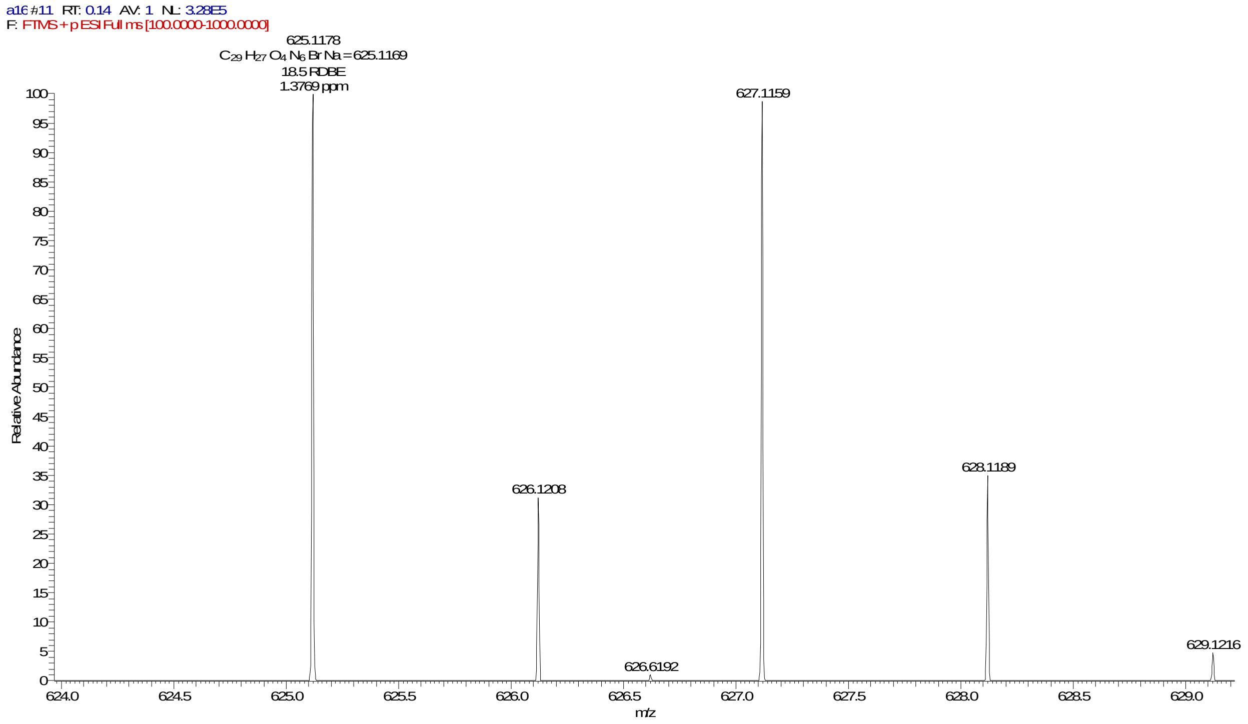

Figure S20-1. ^{1}H NMR spectrum (600MHz, DMSO-d6) of compound a20

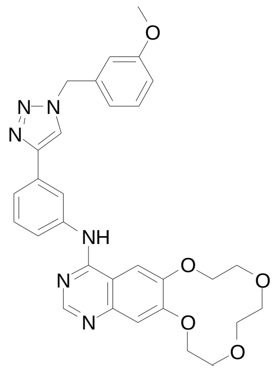

Chemical Formula: $C_{30}H_{30}N_6O_5$
Exact Mass: 554.23

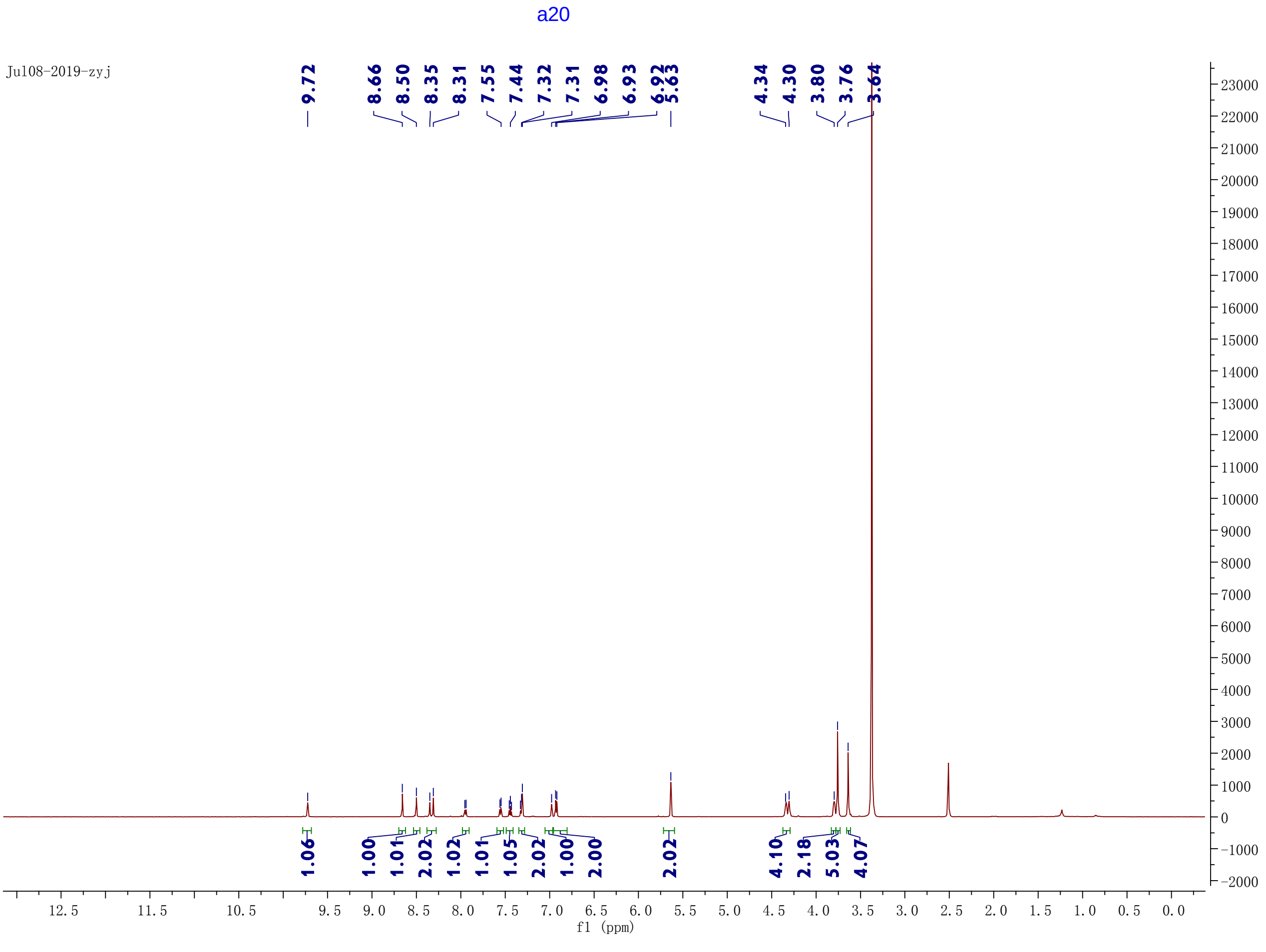

Figure S20-2. ^{13}C NMR spectrum (150MHz, DMSO-d6) of compound a20

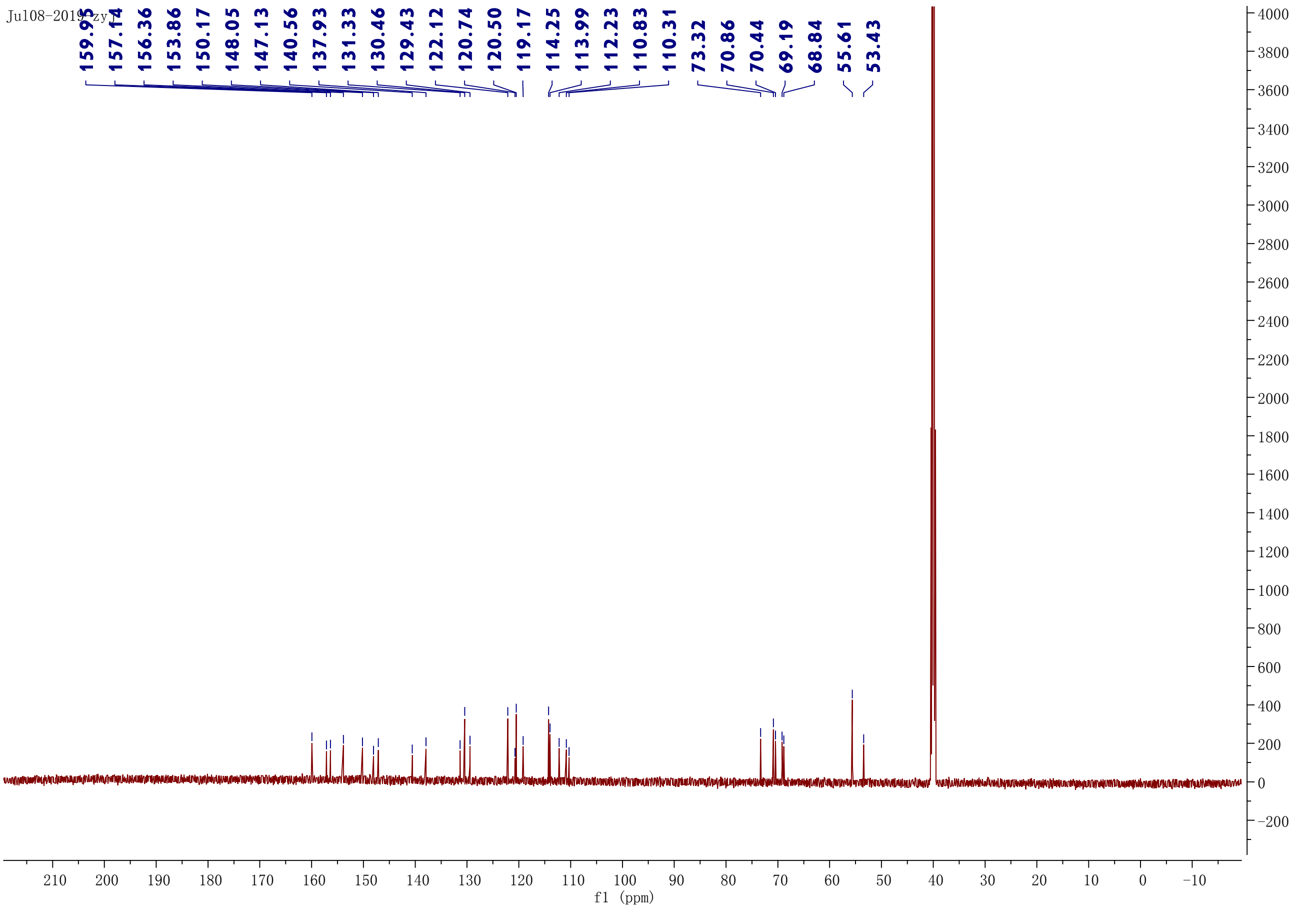

Figure S20-3. HR MS of compound a20

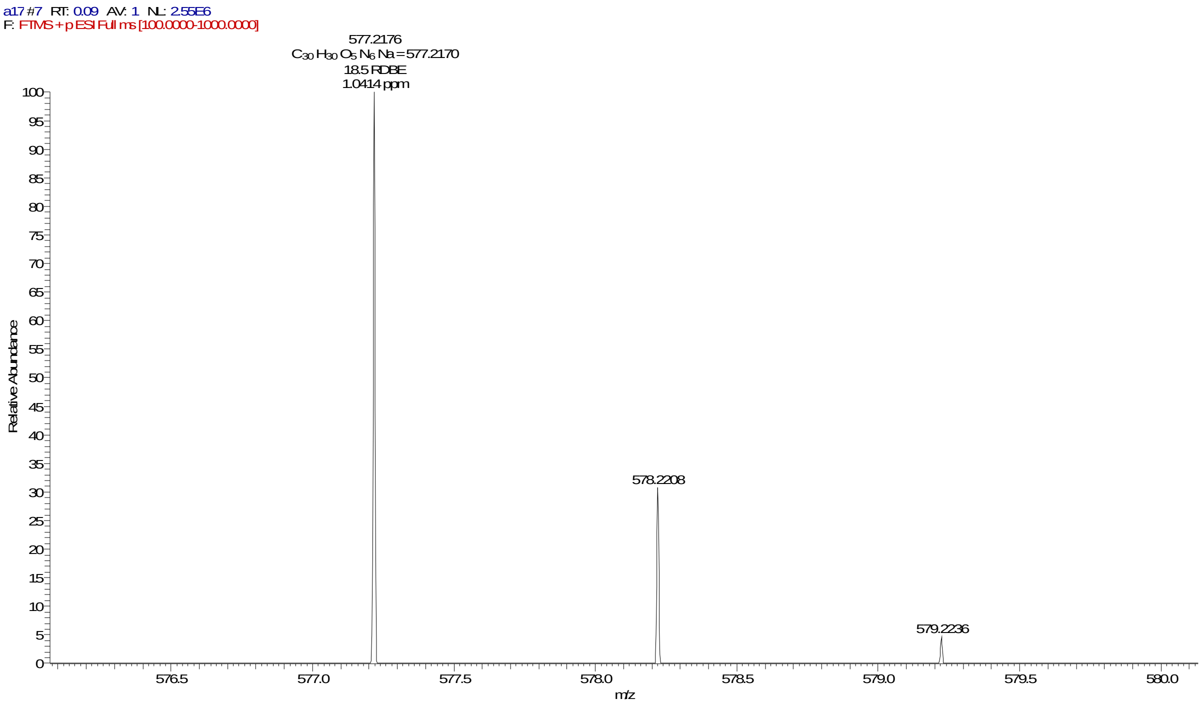

Figure S21-1. ^{1}H NMR spectrum (600MHz, DMSO-d6) of compound a21

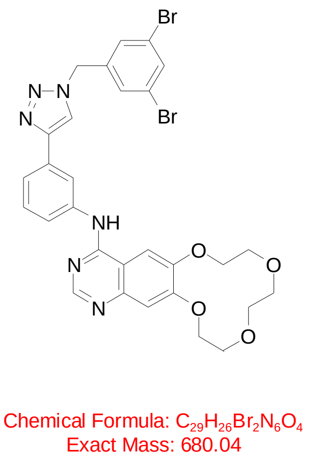

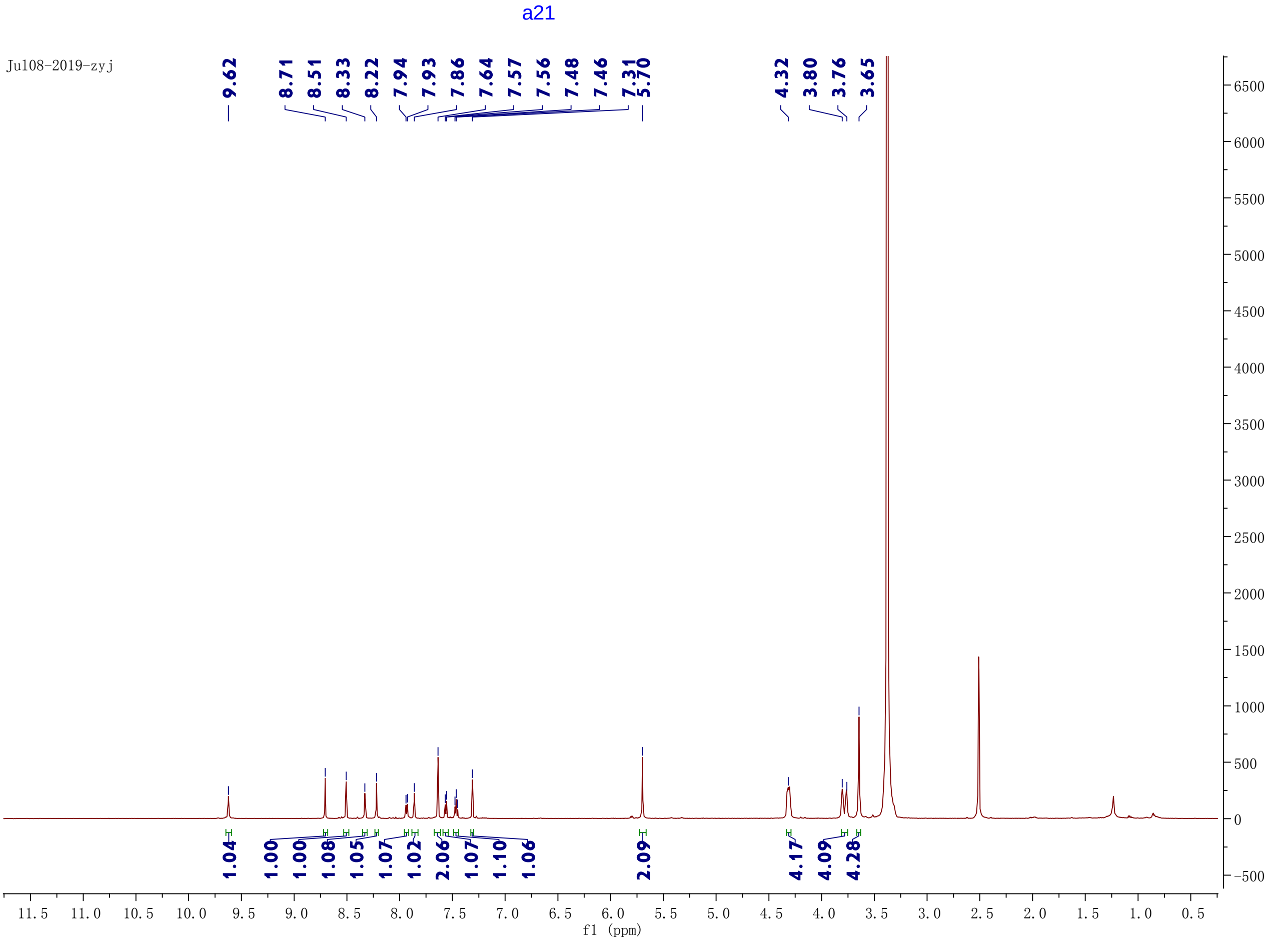

Figure S21-2. ^{13}C NMR spectrum (150MHz, DMSO-d6) of compound a21

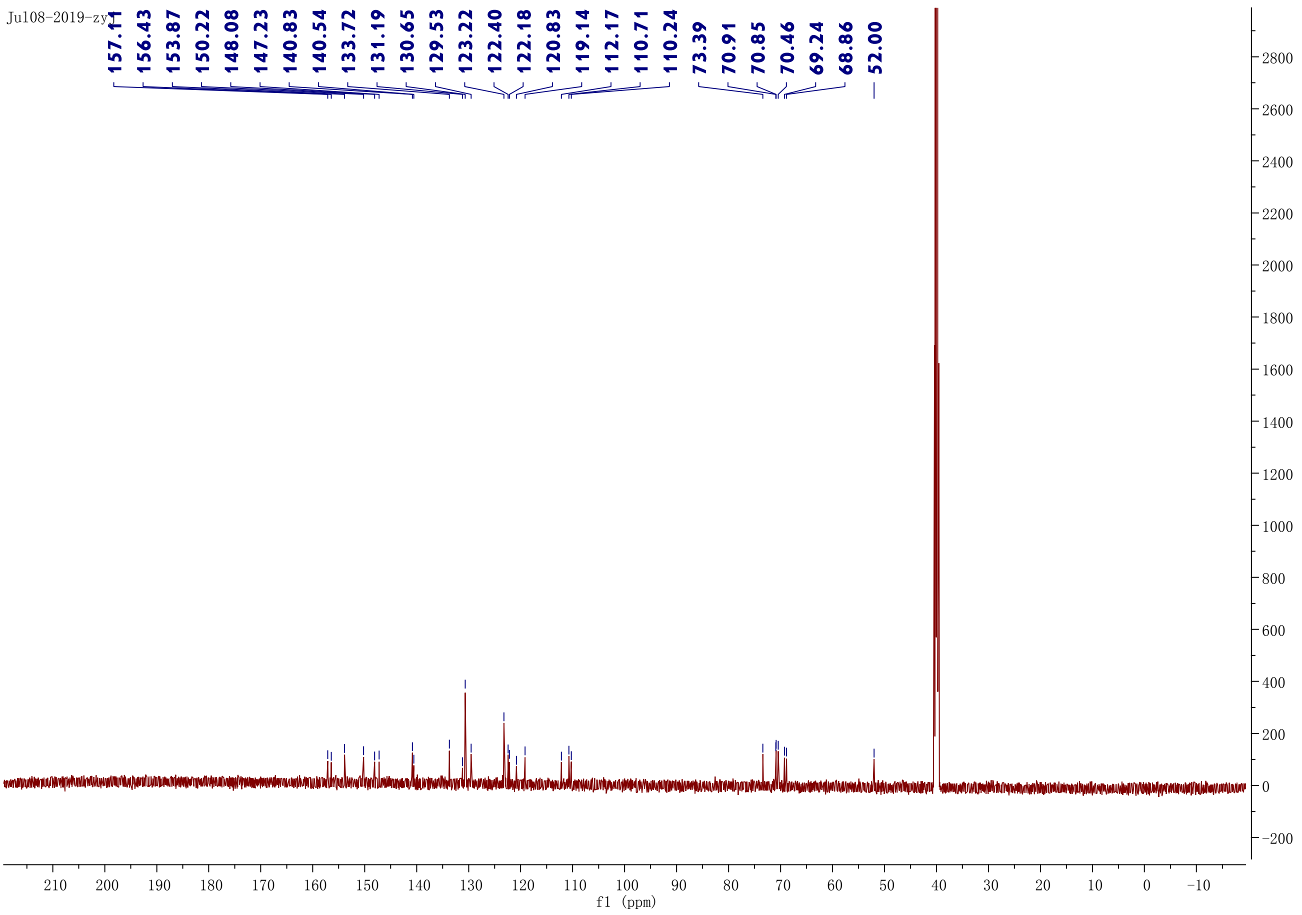

Figure S21-3. HR MS of compound a21

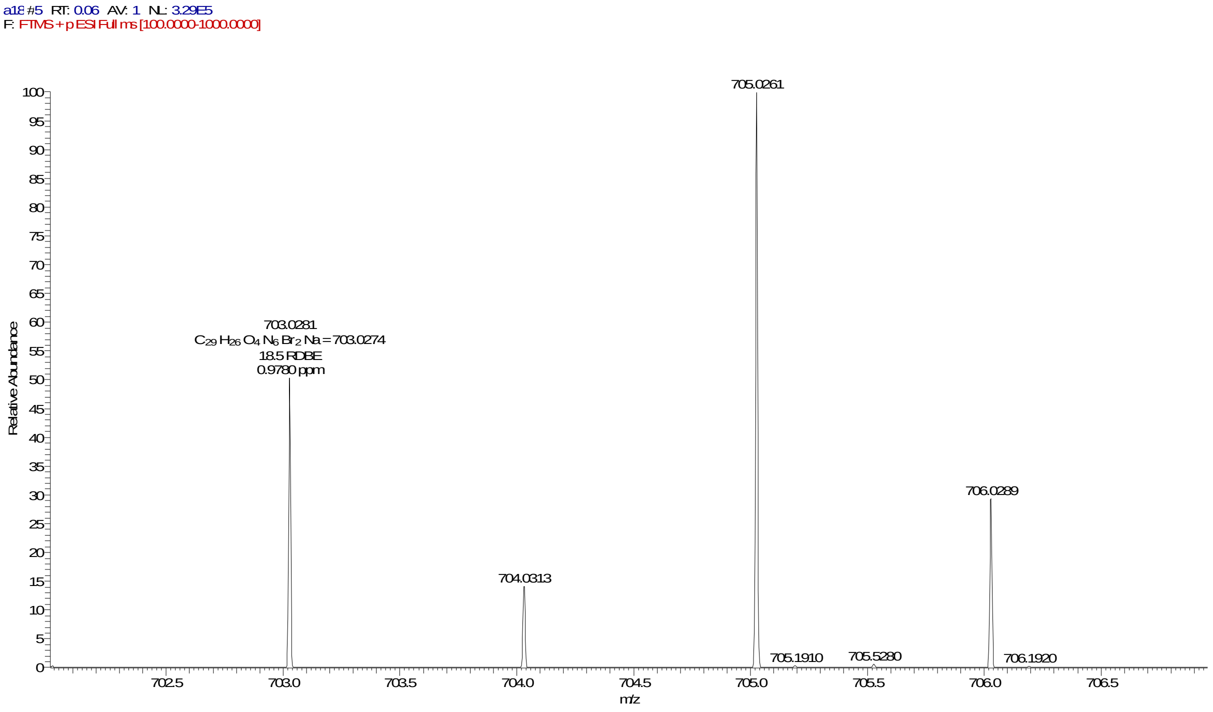

Figure S22-1. ^{1}H NMR spectrum (600MHz, DMSO-d6) of compound a22

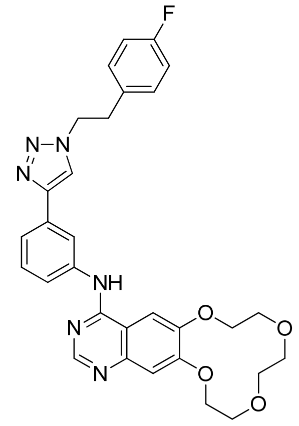

Chemical Formula: $C_{30}H_{29}FN_6O_4$
Exact Mass: 556.22

a22

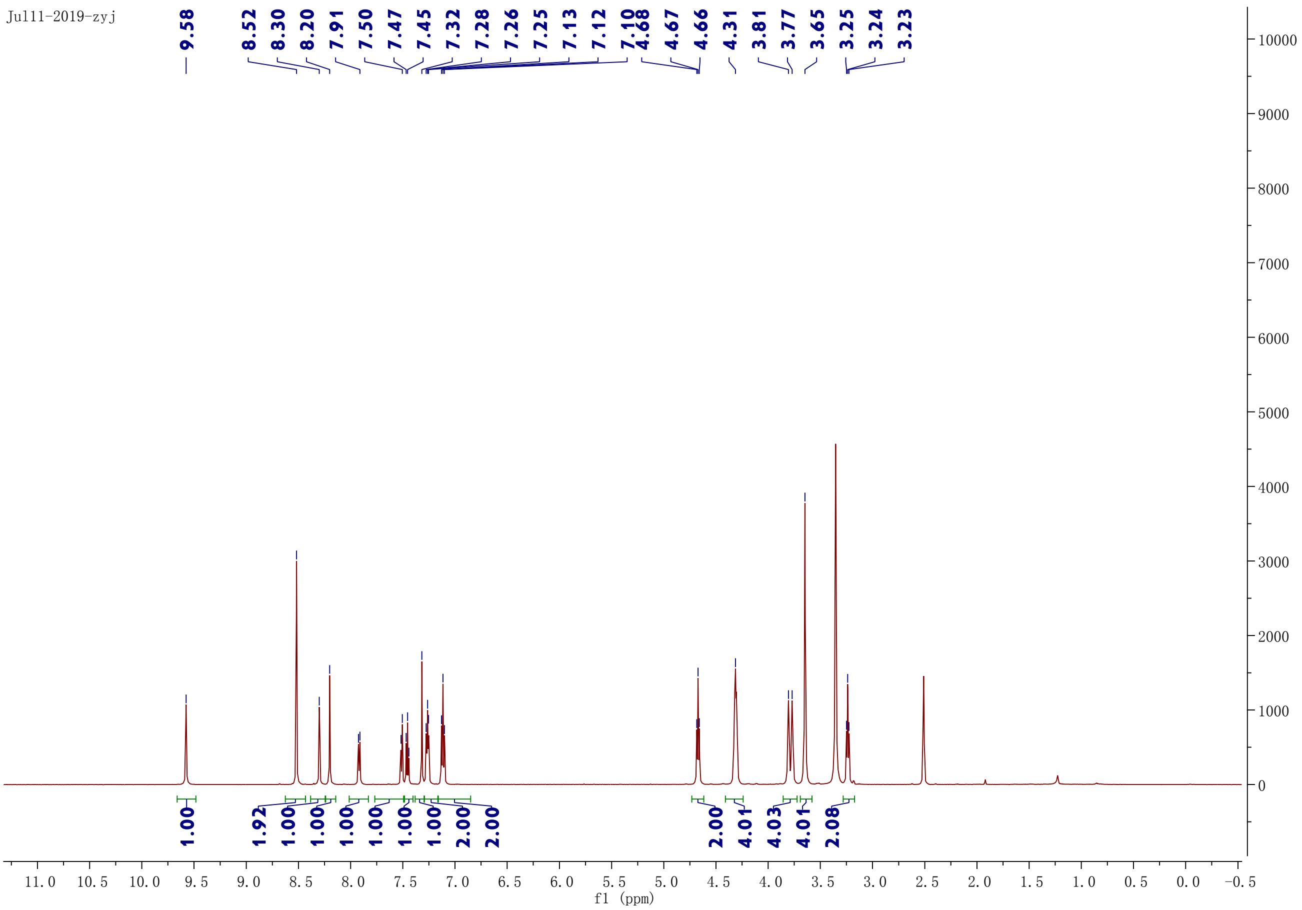

Figure S22-2. ^{13}C NMR spectrum (150MHz, DMSO-d6) of compound a22

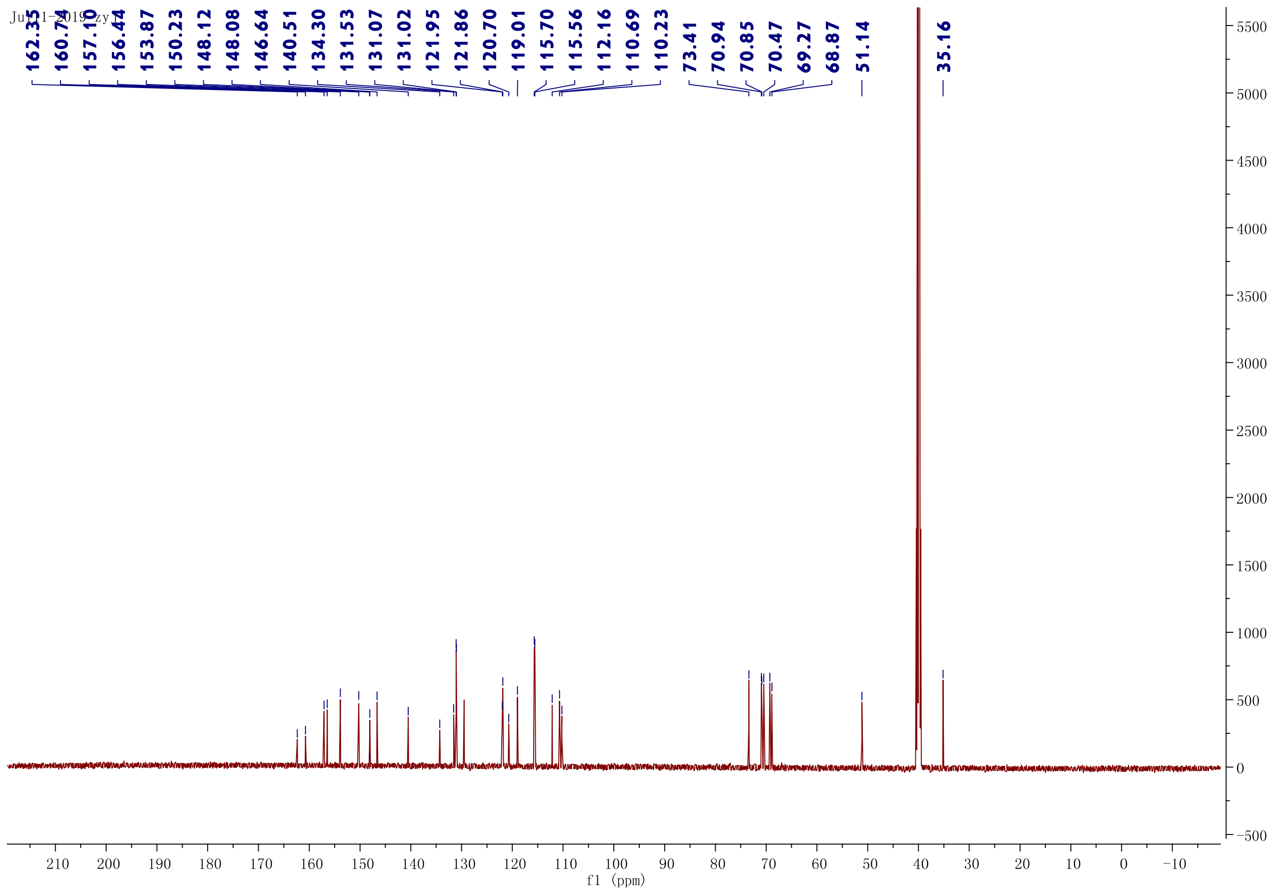

Figure S22-3. HR MS of compound a22

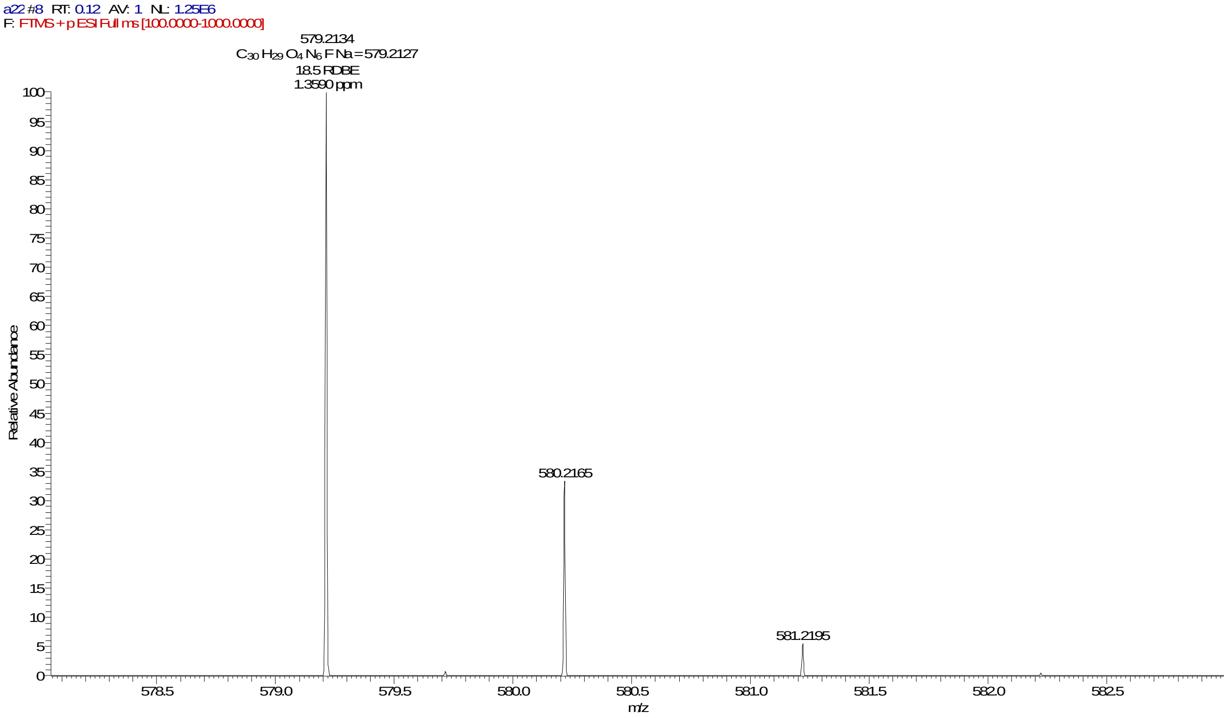

Supplement: Supplementary file 1 [file DataSheet_1.pdf]
